# Highly selective customized reduction products for hydrogenation of CO_2_-derived urea derivatives or carbamates†

**DOI:** 10.1039/d4sc06814a

**Published:** 2024-11-21

**Authors:** Jun Zhu, Yongtao Wang, Jia Yao, Haoran Li

**Affiliations:** a Department of Chemistry, ZJU-NHU United R&D Center, Zhejiang University Hangzhou 310027 China lihr@zju.edu.cn; b State Key Laboratory of Chemical Engineering, College of Chemical and Biological Engineering, Zhejiang University Hangzhou 310027 China

## Abstract

Catalytic hydrogenation of CO_2_-derived urea derivatives or carbamates provides an indirect and efficient solution for the chemical transformation of CO_2_ under mild conditions, avoiding the high temperatures and pressure required for direct catalysis to overcome the thermodynamic energy barrier and the low yield of the targeted product. However, the reported catalyst systems focus mainly on the preparation of one specific product, and switching the product type requires external acid/base additives, which limits the development of this protocol. Here, we report a promising route for the hierarchical reduction of CO_2_-derived urea derivatives or carbamates using an Ir-based PNP pincer catalyst system, enabling the selective production of specific chemicals (methanol, formamides, *N*-methylamines, or *N*,*N*-dimethylamines) for the first time by altering reaction conditions, especially the reaction temperature. This work demonstrates the significant potential of hydrogenation of urea derivatives or carbamates for the indirect conversion of CO_2_ to valuable chemicals and fuels, providing a facile temperature-dependent product-switching strategy in one catalytic system.

## Abstract 2

Selectively customizing hydrogenation products of urea derivatives or carbamates without external acid/base additives remains unrealized. Herein, we report a promising route to selectively obtain formamides, methanol, or methylamines.

## Graphical abstract

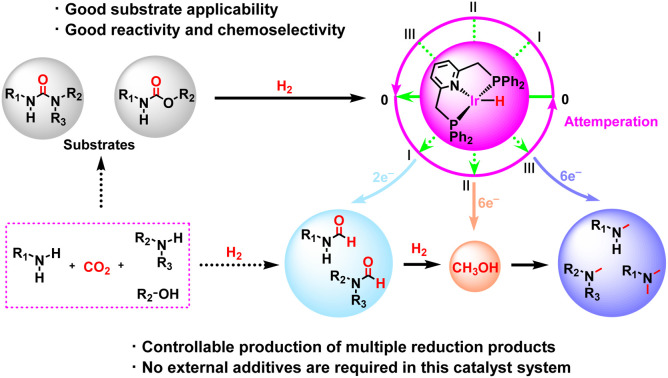

## Introduction

With the acceleration of global industrialization, energy consumption is increasing, and carbon dioxide (CO_2_) emissions are soaring. Whether from the perspective of carbon resource utilization or reducing CO_2_ pollution in the environment, controlling CO_2_ emissions and strengthening the utilization of CO_2_ are of great significance. The resource utilization of CO_2_*via* chemical conversion can not only fix CO_2_, but also produce a variety of valuable fine chemicals to balance the costs associated with CO_2_ capture and conversion.^1–4^ Thus, it is of significant application value to develop reaction routes for converting CO_2_ into energy-storage materials and versatile chemicals under mild reaction conditions.

**Fig. 1:**
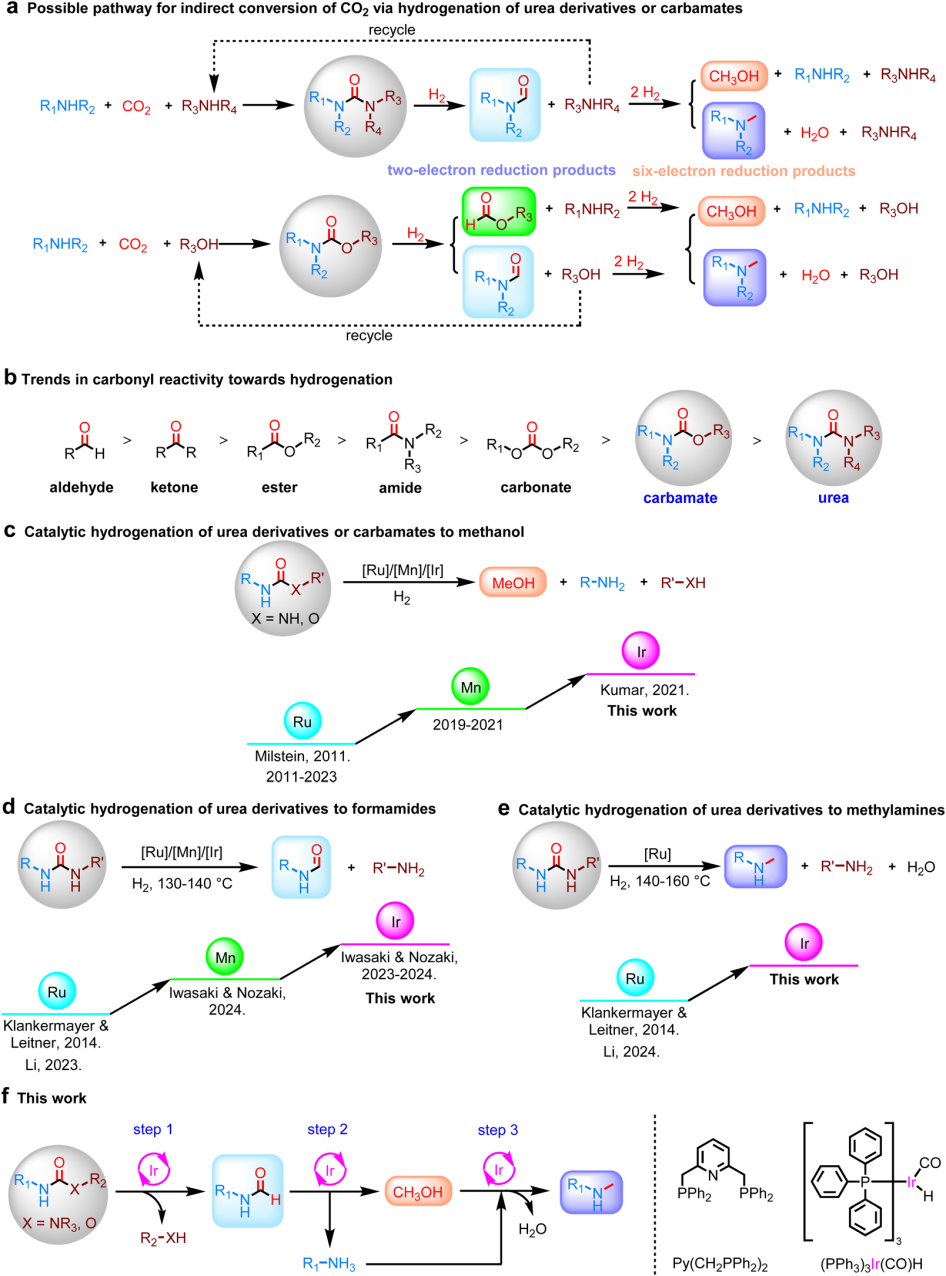
Sustainable alternative routes for the conversion of CO_2_ and amines to formamides, methanol and methylamines based on urea derivatives or carbamates. (a) Indirect conversion of CO_2_ and amines to formamides, methanol and methylamines *via* hydrogenation of carbamates or urea derivatives. (b) Trends in carbonyl reactivity. (c) Previously reported transition-metal catalyst for the hydrogenation of carbamates or urea derivatives to methanol. (d) Previously reported transition-metal catalyst for the hydrogenation of urea derivatives to formamides. (e) Previously reported transition-metal catalyst for the hydrogenation of urea derivatives to methylamines. (f) This work describes the iridium-catalyzed hydrogenation of urea derivatives or carbamates to two- and six-electron reduction products.

Catalytic hydrogenation of CO_2_ is considered to be an attractive method for CO_2_ utilization. The current research mainly focuses on direct and indirect conversion. The direct reduction of CO_2_ is a simple way to produce methanol.^5–9^ The indirect method is used in the presence of alcohols or amines.^10–12^ In recent years, an alternative strategy for CO_2_ reduction has been proposed, which involves indirect hydrogenation of CO_2_*via* the intermediate formation of well-known CO_2_ derivatives, such as formates, formamides, carbonates, carbamates, or urea derivatives.^13–15^ These derivatives are formed upon CO_2_ capture and could be more active than gaseous CO_2_ molecules, thus making subsequent hydrogenation to methanol more effective. Especially, only a few processes using CO_2_ as a C1 source have been industrialized and they are mainly used for the production of urea and its derivatives at present.^16–19^ The reduction of CO_2_-derived urea derivatives or carbamates is thus an alternative approach to expand the resource utilization of CO_2_ (Fig. 1a). In this approach, the first step is to use amine and/or alcohol as a nucleophile to activate and capture CO_2_ to afford a urea derivative or carbamate, which is well-known and thoroughly investigated.^20,21^ The urea derivative or carbamate then undergoes hydrogenation to give methanol and initial amines and/or alcohols, which can be recycled. To realize this protocol in a sustainable manner, routes that enable the highly efficient synthesis of fuels and fine chemicals from urea derivatives or carbamates need to be developed as alternatives to the current synthesis of these chemicals from fossil fuels.

Soon after Milstein and colleagues reported their pioneering work on Ru-catalyzed hydrogenation of urea derivatives to methanol,^22^ in which the two-electron reduction product formamide and an equivalent amine are initially formed by C–N bond cleavage, and then formamide is rapidly hydrogenated to produce methanol without formamide accumulation (Fig. 1c) due to the inherent reactivity order of carbonyl groups (Fig. 1b),^9,13,22–32^ the research groups of Klankermayer and Leitner, Iwasaki and Nozaki, as well as ours, have reported the semi-hydrogenation reduction of urea derivatives to formamides using Ru, Ir, or Mn catalytic systems (Fig. 1d).^31,33–36^ Despite these elegant studies, few studies have reported the hydrogenation of urea derivatives to methylamines (Fig. 1e),^30,31^ and selectively customizing the desired products while precisely controlling the reaction pathways without external additives remains elusive due to the complexity of the process involving the selective cleavage of C–N and C–O bonds.^27,30,35,37–39^ For industrial production, moreover, the ability to produce diverse and variable products from raw materials in response to changes in market demand will become increasingly important. Previously Leigh and colleagues,^40^ as well as Bordet and Leitner,^41^ reported approaches involving artificial switchable catalysis and adaptive catalysis (using temperature or other triggers) respectively. Among them, reaction parameters can be used as a simple and highly effective means to change the chemoselectivity of catalytic reactions because they directly affect the reaction rate without necessarily changing the structure of the catalyst's active site.

Here, we report a promising approach for the orderly hierarchical reduction of CO_2_-derived urea derivatives or carbamates by modulating reaction parameters, enabling the selective production of formamides, methanol, and methylamines (Fig. 1f). To achieve the orderly reduction of urea derivatives or carbamates, the selection of a well-balanced catalyst to subtly control the kinetics of urea derivatives or carbamates reduction is crucial. Previous work showed the selectivity relies heavily on the ligand of the metal catalyst.^35^ Ir metal has been studied less in this reaction system and has relatively mild catalytic hydrogenation capabilities.^28,33,36^ Meanwhile, taking into account the advantage that the tridentate coordination mode of pincer ligands provides strong binding to the metal center, along with easily adjustable steric and electronic properties, we anticipate that the pyridine-based PNP-Ir pincer catalyst system can be used for hydrogenation and dehydrogenation reactions,^39,42–44^ thereby switching the hydrogenation selectivity of urea derivatives or carbamates without external acid/base additives in one catalytic system (Fig. 1f).

## Results and discussion

Our initial studies focused on identifying an Ir-based pincer catalyst capable of converting ureas to formamides and amines, and the hydrogenation of 1,3-bis(4-chlorophenyl)urea was chosen as a benchmark system (Table 1). It is well known that the additional resonance stabilization of alkoxy or amido groups makes ureas the least reactive carbonyl compounds, which makes it extremely challenging to avoid excessive hydrogenation of formamides. To our delight, in the presence of (PPh_3_)_3_Ir(CO)H (1 mol%) and Py(CH_2_PPh_2_)_2_ (1.5 mol%), full conversion of 1,3-bis(4-chlorophenyl)urea was achieved along with the formation of the desired product *N*-(4-chlorophenyl)formamide in 99% yield in tetrahydrofuran (THF) under H_2_ (60 bar) at 140 °C for 8 h (Table 1, entry 1). Based on this result, we attempted to perform this reaction under milder reaction conditions. First, the reaction temperature was gradually reduced (entries 2 and 3). When the reaction temperature was reduced to 120 °C, the reaction efficiency decreased significantly, with only 33% conversion of 1,3-bis(4-chlorophenyl)urea after 12 h (entry 3). Then, the effect of H_2_ pressure on the hydrogenation efficiency was observed at 130 °C (entries 4–6). It is worth noting that 1,3-bis(4-chlorophenyl)urea can still achieve better conversion under a H_2_ pressure as low as 5 bar (entries 6 and 7). Upon investigating various reaction parameters, it was determined that the hydrogenation reaction was most effective in the presence of (PPh_3_)_3_Ir(CO)H (1 mol%) and Py(CH_2_PPh_2_)_2_ (1.5 mol%) at 130 °C under 10 bar H_2_ pressure (entry 8).

**Table 1:** Optimization of the catalytic conditions^a^

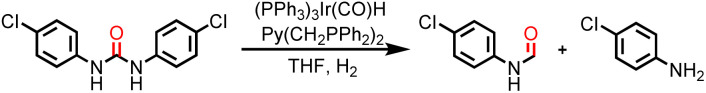					
Entry	H_2_ (bar)	Temperature (°C)	Time (h)	Yield (%) of formamide	Conversion (%)
1	60	140	8	99	>99
2	60	130	12	99	>99
3	60	120	12	32	33
4	30	130	12	98	99
5	10	130	12	96	97
6	5	130	12	80	81
7	5	130	20	88	89
8	10	130	16	99	>99

a Reaction conditions: substrate (2 mmol), (PPh_3_)_3_Ir(CO)H (1 mol%), Py(CH_2_PPh_2_)_2_ (1.5 mol%), THF (4 mL). Determined by GC using biphenyl as an internal standard. Identification of the products was also confirmed by GC-MS and ^1^H NMR. Yields of formamide and amine were reported based on the mole of 1,3-bis(4-chlorophenyl)urea, with a maximum yield of 200%.

**Fig. 2:**
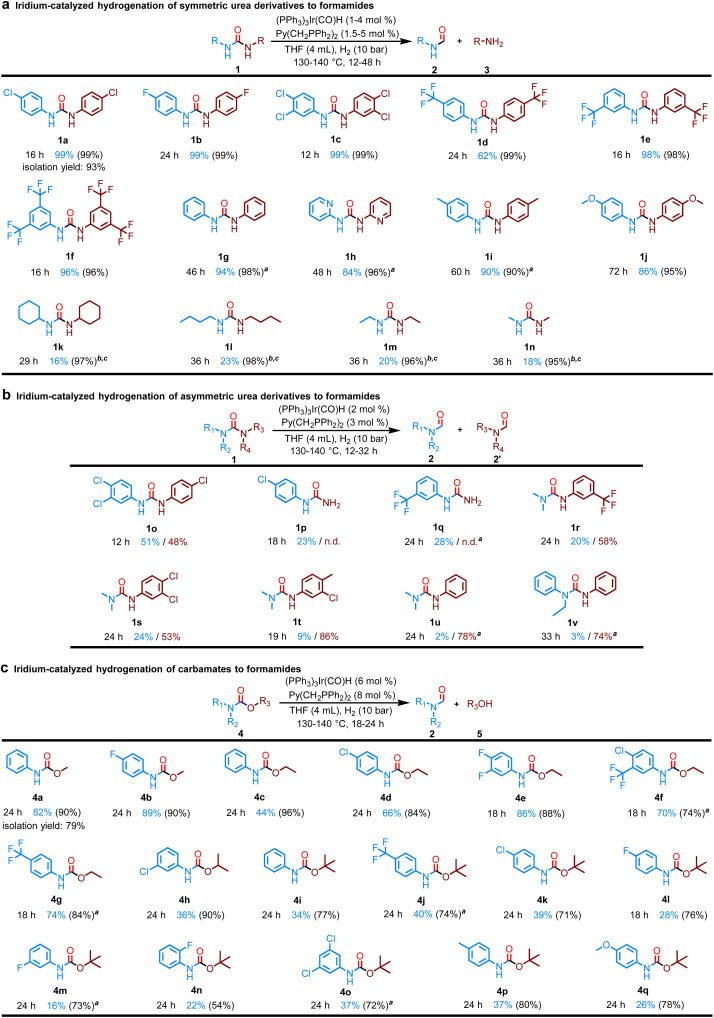
Substrate scope of the hydrogenation of urea derivatives or carbamates. The yield was determined by GC using biphenyl as an internal standard. Identification of the products were also confirmed by GC-MS and ^1^H NMR; selectivity in parentheses. Yields of formamide and amine or alcohol were reported based on the mole of urea derivatives or carbamates, with a maximum yield of 200%; n.d.: not detected. Selectivity value = yield of formamide/(conversion of substrate)×100 (%). (a) Reaction conditions: substrate (2 mmol), (PPh_3_)_3_Ir(CO)H (1 mol%), Py(CH_2_PPh_2_)_2_ (1.5 mol%), H_2_ (10 bar), THF (4 mL), 130 °C (bath temperature). ^*a*^(PPh_3_)_3_Ir(CO)H (2 mol%), Py(CH_2_PPh_2_)_2_ (3 mol%) were used. ^*b*^(PPh_3_)_3_Ir(CO)H (4 mol%), Py(CH_2_PPh_2_)_2_ (5 mol%). ^*c*^140 °C (bath temperature). (b) Reaction conditions: substrate (2 mmol), (PPh_3_)_3_Ir(CO)H (2 mol%), Py(CH_2_PPh_2_)_2_ (3 mol%), H_2_ (10 bar), THF (4 mL), 130 °C (bath temperature). ^*a*^140 °C (bath temperature). (c) Reaction conditions: substrate (1 mmol), (PPh_3_)_3_Ir(CO)H (6 mol%), Py(CH_2_PPh_2_)_2_ (8 mol%), H_2_ (10 bar), THF (4 mL), 140 °C (bath temperature). ^*a*^130 °C (bath temperature).

Encouraged by this result, the hydrogenation of various symmetric urea derivatives bearing electron-withdrawing or electron-donating substituents was investigated in more detail (Fig. 2a). As expected, urea derivatives with electron-withdrawing groups such as F, CF_3_, or Cl at different substitution sites on aniline were efficiently converted (1a–f). The conversion of 1,3-diphenylurea without any substituent on the aniline ring was 96% and the yield of formanilide was 94% in the presence of (PPh_3_)_3_Ir(CO)H (2 mol%) and Py(CH_2_PPh_2_)_2_ (3 mol%) after 46 h (1g). 1,3-di(pyridin-2-yl)urea, in which the benzene ring is replaced with pyridine, was also highly selectively converted to *N*-(pyridin-2-yl)formamide (1h). Electron-donating groups such as Me or OMe at the *para*-position of the aniline reduced the conversion efficiency, but did not affect the selectivity (1i and 1j). We then moved on to various alkyl urea derivatives. It is worth noting that various alkyl urea derivatives were also successfully converted using the Ir-based catalyst system (1k–n).

Following the successful hydrogenation of symmetrical urea derivatives, we tried to use the Ir catalyst system for the catalytic hydrogenation of asymmetric urea derivatives. Gratifyingly, two distinct formamide products can be clearly observed in Fig. 2b. Thus, the first C–N bond cleavage shows clear regioselectivity in unsymmetric ureas. Even the more sterically hindered tri-substituted urea derivatives were selectively hydrogenated to amines along with the corresponding mono-substituted formamide and di-substituted formamides. The yield of di-substituted formamides was significantly lower than that of mono-substituted formamides, which may be due to the steric hindrance caused by adjacent *N*-Me or *N*-Et groups. Noticeably, tetra-substituted urea derivatives are difficult to hydrogenate under these similar catalytic conditions.

After successful hydrogenation of the most hydrogenation-resistant carbonyl compounds, especially tri-substituted urea derivatives, we next turned our attention to the more challenging catalytic hydrogenation of carbamates to formamides. This is because Carbamates present difficulties in achieving selectivity between dealcoholization hydrogenation (C–O bond cleavage) and deaminative hydrogenation (C–N bond cleavage). Gratifyingly, using (PPh_3_)_3_Ir(CO)H (6 mol%) and Py(CH_2_PPh_2_)_2_ (8 mol%) under H_2_ (10 bar) at 140 °C for 24 h in THF, various carbamates bearing aliphatic or aromatic substituents were eventually highly chemoselectively hydrogenated into formamides (Fig. 2c).

**Scheme 1:**
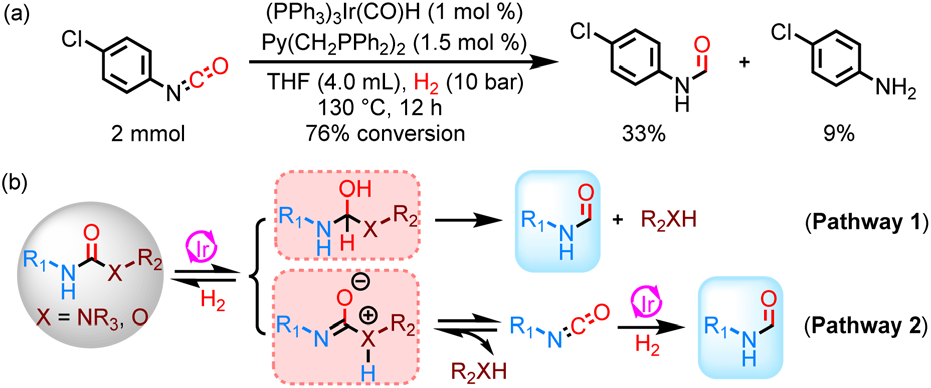
Study of the reaction pathway for hydrogenation of urea derivatives or carbamates to formamides. (a) Catalytic hydrogenation of 4-chlorophenyl isocyanate. (b) Reaction pathways for the hydrogenation of urea derivatives or carbamates to formamides.

In this study, di-substituted formamides were obtained by catalytic hydrogenation of tri-substituted urea derivatives in a selective manner. Similarly, formanilide and methyl formate were detected during the hydrogenation of methyl *N*-phenylcarbamate. These results indicate that the reaction proceeds through pathway 1. However, reaction pathway 2 cannot be ruled out. Previously, our group and Nozaki *et al.* reported that urea derivatives can be slowly pyrolyzed to form isocyanates and corresponding amines at 130–140 °C.^33,34^ Consequently, there are two pathways (Scheme 1b) for the hydrogenation of urea derivatives to formamides: (1) the carbonyl C=O double bond hydrogenation forms a hemiaminal intermediate, which selectively generates amine and formamide (pathway 1); (2) the urea derivative undergoes thermal decomposition into isocyanate, which is then hydrogenated to formamide (pathway 2).^30,33,34^ Moreover, 4-chlorophenyl isocyanate can be hydrogenated to 4-Cl-phenylformamide under the same catalytic conditions, but the hydrogenation efficiency is significantly lower than that of 1,3-bis(4-chlorophenyl)urea (Scheme 1a). Meanwhile, the conversion efficiency of 1,3-bis(4-chlorophenyl)urea was very low at 130 °C for 12 h in a N_2_ environment. Thus, hydrogenation of urea derivatives or carbamates to formamides is mainly carried out *via* reaction pathway 1.

**Scheme 2:**
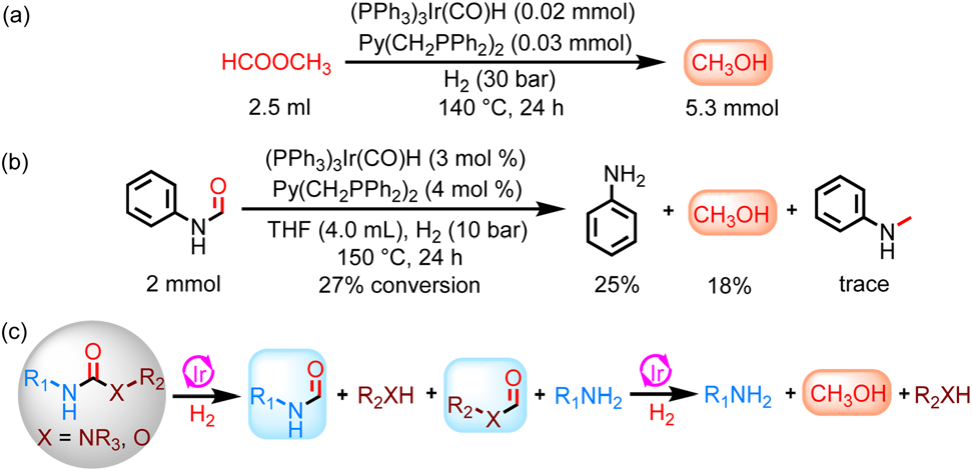
Study of the reaction pathway for the hydrogenation of urea derivatives or carbamates to formamides. (a) Catalytic hydrogenation of methyl formate. (b) Catalytic hydrogenation of formanilide. (c) Reaction pathways for the hydrogenation of urea derivatives or carbamates to methanol.

**Fig. 3:**
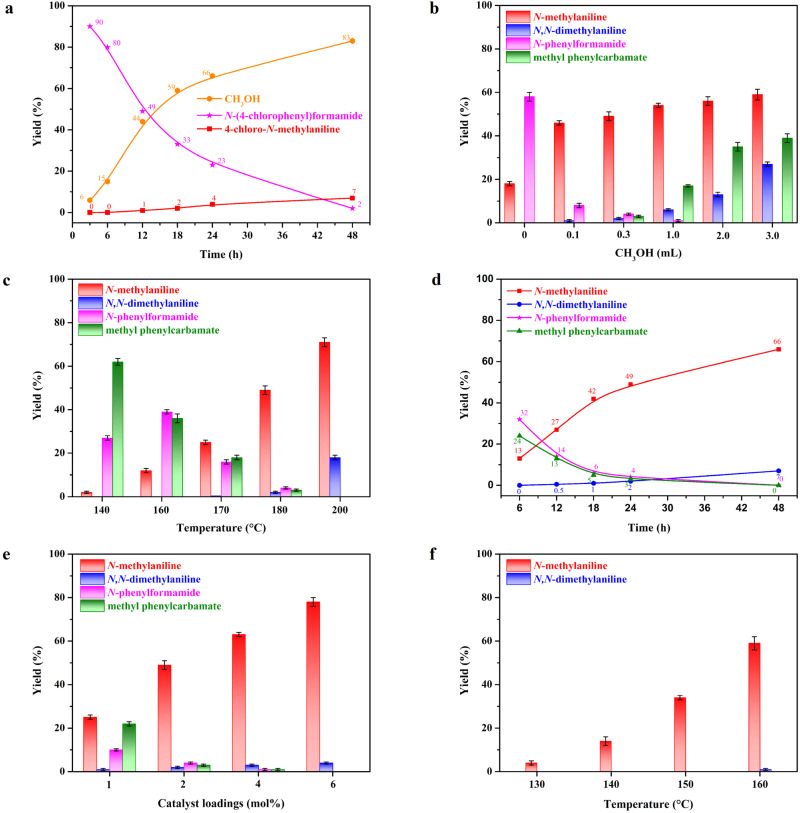
Catalytic hydrogenation of 1,3-diphenylurea to *N*-methylaniline and *N*,*N*-dimethylaniline. Reaction conditions: 1,3-diphenylurea (2 mmol), (PPh_3_)_3_Ir(CO)H (2 mol%), Py(CH_2_PPh_2_)_2_ (3 mol%), H_2_ (30 bar), solvent (4 mL), 180 °C (bath temperature). The yield was determined by GC using biphenyl as an internal standard. Identification of the products were also confirmed by GC-MS and ^1^H NMR. (a) Reaction conditions: 1,3-bis(4-chlorophenyl)urea (2 mmol), (PPh_3_)_3_Ir(CO)H (4 mol%), Py(CH_2_PPh_2_)_2_ (5 mol%), H_2_ (30 bar), 150 °C, THF (4 mL). (b) Reaction conditions: solvent (0.3 mL methanol and 3.7 mL THF), reaction temperatures (140–200 °C), reaction time (24 h). (c) Reaction conditions: solvent (0.3 mL methanol and 3.7 mL THF), reaction temperature (180 °C), reaction times (6–48 h). (d) Reaction conditions: solvent (0.3 mL methanol and 3.7 mL THF), reaction temperature (180 °C), reaction times (6–48 h). (e) Reaction conditions: 1,3-diphenylurea (2 mmol), (PPh_3_)_3_Ir(CO)H (1–6 mol%), Py(CH_2_PPh_2_)_2_ (1.5–9 mol%), solvent (0.3 mL methanol and 3.7 mL THF), reaction temperature (180 °C), reaction time (24 h). (f) Reaction conditions: aniline (4 mmol), (PPh_3_)_3_Ir(CO)H (1 mol%), Py(CH_2_PPh_2_)_2_ (1.5 mol%), N_2_ (30 bar), solvent (0.3 mL methanol and 3.7 mL THF), reaction temperatures (130–160 °C), reaction time (20 h).

Interestingly, methanol was observed in addition to ethanol in the catalytic hydrogenation of ethyl phenylcarbamate, which aroused our great research interest in determining the source of methanol. It may provide powerful insights into the further catalytic hydrogenation of urea derivatives or carbamates to methanol using the Ir-based catalyst system. Catalytic hydrogenation of formanilide at 140 °C for 24 h resulted in an 18% yield of methanol (Scheme 2b), confirming that formamides are one of the sources of methanol. Moreover, a small amount of methyl formate was detected in the hydrogenation of methyl *N*-phenylcarbamate, and methyl formate could be hydrogenated to methanol using this catalyst system, suggesting that it may be another source of methanol (Scheme 2a). Consequently, complete hydrogenation of carbamates can yield methanol regardless of whether the C–N bond cleavage or the C–O bond cleavage occurs first. For the hydrogenation of urea derivatives to methanol, ureas are first hydrogenated to formamides, which are then fully hydrogenated to methanol.^22,26^ More specifically, the hydrogenation rate of urea derivatives or carbamates to formamides is much faster than the subsequent hydrogenation of formamides in this Ir-based PNP pincer catalyst system (Fig. 3a). Thus, methanol can be obtained with high selectivity by optimizing reaction conditions (Table S1†), and this catalyst system can be applied to various types of carbonyl substrates, including formamides, carbamates, esters, and urea derivatives (Table S2†).

**Scheme 3:**
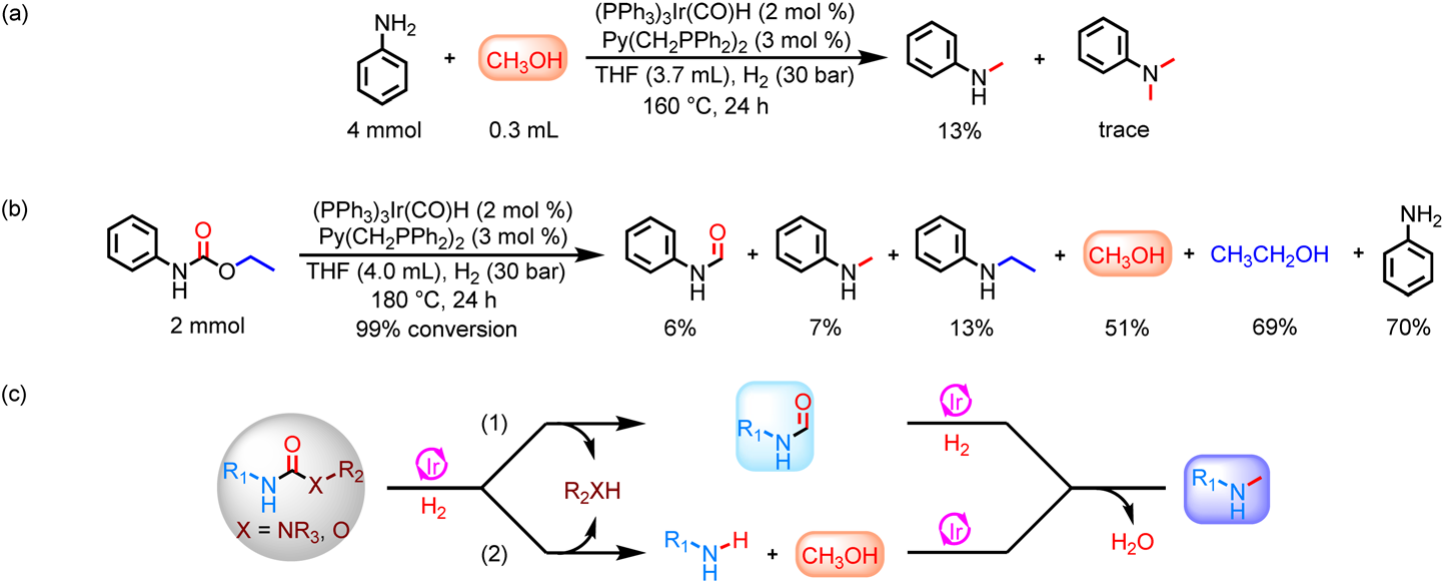
Study of the reaction pathway for hydrogenation of urea derivatives or carbamates to *N*-methylamines. (a) Catalytic coupling of aniline and methanol. (b) Catalytic hydrogenation of ethyl phenylcarbamate. (c) Reaction pathways for hydrogenation of urea derivatives or carbamates to *N*-methylamines.

More interestingly, the hydrogenation of methyl *N*-phenylcarbamate and 1,3-diphenylurea at 160 °C for 24 h showed the presence of methylaniline in addition to formanilide and methanol (Table S1 and Fig. S6†). When the reaction temperature was increased to 180 °C and 200 °C for the same reaction duration, the yield of formanilide significantly decreased, while the yield of methylaniline increased (Fig. S4†). Based on the above results, it can be preliminarily concluded that increasing the reaction temperature helps catalyze the hydrogenation of urea derivatives or carbamates to produce six-electron reduction products, especially methylamine. To investigate the origin of the methyl group in methylaniline, hydrogenation of formanilide and *N*-alkylation of methanol and aniline were performed (Schemes 2b and 3a). These experimental results show that both reaction pathways are feasible in the Ir-based catalyst system. Moreover, ethylaniline with 13% yield was also detected in addition to *N*-methylaniline in the ethyl phenylcarbamate hydrogenation reaction at 180 °C for 24 h, which further proves that methyl groups can be derived from the catalytic coupling of methanol and amines, as ethylaniline can only be obtained through the dehydrogenative coupling of aniline and the resulting ethanol (Scheme 3b). Thus, there are two major reaction routes for hydrogenation of urea derivatives or carbamates to methylamines: (1) hydrodehydration of formamide intermediates; (2) catalytic coupling of methanol and amines (Scheme 3c).

Remarkable progress has been achieved in the highly selective tuning of formamide and methanol products in this catalyst system, but regulating the selectivity of methylamine products remains a challenge because this six-electron reduction process is accompanied by the formation of methanol byproducts. To our delight, the successful dehydrogenative coupling of alcohols and amines in Ir-based catalyst system provides us with a new design concept, which means that we can improve the selectivity of methylamines by adding methanol as a co-solvent to accelerate the reaction of methanol and amines (methanol can be obtained from the hydrogenation of urea derivatives or carbamates). Notably, *N*-methylaniline with 49% yield was obtained at 180 °C for 24 h in the presence of 0.3 mL methanol, which is similar to the yield achieved from the full conversion of 1,3-diphenylurea at 200 °C (Fig. S6†). The addition of methanol likely accelerates the reaction rate for coupling of methanol and amine, while also inhibiting the hydrogenation of formamide to methanol, thus increasing the formation rate of methylamine. Therefore, this approach is feasible for obtaining *N*-methylamine products with high yield.

**Fig. 4:**
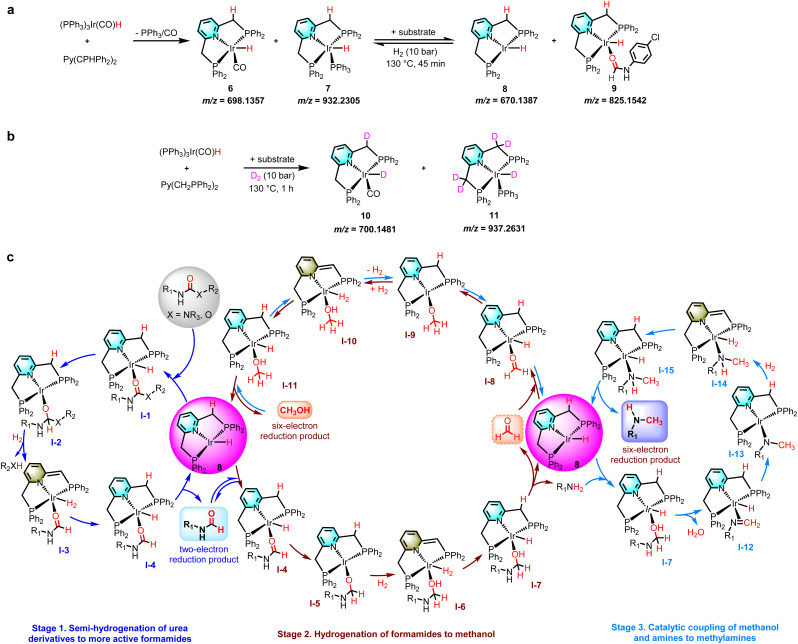
Mechanistic studies. (a) Study of the catalytically active species. (b) Deuterium labeling study. (c) Proposed reaction mechanism for the iridium-catalyzed hydrogenation of carbamates or urea derivatives to formamides, methanol, and methylamines.

To further optimize the chemoselectivity for methylamine, the effects of reaction parameters were investigated. First, we finely modulated the proportion of methanol in the solvent. The formation of *N*-methylaniline is favored in the presence of a lower concentration of methanol. In contrast, *N*-methylaniline and methanol were further coupled to produce *N*,*N*-dimethylaniline in the presence of a higher concentration of methanol (Fig. 3b). Subsequently, the effect of reaction temperature and reaction time were explored. As expected, the yields of *N*-methylaniline and *N*,*N*-dimethylaniline increased with the increase in reaction time and temperature (Fig. 3c and 4d). It is worth noting that methyl *N*-phenylcarbamate is formed in high yield at a short reaction time, and methyl *N*-phenylcarbamate is gradually consumed with the extension of the reaction time. This indicates that 1,3-diphenylurea first reacts with methanol to form methyl *N*-phenylcarbamate (Fig. 3b–e), which is then hydrogenated to methanol, methylaniline and aniline. As the reaction time was prolonged, methanol and aniline continued to react, leading to an improvement in the yield of methylaniline. Similarly, increasing the catalyst loading also accelerates the reaction rate (Fig. 3e). Thus, we can customize *N*-methylamines and *N*,*N*-dimethylamines by changing the ratio of methanol in the solvent. Moreover, optimizing experimental parameters such as reaction temperature and catalyst loading improves the conversion efficiency.

**Scheme 4:**
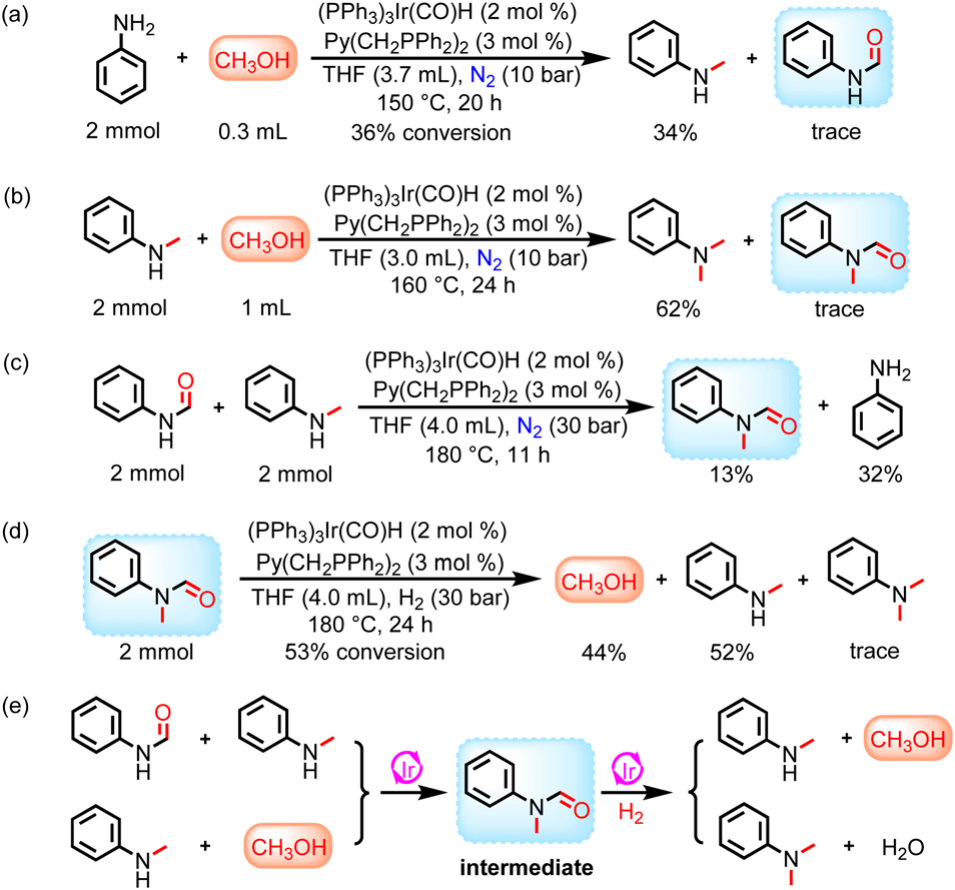
Study of the reaction pathway for hydrogenation of urea derivatives or carbamates to *N*-methylamines and *N*,*N*-dimethylamines in the presence of methanol. (a) Catalytic coupling of aniline and methanol. (b) Catalytic coupling of *N*-methylaniline and methanol. (c) Catalytic coupling of formanilide and *N*-methylaniline. (d) Catalytic hydrogenation of *N*-methyl-*N*-phenylformamide. (e) Reaction pathways for hydrogenation of urea derivatives or carbamates to *N*-methylamines and *N*,*N*-dimethylamines in the presence of methanol.

After solving the selectivity issue of methylamines, we turned our attention to exploring the reaction routes for the catalytic coupling of methanol and amines to produce methylated products. When aniline/methylaniline reacted with methanol in a N_2_ environment, considerable amounts of *N*-methylaniline/*N*,*N*-dimethylaniline products were observed (Schemes 4a and 4b). In addition, trace amounts of formanilide/*N*-methyl-*N*-phenylformamide intermediates were also detected. These results demonstrate that methanol and aniline/methylaniline were first dehydrogenated to formanilide/*N*-methyl-*N*-phenylformamide intermediates,^45–51^ followed by rapid hydrodehydration to produce *N*-methylaniline/*N*,*N*-dimethylaniline.^42,52–57^ Meanwhile, formanilide and *N*-methylaniline also react to produce the *N*-methyl-*N*-phenylformamide intermediate (Scheme 4c).^58,59^ Similarly, the catalytic hydrogenation of *N*-methyl-*N*-phenylformamide intermediate proceeds through two reaction pathways: (a) leading to *N*-methylaniline and methanol; (b) leading to *N*,*N*-dimethylaniline by dehydration (Scheme 4d and 4e).

The catalytic coupling of methanol and aniline under a N_2_ atmosphere shows that the Ir-based catalyst system also has a good catalytic coupling effect in addition to excellent catalytic hydrogenation ability in this reaction. Based on this, we further investigated the effect of reaction temperature on catalytic coupling of methanol and aniline in the N_2_ environment. Notably, dehydrogenative coupling of methanol and amines can be achieved at a lower reaction temperature (Fig. 3f). Therefore, it is an ideal route for the conversion of urea derivatives or carbamates to methylamines under more mild reaction conditions (reaction temperature as low as 140 °C) by a two-step process. That is, urea derivatives or carbamates are first hydrogenated in a H_2_ environment to produce methanol and amines, and then the resulting methanol and amines are catalytically coupled under a N_2_ atmosphere to finally obtain the targeted products (Fig. S29†).

After studying the possible reaction routes involved in the hydrogenation of carbamates or urea derivatives, we turned our attention to the mechanistic details regarding the speciation of the catalytically active species. The results of ESI-MS (*m*/*z* = 698.1357 and 932.2305) and the presence of PPh_3_ in the reaction solution indicate that the pincer ligand Py(CH_2_PPh_2_)_2_ reacts with (PPh_3_)_3_Ir(CO)H in THF under a N_2_ atmosphere to produce 18-electron complexes 6 and 7 (Fig. 4a). Performing the same experiment in a H_2_ environment, 16-electron complex 8 (*m*/*z* = 670.1396) was detected in addition to complexes 6 and 7 (see the ESI† for more details). Subsequently, the 1,3-bis(4-chlorophenyl)urea substrate was added to the analogous reaction. Complex 8 (*m*/*z* = 670.1387) and compound 9 (*m*/*z* = 825.1542) formed by the coordination of *N*-(4-chlorophenyl)formamide to complex 8 were observed in the reaction solution with incomplete substrate conversion (Fig. 4a). These results suggest that the 16-electron complex 8 may be the catalytically active substance for the hydrogenation of carbamates or urea derivatives. Complexes 6 and 7 first remove a CO/PPh_3_ ligand from the more stable 18-electron catalyst precursors 6 and 7, creating the 16-electron catalytically active substance 8, which then coordinates with the substrate and enters the catalytic cycle. To further explore the reaction mechanism, we performed deuteration labeling experiments with D_2_ instead of H_2_ (Fig. 4b). According to ^1^H NMR spectroscopic characterization and ESI-MS analysis, D-labels are incorporated into Ir-D and partial H/D exchange occurs in the pincer arm methylene. Complex 10 (*m*/*z* = 700.1481) and complex 11 (*m*/*z* = 937.2631) were detected in the reaction solution at 130 °C after 1 h under a D_2_ atmosphere, while complexes 6 and 7 were not detected, indicating that complexes 10 and 11 were generated by the reaction of complexes 6 and 7 with D_2_, respectively. When the reaction time was prolonged to 6 h (with incomplete substrate conversion), further transformation of complex 10 into 11 was observed (*m*/*z* = 701.153). Therefore, this hydrogenation of carbamates or urea derivatives may be carried out *via* metal–ligand cooperativity.^43,60–65^

Considering our experimental results, and previous reports on catalytic hydrogenation of urea derivatives or carbamates and catalytic coupling of alcohols and amines,^13,26,31,42,52^ we propose a reasonable catalytic cycle for hydrogenation of carbamates or urea derivatives to two- and six-electron reduction products (Fig. 4c, see the SI for more details). The precatalyst 6/7 is prepared *in situ* by releasing PPh_3_/CO through dissociative exchange, using the Py(CH_2_PPh_2_)_2_ ligand and (PPh_3_)_3_Ir(CO)H metal precursor as raw materials. Then the PPh_3_/CO ligand dissociates from the 6/7 complex to form the 16-electron complex 8 at the reaction temperature. Since the catalytically active species are formed *in situ* at the initial stage of the reaction, a distinct induction period was observed in most of the presented time profiles (Fig. 3). At the start of the catalytic cycle, the urea derivative or carbamate coordinates with complex 8 to form substrate complex I-1, which then undergoes a migratory insertion step to generate complex I-2. The latter removes R_2_XH (X = NR_3_, O) by metal–ligand cooperation to form complex I-3, which regenerates complex 8 by releasing *N*-formamide. By adjusting the reaction parameters to accelerate the reaction rate, *N*-formamide will enter the next catalytic cycle and undergo hydrogenation reaction to produce methanol. Similarly, *N*-formamide reacts with complex 8 to form complex I-4, which eliminates the amine by metal−ligand cooperation to form formaldehyde complex I-8. The complex I-8 activates H_2_ by metal–ligand cooperation to form the methoxy complex I-11. Elimination of methanol from I-11 regenerates complex 8. In addition, the catalytically active substance 8 shows good dehydrogenation coupling performance and can further catalyze the coupling reaction between methanol and amine to produce methylamine. This stage requires a higher reaction temperature or a N_2_ environment because the reaction rate is inhibited in the H_2_ environment. First, methanol combines with complex 8 to form the I-11 complex. The I-11 complex undergoes reverse cycling for dehydrogenation (I-8) and couples with the amine to produce complex I-7. The latter then generates complex I-12 by eliminating H_2_O. The neighbouring H is transplanted and inserted to form complex I-13 by migratory insertion, and complex I-13 is coordinated with hydrogen to generate I-14. Finally, complex I-15 releases methylamine to regenerate the catalytically active species 8.

## Conclusions

In summary, we report an effective and universal strategy for precisely customizing reduction products *via* a sequential reaction process for the hydrogenation of carbamates or urea derivatives using a PNP pincer-type iridium complex in the absence of acid/base additives, involving 2-electron reduction, 6-electron reduction, and dehydrogenative coupling. In neat THF solvent, highly selective hydrogenation of carbamates or urea derivatives into formamides is achieved at lower reaction temperatures (up to 99% selectivity at 130 °C). With an increase in reaction temperature (from 130 to 150 °C), formamides are further hydrogenated to produce 6-electron reduction products, especially methanol (up to 84% yield). As the reaction temperature continues to increase, the resulting methanol and amines undergo a dehydrogenation coupling reaction to form methylamines. More importantly, the selectivity of methylamines is improved by optimizing the reaction conditions with methanol as a co-solvent (up to 93% selectivity at 180 °C, methanol can be obtained by total hydrogenation of urea derivatives or carbamates). Switching the product type for the hydrogenation of carbamates or urea derivatives based on the dual roles of hierarchical hydrogenation and dehydrogenative coupling of the Ir-based catalyst system is unprecedented, which opens the possibility of selectively customizing the desired products and precisely controlling the reaction pathways without external additives. This work demonstrates the great potential of hydrogenating urea derivatives or carbamates for the indirect conversion of CO_2_ and for the production of fuels and valuable fine chemicals, providing a new perspective for selective and total hydrogenation of urea derivatives or carbamates in one catalytic system.

## Data availability

All data needed to evaluate the conclusions in the paper are present in the paper and/or the ESI.† And all data can be obtained from the authors.

## Author contributions

H. L. conceived the project and supervised the work with Y. W. and J. Y. J. Z. performed the catalysis experiments and wrote the manuscript. All authors discussed the results and contributed to the final manuscript.

## Conflicts of interest

There are no conflicts to declare.

## Supplementary Material

SC-OLF-D4SC06814A-s001
