# Supplementary material for: Highly selective customized reduction products for hydrogenation of CO2-derived urea derivatives or carbamates
Source: Chem Sci. 2024 Nov 21;15(48):20534–44. doi: 10.1039/d4sc06814a (PMC11587147; doi:10.1039/d4sc06814a)
Supplement: SC-015-D4SC06814A-s001 [file SC-015-D4SC06814A-s001.pdf]

Supplementary Information (SI) for Chemical Science.
This journal is © The Royal Society of Chemistry 2024

Supplementary Information

Highly selective custom reduction products for hydrogenation of CO_2-derived urea derivatives and carbamates

Jun Zhu,[a] Yongtao Wang,[a] Jia Yao[a] and Haoran Li*[ab]
E-mail: lihr@zju.edu.cn

Table of Contents

1. General Experimental 4

2. Experimental procedures 5

2-1 Procedure for the catalytic hydrogenation of urea derivatives 5

2-2 Procedure for the catalytic hydrogenation of carbamates 6

3. Synthesis and Characterization of Iridium Complexes 8

3-1 $(PPh_3)_3Ir(CO)H$/bis((diphenylphosphanyl)methyl)pyridine under a N_2 atmosphere in a 1:1.5 molar ratio in THF 8

3-2 $(PPh_3)_3Ir(CO)H$/bis((diphenylphosphanyl)methyl)pyridine under a H_2 atmosphere in a 1:1.5 molar ratio in THF 9

3-3 $(PPh_3)_3Ir(CO)H$/bis((diphenylphosphanyl)methyl)pyridine/1,3-bis(4-chlorophenyl)urea under H_2 atmosphere in a 1:1.5:100 molar ratio in THF 11

3-4 $(PPh_3)_3Ir(CO)H$/bis((diphenylphosphanyl)methyl)pyridine/1,3-bis(4-chlorophenyl)urea under D_2 atmosphere in a 1:1.5:100 molar ratio in THF for 1 h 13

3-5 $(PPh_3)_3Ir(CO)H$/bis((diphenylphosphanyl)methyl)pyridine under a H_2 atmosphere in a 1:1.5 molar ratio in toluene 16

3-6 $(PPh_3)_3Ir(CO)H$/bis((diphenylphosphanyl)methyl)pyridine/1,3-bis(4-chlorophenyl)urea under D_2 atmosphere in a 1:1.5:100 molar ratio in toluene for 5 h 18

4. Reaction process for the catalytic hydrogenation of 1,3-diphenylurea 20

5. Catalytic hydrogenation of 1,3-diphenylurea in the presence of alcohol as co-solvent 22

5-1 Effect of methanol ratio in solvent for the catalytic hydrogenation of 1,3-diphenylurea to *N*-methylaniline and *N,N*-dimethylaniline 22

5-2 Effect of H_2 Pressure for the catalytic hydrogenation of 1,3-diphenylurea to *N*-methylaniline and *N,N*-dimethylaniline in the presence of methanol 24

5-3 Catalytic coupling of aniline and methanol in the H_2 atmosphere 25

5-4 Hydrogenation of 1,3-diphenylurea with CH_3OH in the absence of catalyst 26

5-5 Catalytic hydrogenation of 1,3-diphenylurea in the presence of ethanol as co-solvent 27

6. Catalytic hydrogenation of formamides, carbamates, or urea derivatives to methanol....... 28

7. Catalytic hydrogenation of formamides, carbamates, or urea derivatives to methylamines 29

8. Iridium-catalyzed transfer hydrogenation of urea derivatives to methylamines in the presence of alcohols 31

8-1 Conversion of urea derivatives to methylamines via transfer hydrogenation under N_2 atmosphere in the presence of methanol 31

8-2 Conversion of urea derivatives to methylamines under N_2 atmosphere in the presence of alcohol 32

9. Reaction process 33

9-1 Reaction process for the catalytic hydrogenation of 1,3-bis(4-chlorophenyl)urea 33

9-2 Reaction process for the catalytic hydrogenation of 1,3-bis(4-chlorophenyl)urea 38

9-3 Reaction process for the catalytic hydrogenation of 1,3-diphenylurea in the presence of methanol as cosolvent 39

10. Two-step process for conversion of urea derivatives and carbamates to methylamines under mild reaction conditions 44

11. Possible reaction pathways 45

11-1 Iridium-catalyzed hydrogenation of carbamates and urea derivatives to formamides 45

11-2 Iridium-catalyzed hydrogenation of carbamates and urea derivatives to methanol 46

11-3 Iridium-catalyzed hydrogenation of carbamates and urea derivatives to methylamines 47

12. Analysis of organic compounds 48

12-1 GC-MS Data of Products 48

12-2 NMR Data of Isolated Products 63

1. General Experimental

All experiments were carried out under an atmosphere of purified nitrogen in a Vacuum Atmospheres glove box. All solvents were reagent grade or better. Deuterated solvents were used as received. All solvents were degassed with argon and kept in the glove box over 4Å molecular sieves. All the chemicals used in the catalytic reactions (urea derivatives and carbamates) are commercially available.

^{1}H, ^{13}C and ^{31}P NMR spectra were recorded using Bruker-500 instrument. ^{1}H NMR chemical shifts are reported in ppm downfield from tetramethylsilane and referenced to the residual signals of an appropriate deuterated solvent. Mass spectra were recorded on Agilent 6545 Q-TOF, using Electro Spray Ionization (ESI) mode. GC-MS was carried out on a Shimadzu GC/MS-QP2010 system (Shimadzu, Germany), carried out using a DB-5 column (30 m × 0.25 mm × 0.25 μm film thickness), and helium as carrier gas. GC analysis were obtained on GC-2010 (Shimadzu, Japan) and carried out using a SH-5 column (30 m × 0.25 mm × 0.25 μm film thickness) using biphenyl as an internal standard. The quantitative analysis of the products (formamides, methanol, methylamines and amines) was performed by comparison with the corresponding authentic samples (commercially purchased). Inlets: 280 °C; Detector: FID 300 °C; Carrier Gas: N_2; Flow: 1 mL/min; Oven: 40 °C, hold 7 min; 5 °C/min to 80 °C, hold 3 min; 2 °C/min to 100 °C, hold 3 min; 20 °C/min to 300 °C, hold 3 min.

2. Experimental procedures

2-1 Procedure for the catalytic hydrogenation of urea derivatives

In a N_2 glove box, 0.02 mmol of the $(PPh_3)_3Ir(CO)H$ and 0.03 mmol of $Py(CH_2PPh_2)_2$ were added in 4 mL of THF to a 50 mL autoclave (Anhui CHEM^N Instrument Co., Ltd). This mixture was stirred for 5 min, then 2 mmol of urea derivative was added to it. Remove the sealed autoclave from the glove box, rinse it four times with hydrogen gas (Pressure to 50 bar, release to 2 bar, cycle four times) and pressurize it to the specified pressure, and heat it with stirring at the specified temperature. After the reaction, the steel autoclave was cooled in an ice-bath for 30 min and slowly depressurized. The biphenyl (internal standard) was added to the cold solution and then filtered through Celite, and the solution was analyzed by GC/GC-MS and ^1H NMR spectroscopy.

2-2 Procedure for the catalytic hydrogenation of carbamates

In a N_2 glove box, 0.06 mmol of the $(PPh_3)_3Ir(CO)H$ and 0.08 mmol of $Py(CH_2PPh_2)_2$ were added in 4 mL of THF to a 50 mL autoclave. This mixture was stirred for 5 min, then 1 mmol of carbamate was added to it. Remove the sealed autoclave from the glove box, rinse it four times with hydrogen gas (Pressure to 50 bar, release to 2 bar, cycle four times) and pressurize it to the specified pressure, and heat it with stirring at the specified temperature. After the reaction, the steel autoclave was cooled in an ice-bath for 30 min and slowly depressurized. The biphenyl (internal standard) was added to the cold solution and then filtered through Celite, and the solution was analyzed by GC/GC-MS and ^{1}H NMR spectroscopy.

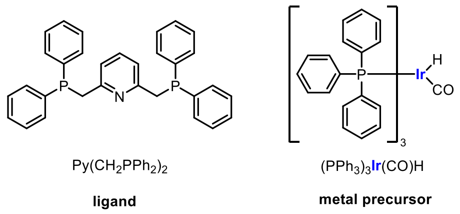

Figure S1. Catalyst system for the hydrogenation of urea derivatives and carbamates.

Table S1. Optimization of the reaction conditions for hydrogenation of methyl *N*-phenylcarbamate.[a]

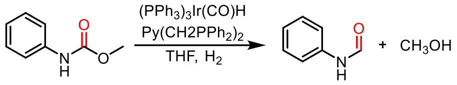

Entry	Ir/ligand (mol%/mol%)	T (°C)	P (bar)	t (h)	conv. (%)	yield (%) of amine	yield (%) of formamide	selec. (%)
1	1/1.5	140	30	24	44	1	42	95
2	2/3	140	30	24	60	3	56	93
3	3/4.5	140	30	24	70	6	63	90
4	4/6	140	30	24	73	8	65	89
5	3/4.5	140	30	48	78	15	63	81
6	2/3	150	30	24	80	12	68	85
7[b]	2/3	160	30	24	94	36	55	58
8[c]	2/3	180	30	24	99	71	10	10
9	3/4.5	150	30	24	88	23	65	81
10[d]	3/4.5	150	30	24	77	6	71	92
11[d]	6/8	150	30	24	90	11	78	87
12[d]	6/8	140	30	24	78	8	70	90
13[e]	6/8	140	30	24	90	6	84	93
14[e,f]	6/8	140	30	22	96	44	51	53
15[e]	6/8	140	10	24	88	8	80	91

[a]Reaction conditions: substrate (2 mmol), $(PPh_3)_3Ir(CO)H$ (3 mol%), $Py(CH_2PPh_2)_2$ (4.5 mol%), H_2 (30 bar), THF (4 mL), 140 °C (bath temperature). Determined by GC using biphenyl as an internal standard. Identification of the products was also confirmed by GC-MS. [b]Methylaniline (1%). [c]Methylaniline (18%). [d]THF (6 mL). [e]Substrate (1 mmol). [f]THF (3 mL).

3. Synthesis and Characterization of Iridium Complexes

3-1 $(PPh_3)_3Ir(CO)H$/bis((diphenylphosphanyl)methyl)pyridine under a N_2 atmosphere in a 1:1.5 molar ratio in THF

In a N_2 glove box, 0.02 mmol of the $(PPh_3)_3Ir(CO)H$ and 0.03 mmol of $Py(CH_2PPh_2)_2$ were added in 4 mL of THF to a 50 mL autoclave. Remove the sealed autoclave from the glove box, and heat it with stirring at 130 °C for 6 h. After the reaction, the steel autoclave was cooled in an ice-bath for 30 min and slowly depressurized. Afterwards, THF was slowly evaporated and the product precipitated form the concentrated reaction mixture which was analysed by NMR and ESI-MS.

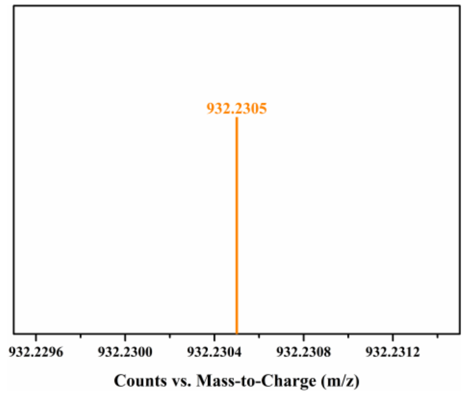

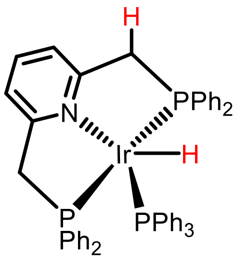

ESI-MS (CH_3OH) ($[C_{49}H_{43}IrNP_3]+H)^+$ (M+H) Calc.: 932.2310 Found: 932.2305 m/z.

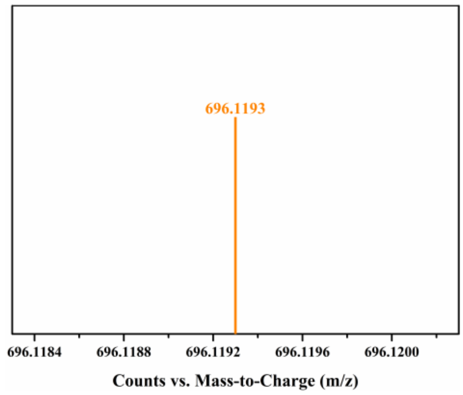

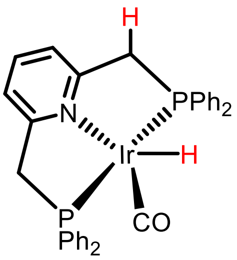

ESI-MS (CH_3OH) ($[C_{32}H_{28}IrNOP_2]-H)^+$ (M-H) Calc.: 696.1192 Found: 696.1193 m/z.

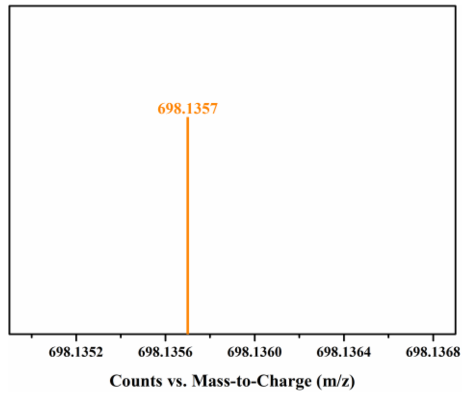

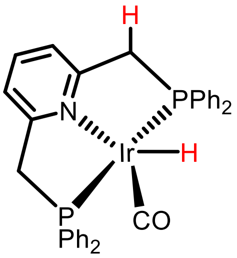

ESI-MS (CH_3OH) ($[C_{32}H_{28}IrNOP_2]+H)^+$ (M+H) Calc.: 698.1348 Found: 698.1357 m/z.

3-2 $(PPh_3)_3Ir(CO)H$/bis((diphenylphosphanyl)methyl)pyridine under a H_2 atmosphere in a 1:1.5 molar ratio in THF

In a N_2 glove box, 0.02 mmol of the $(PPh_3)_3Ir(CO)H$ and 0.03 mmol of $Py(CH_2PPh_2)_2$ were added in 4 mL of THF to a 50 mL autoclave. Remove the sealed autoclave from the glove box, rinse it four times with hydrogen gas (Pressure to 50 bar, release to 2 bar, cycle four times) and pressurize it to 10 bar, and heat it with stirring at 130 °C for 6 h. After the reaction, the steel autoclave was cooled in an ice-bath for 30 min and slowly depressurized. Afterwards, THF was slowly evaporated and the product precipitated form the concentrated reaction mixture which was analysed by NMR and ESI-MS.

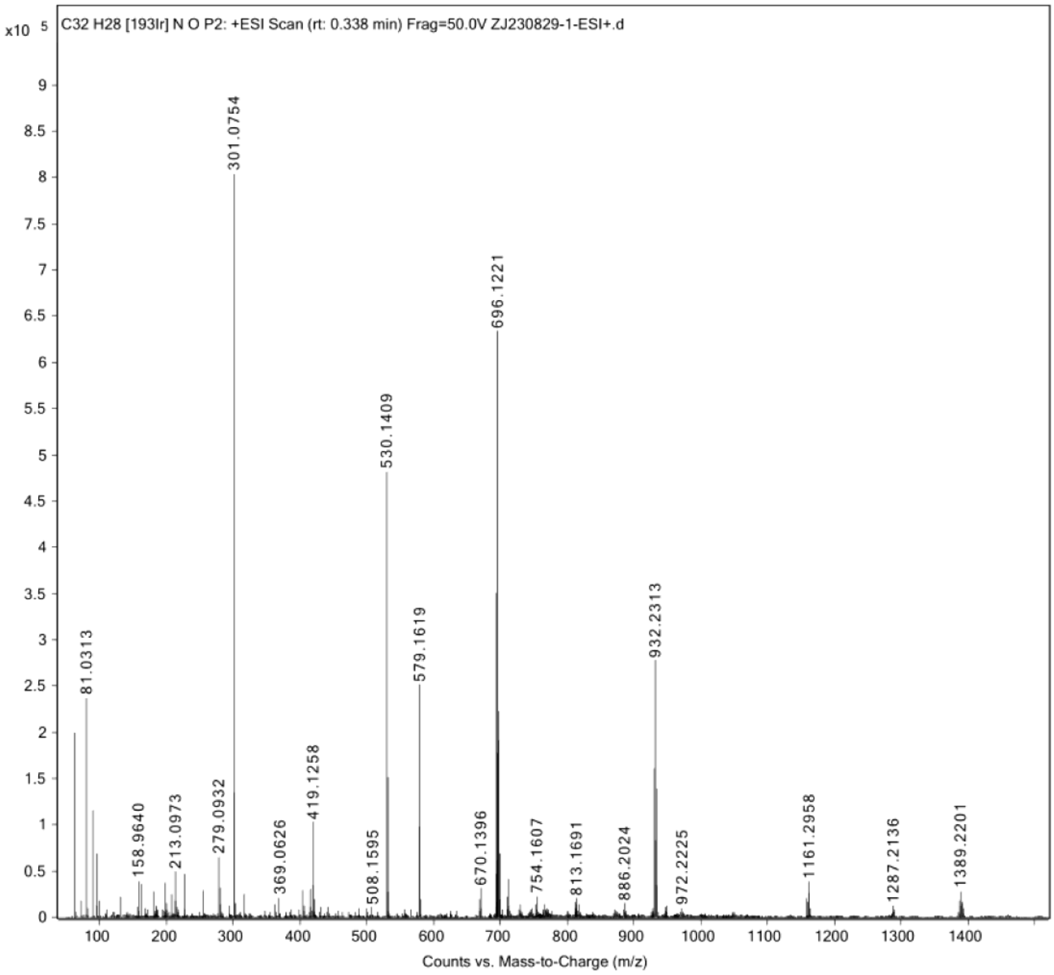

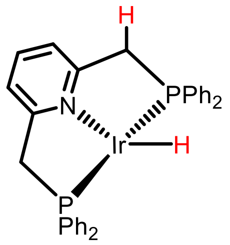

ESI-MS (CH$_3$OH) ([$C_{31}H_{28}IrNP_2$]+H)$^+$ (M+H) Calc.: 670.1399 Found: 670.1396 m/z.

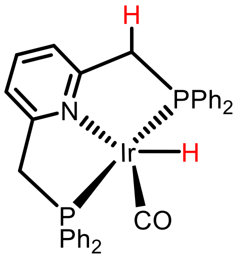

ESI-MS (CH$_3$OH) ([$C_{32}H_{28}IrNOP_2$]-H)$^+$ (M-H) Calc.: 696.1192 Found: 696.1221 m/z.

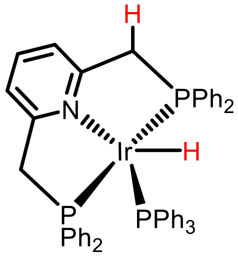

ESI-MS (CH$_3$OH) ([$C_{49}H_{43}IrNP_3$]+H)$^+$ (M+H) Calc.: 932.2310 Found: 932.2313 m/z.

3-3 $(PPh_3)_3Ir(CO)H$/bis((diphenylphosphanyl)methyl)pyridine/1,3-bis(4-chlorophenyl)urea under H_2 atmosphere in a 1:1.5:100 molar ratio in THF

In a N_2 glove box, 0.02 mmol of the $(PPh_3)_3Ir(CO)H$ and 0.03 mmol of $Py(CH_2PPh_2)_2$ were added in 4 mL of THF to a 50 mL autoclave. This mixture was stirred for 5 min, then 2 mmol of 1,3-bis(4-chlorophenyl)urea was added to it. Remove the sealed autoclave from the glove box, rinse it four times with hydrogen gas (Pressure to 50 bar, release to 2 bar, cycle four times) and pressurize it to 10 bar, and heat it with stirring at 130 °C for 45 min. After the reaction, the steel autoclave was cooled in an ice-bath for 30 min and slowly depressurized. Afterwards, THF was slowly evaporated and the product precipitated form the concentrated reaction mixture which was analysed by NMR and ESI-MS.

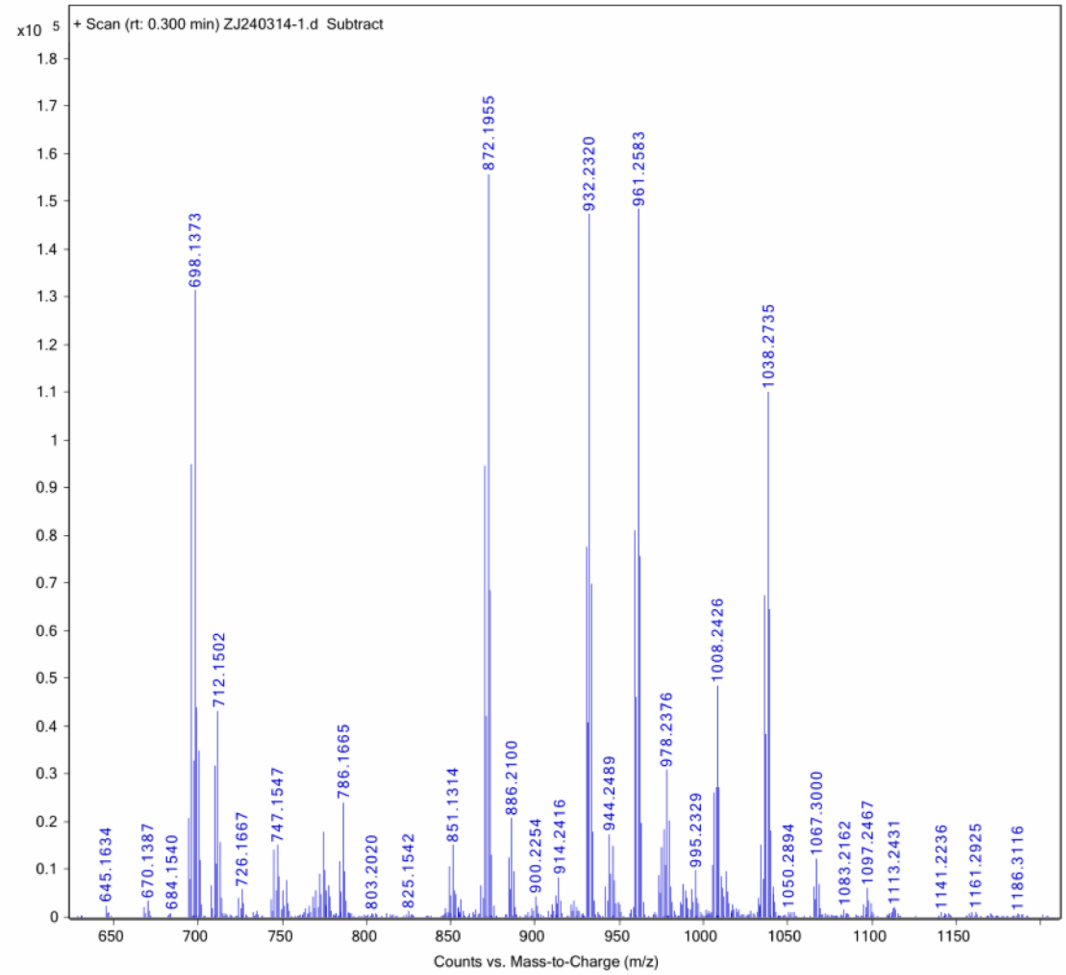

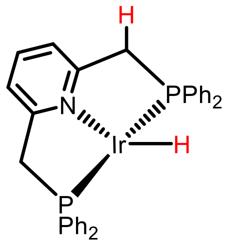

ESI-MS (CH_3OH) ($[C_{31}H_{28}IrNP_2]+H)^+$ (M+H) Calc.: 670.1399 Found: 670.1387 m/z.

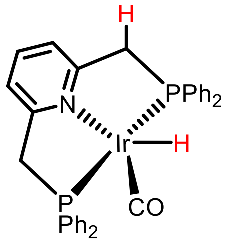

ESI-MS (CH_3OH) ($[C_{32}H_{28}IrNOP_2]+H)^+$ (M+H) Calc.: 698.1348 Found: 698.1373 m/z.

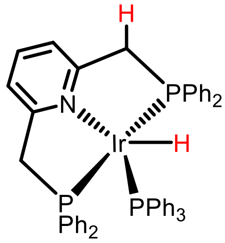

ESI-MS (CH_3OH) ($[C_{49}H_{43}IrNP_3]+H)^+$ (M+H) Calc.: 932.2310 Found: 932.2320 m/z.

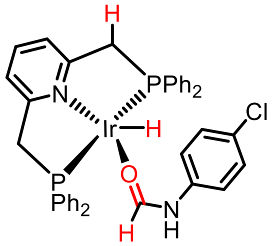

ESI-MS (CH_3OH) ($[C_{38}H_{34}ClIrN_2OP_2]+H)^+$ (M+H) Calc.: 825.1537 Found: 825.1542 m/z.

3-4 (PPh$_3$)$_3$Ir(CO)H/bis((diphenylphosphanyl)methyl)pyridine/1,3-bis(4-chlorophenyl)urea under D_2 atmosphere in a 1:1.5:100 molar ratio in THF for 1 h

In a N_2 glove box, 0.02 mmol of the (PPh$_3$)$_3$Ir(CO)H and 0.03 mmol of Py(CH$_2$PPh$_2$)$_2$ were added in 4 mL of THF to a 50 mL autoclave. This mixture was stirred for 5 min, then 2 mmol of urea derivative was added to it. Remove the sealed autoclave from the glove box, rinse it four times with D_2 gas (Pressure to 20 bar, release to 2 bar, cycle four times) and pressurize it to the 10 bar, and heat it with stirring at 130 °C for 1 h. After the reaction, the steel autoclave was cooled in an ice-bath for 30 min and slowly depressurized. Afterwards, THF was slowly evaporated and the product precipitated form the concentrated reaction mixture which was analysed by NMR and ESI-MS.

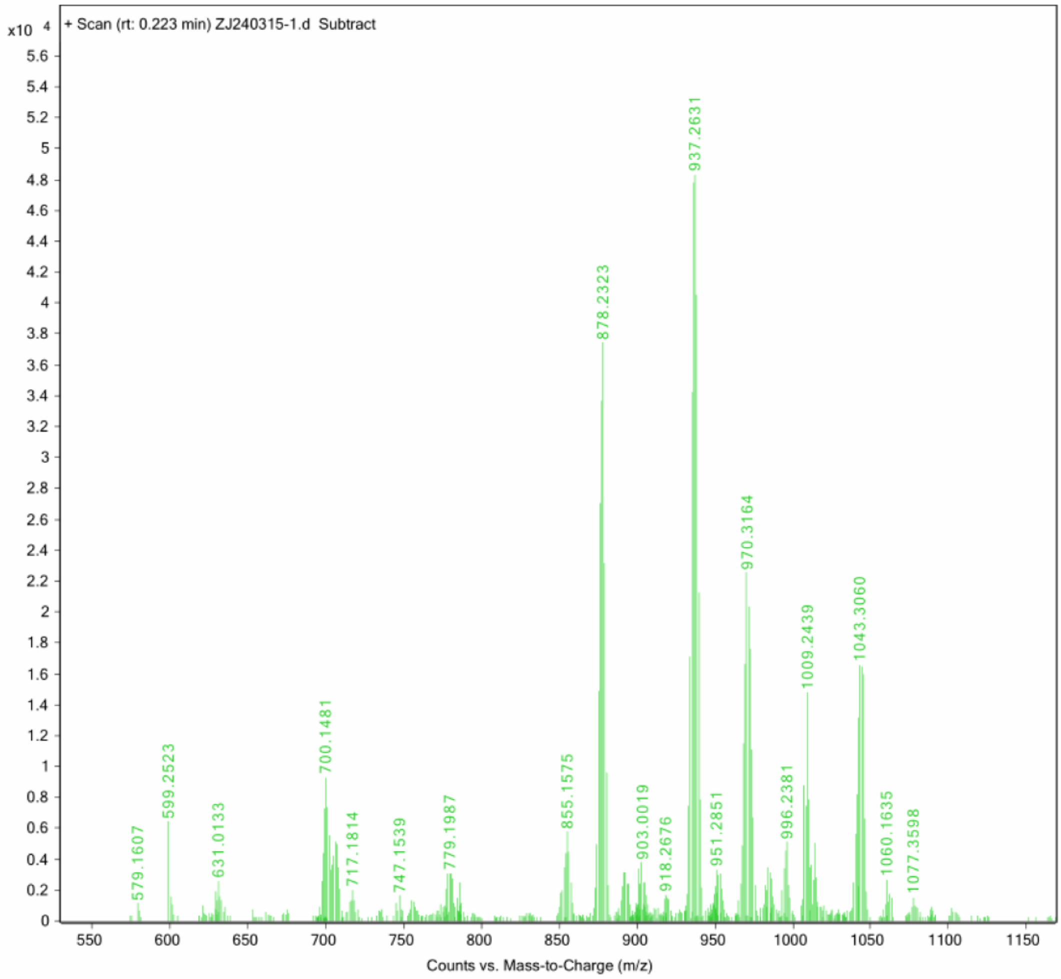

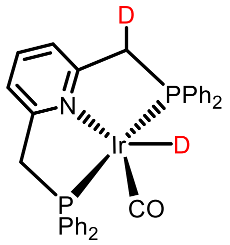

ESI-MS (CH_3OH) ($[C_{32}H_{26}D_2IrNOP_2]+H)^+$ (M+H) Calc.: 700.1474 Found: 700.1481 m/z.

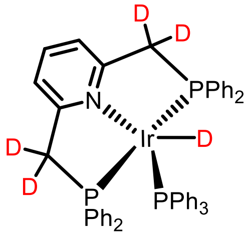

ESI-MS (CH_3OH) ($[C_{49}H_{38}D_5IrNP_3]+H)^+$ (M+H) Calc.: 937.2631 Found: 937.2624 m/z.

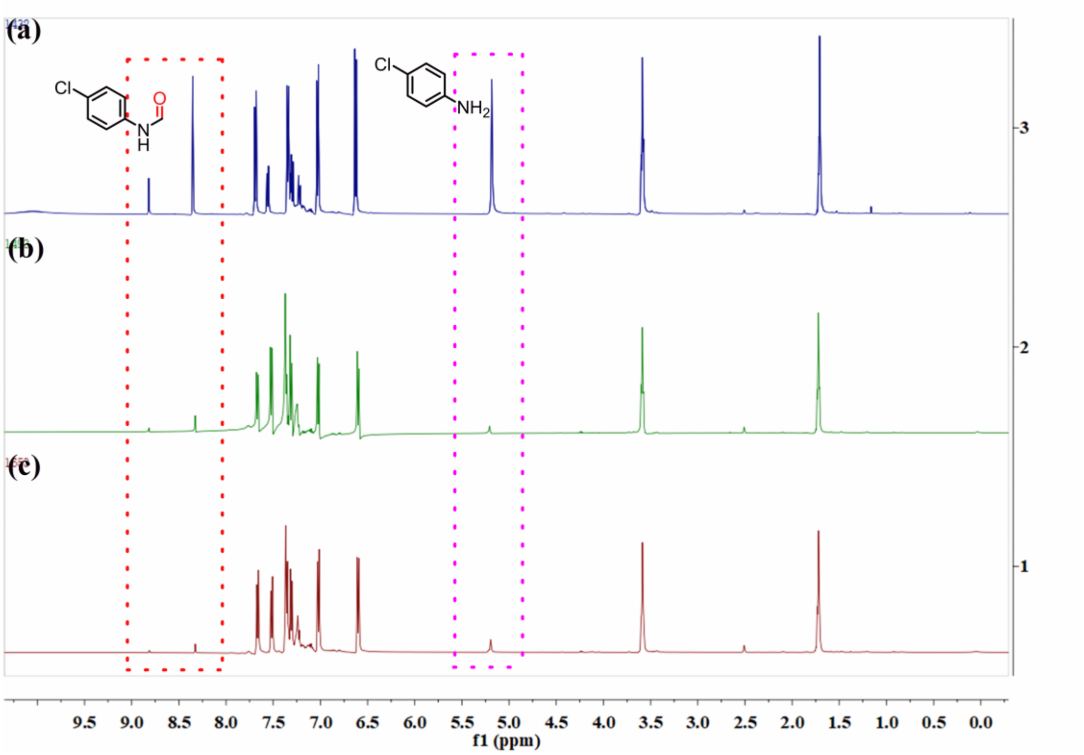

Figure S2. **(a)** ^1H NMR (500 MHz, DMSO-*d6*) spectra of $(PPh_3)_3Ir(CO)H$/bis((diphenylphosphanyl)methyl)pyridine/1,3-bis(4-chlorophenyl)urea under H_2 atmosphere in THF for 6 h, **(b)** $(PPh_3)_3Ir(CO)H$/bis((diphenylphosphanyl)methyl)pyridine/1,3-bis(4-chlorophenyl)urea under D_2 atmosphere in THF for 1 h, and **(c)** $(PPh_3)_3Ir(CO)H$/bis((diphenylphosphanyl)methyl)pyridine/1,3-bis(4-chlorophenyl)urea under D_2 atmosphere in THF for 6 h.

3-5 $(PPh_3)_3Ir(CO)H$/bis((diphenylphosphanyl)methyl)pyridine under a H_2 atmosphere in a 1:1.5 molar ratio in toluene

In a N_2 glove box, 0.02 mmol of the $(PPh_3)_3Ir(CO)H$ and 0.03 mmol of $Py(CH_2PPh_2)_2$ were added in 4 mL of toluene to a 50 mL autoclave. Remove the sealed autoclave from the glove box, rinse it four times with hydrogen gas (Pressure to 50 bar, release to 2 bar, cycle four times) and pressurize it to 10 bar, and heat it with stirring at 130 °C for 6 h. After the reaction, the steel autoclave was cooled in an ice-bath for 30 min and slowly depressurized. Afterwards, toluene was slowly evaporated and the product precipitated form the concentrated reaction mixture which was analysed by NMR and ESI-MS.

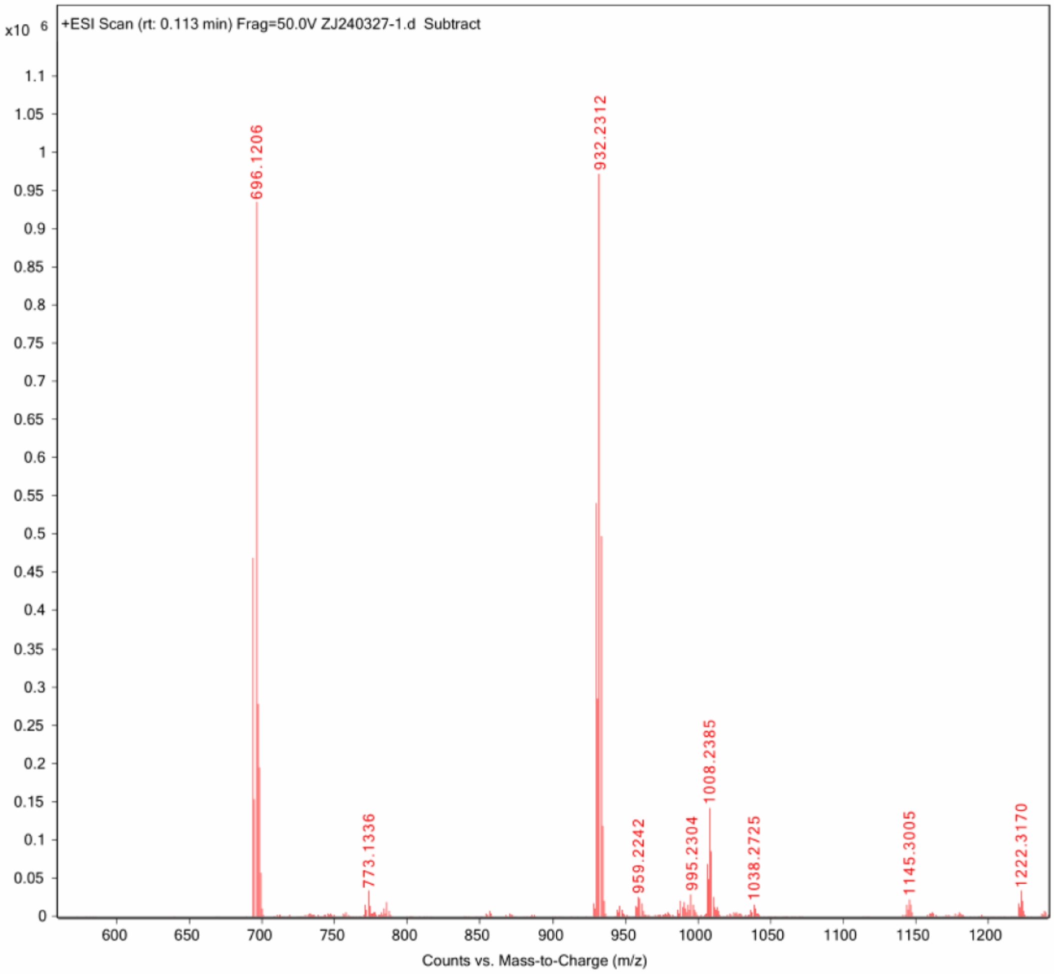

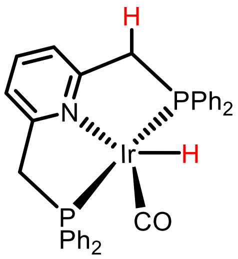

ESI-MS (CH_3OH) ($[C_{32}H_{28}IrNOP_2]$-H)$^+$ (M-H) Calc.: 696.1192 Found: 696.1206 m/z.

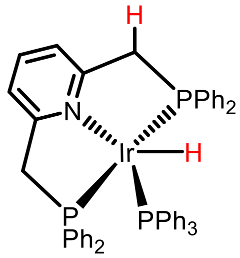

ESI-MS (CH$_3$OH) ([$C_{49}H_{43}IrNP_3$]+H)$^+$ (M+H) Calc.: 932.2310 Found: 932.2312 m/z.

3-6 $(PPh_3)_3Ir(CO)H$/bis((diphenylphosphanyl)methyl)pyridine/1,3-bis(4-chlorophenyl)urea under D_2 atmosphere in a 1:1.5:100 molar ratio in toluene for 5 h

In a N_2 glove box, 0.02 mmol of the $(PPh_3)_3Ir(CO)H$ and 0.03 mmol of $Py(CH_2PPh_2)_2$ were added in 4 mL of toluene to a 50 mL autoclave. This mixture was stirred for 5 min, then 2 mmol of urea derivative was added to it. Remove the sealed autoclave from the glove box, rinse it four times with D_2 gas (Pressure to 10 bar, release to 2 bar, cycle four times) and pressurize it to the 10 bar, and heat it with stirring at 150 °C for 5 h. After the reaction, the steel autoclave was cooled in an ice-bath for 30 min and slowly depressurized. Afterwards, toluene was slowly evaporated and the product precipitated form the concentrated reaction mixture which was analysed by NMR and ESI-MS.

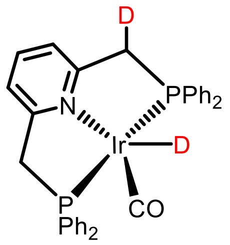

ESI-MS (CH_3OH) $([C_{32}H_{26}D_2IrNOP_2]+H)^+$ (M+H) Calc.: 700.1474 Found: 700.1469 m/z.

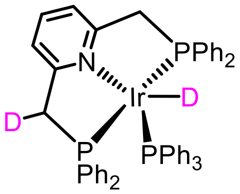

ESI-MS (CH_3OH) $([C_{49}H_{38}D_5IrNP_3]+H)^+$ (M+H) Calc.: 934.2436 Found: 934.244 m/z.

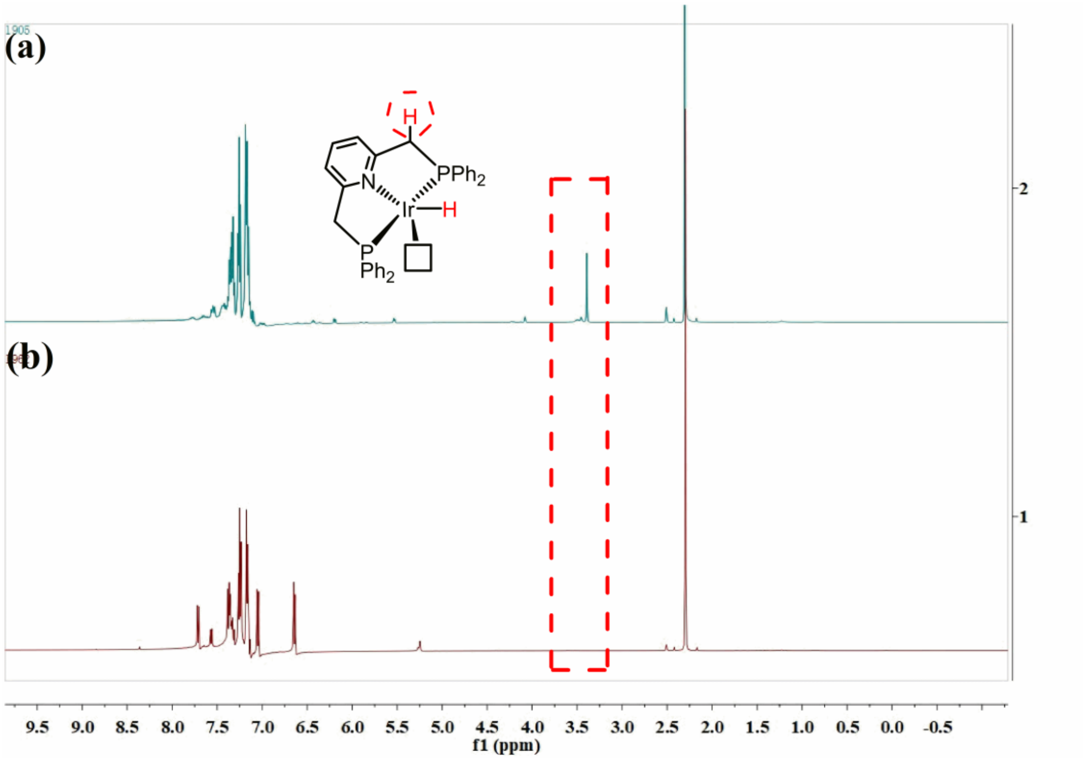

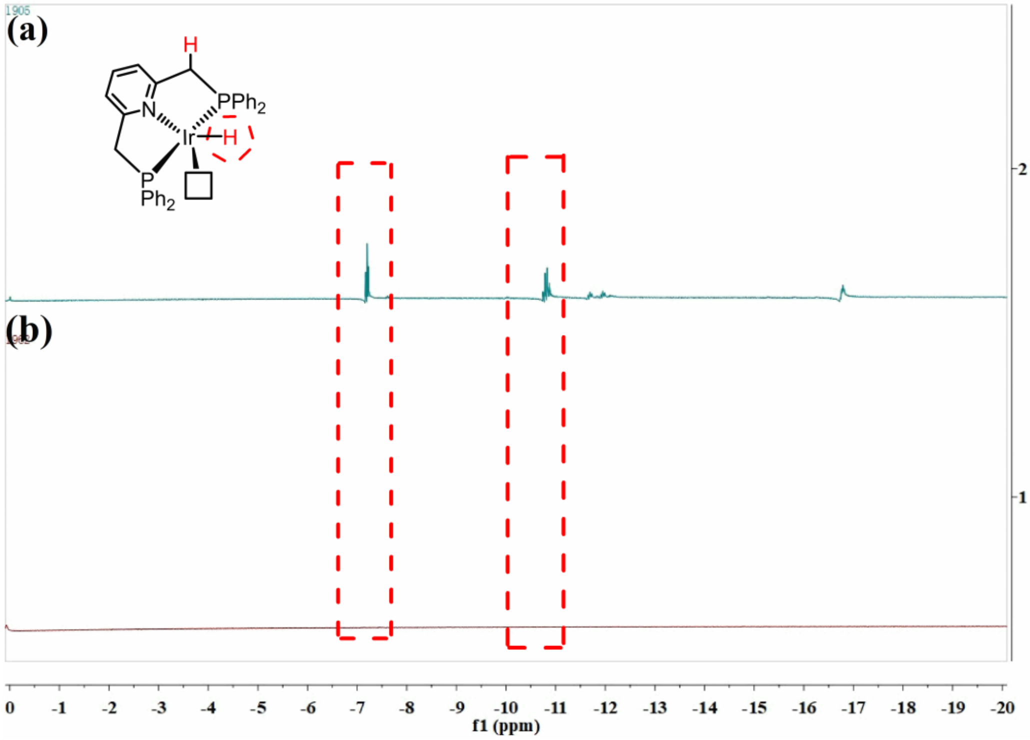

Figure S3. **(a)** ^{1}H NMR (500 MHz, DMSO-*d6*) spectra of $(PPh_3)_3Ir(CO)H$/bis((diphenylphosphanyl)methyl)pyridine under D_2 atmosphere in toluene, **(b)** $(PPh_3)_3Ir(CO)H$/bis((diphenylphosphanyl)methyl)pyridine/1,3-bis(4-chlorophenyl)urea under D_2 atmosphere in toluene.

4. Reaction process for the catalytic hydrogenation of 1,3-diphenylurea

In a N_2 glove box, 0.04 mmol of the $(PPh_3)_3Ir(CO)H$ and 0.06 mmol of $Py(CH_2PPh_2)_2$ were added in 4 mL of THF to a 50 mL autoclave. This mixture was stirred for 5 min, then 2 mmol 1,3-diphenylurea was added to it. Remove the sealed autoclave from the glove box, rinse it four times with hydrogen gas (Pressure to 50 bar, release to 2 bar, cycle four times) and pressurize it to the 30 bar, and heat it with stirring at the specified temperature. After the reaction, the steel autoclave was cooled in an ice-bath for 30 min and slowly depressurized. The biphenyl (internal standard) was added to the cold solution and then filtered through Celite, and the solution was analyzed by GC/GC-MS.

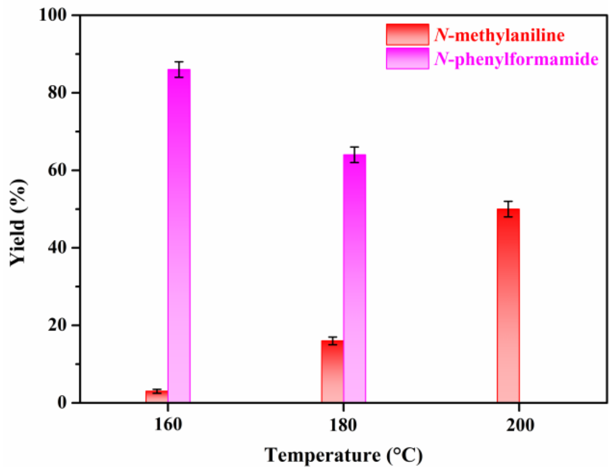

Figure S4. Hydrogenation of 1,3-diphenylurea at different reaction temperature. Reaction conditions: 1,3-diphenylurea (2 mmol), $(PPh_3)_3Ir(CO)H$ (2 mol%), $Py(CH_2PPh_2)_2$ (3 mol%), 30 bar H_2, THF (4 mL), reaction time (18 h).

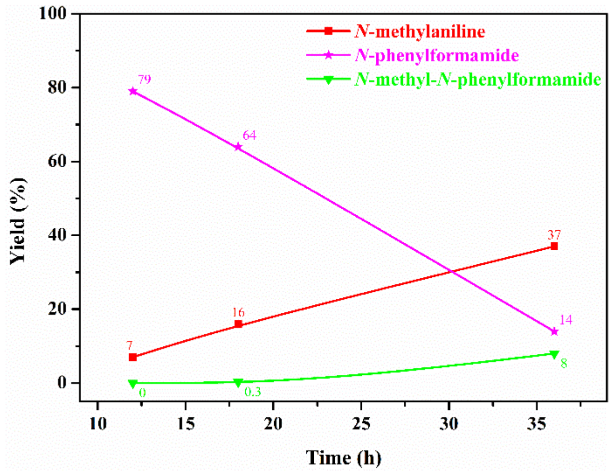

Figure S5. Reaction profile for the hydrogenation of 1,3-diphenylurea. Reaction conditions: 1,3-diphenylurea (2 mmol), $(PPh_3)_3Ir(CO)H$ (2 mol%), $Py(CH_2PPh_2)_2$ (3 mol%), 30 bar H_2 (separate trials at varying reaction time), THF (4 mL), reaction temperature (180 °C).

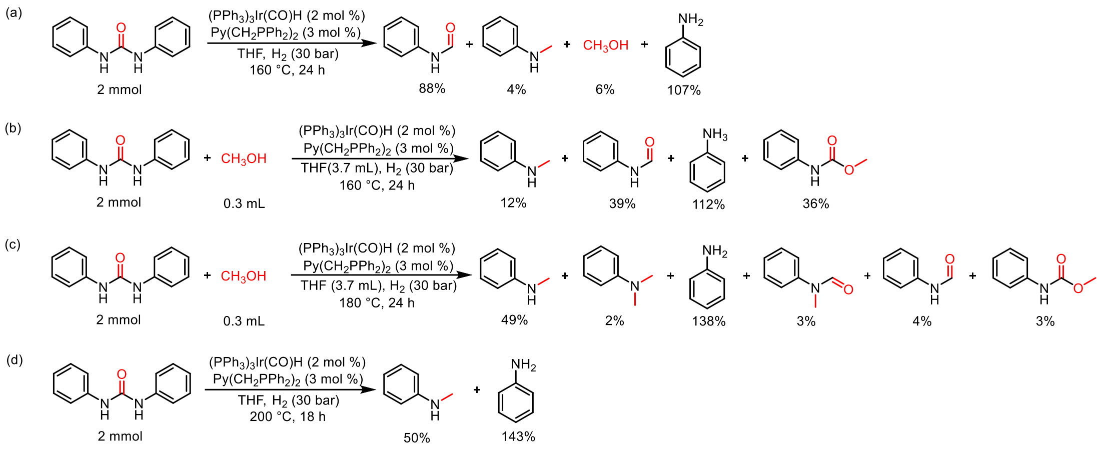

Figure S6. Catalytic hydrogenation of 1,3-diphenylurea in the absence/presence of CH_3OH.

5. Catalytic hydrogenation of 1,3-diphenylurea in the presence of alcohol as co-solvent

5-1 Effect of methanol ratio in solvent for the catalytic hydrogenation of 1,3-diphenylurea to *N*-methylaniline and *N,N*-dimethylaniline

In a N_2 glove box, 0.04 mmol of the $(PPh_3)_3Ir(CO)H$ and 0.06 mmol of $Py(CH_2PPh_2)_2$ were added in 4 mL of solvent (methanol and THF as co-solvent) to a 50 mL autoclave. This mixture was stirred for 5 min, then 2 mmol 1,3-diphenylurea was added to it. Remove the sealed autoclave from the glove box, rinse it four times with hydrogen gas (Pressure to 50 bar, release to 2 bar, cycle four times) and pressurize it to the 30 bar, and heat it with stirring at the specified temperature. After the reaction, the steel autoclave was cooled in an ice-bath for 30 min and slowly depressurized. The biphenyl (internal standard) was added to the cold solution and then filtered through Celite, and the solution was analyzed by GC/GC-MS.

a)

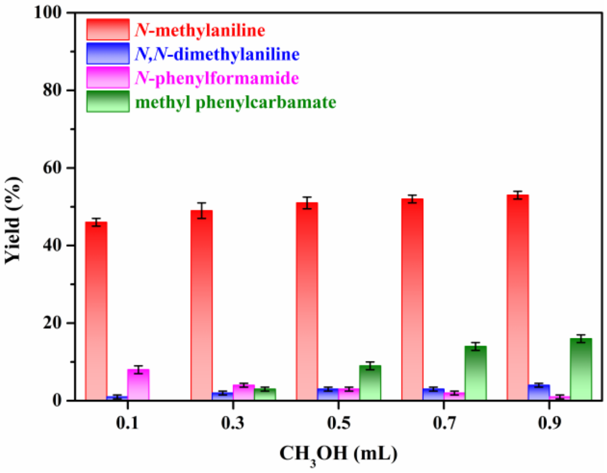

b)

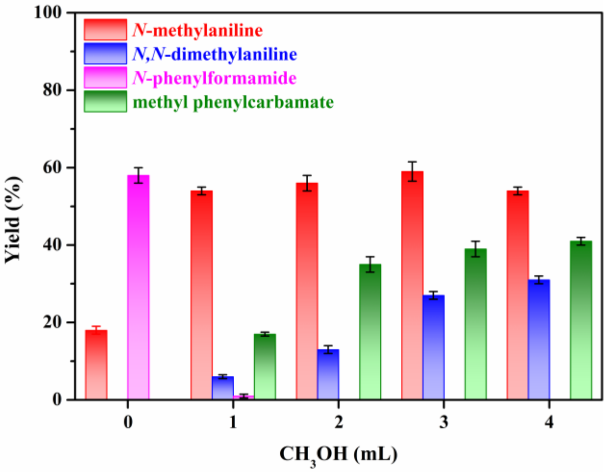

c)

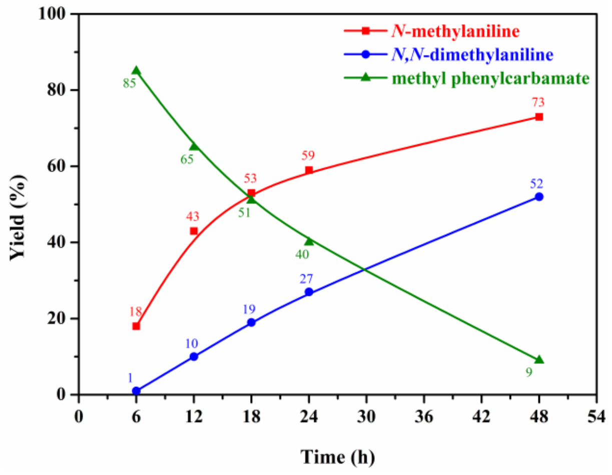

Figure S7. Hydrogenation of 1,3-diphenylurea in the presence of CH_3OH as co-solvent. (a) and (b) Reaction conditions: 1,3-diphenylurea (2 mmol), $(PPh_3)_3Ir(CO)H$ (2 mol%), $Py(CH_2PPh_2)_2$ (3 mol%), 30 bar H_2 (separate trials at varying reaction time), solvent (THF and CH_3OH; 4 mL), reaction time (24 h). (c) Reaction conditions: 1,3-diphenylurea (2 mmol), $(PPh_3)_3Ir(CO)H$ (2 mol%), $Py(CH_2PPh_2)_2$ (3 mol%), H_2 (30 bar), solvent (3.0 mL methanol and 1.0 mL THF), reaction temperature (180 °C), reaction times (6-48 h).

5-2 Effect of H_2 Pressure for the catalytic hydrogenation of 1,3-diphenylurea to *N*-methylaniline and *N,N*-dimethylaniline in the presence of methanol

In a N_2 glove box, 0.04 mmol of the $(PPh_3)_3Ir(CO)H$ and 0.06 mmol of $Py(CH_2PPh_2)_2$ were added in 4 mL of solvent (0.3 mL methanol and 3.7 mL THF) to a 50 mL autoclave. This mixture was stirred for 5 min, then 2 mmol 1,3-diphenylurea was added to it. Remove the sealed autoclave from the glove box, rinse it four times with hydrogen gas (Pressure to 50 bar, release to 2 bar, cycle four times) and pressurize it to the desired pressure, and heat it with stirring at 180 °C. After the reaction, the steel autoclave was cooled in an ice-bath for 30 min and slowly depressurized. The biphenyl (internal standard) was added to the cold solution and then filtered through Celite, and the solution was analyzed by GC/GC-MS.

i)

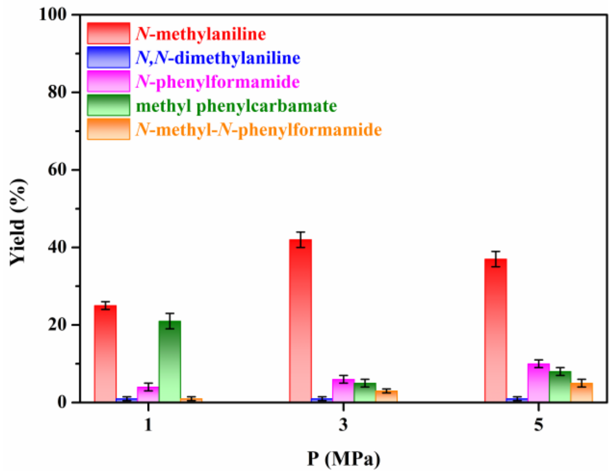

ii)

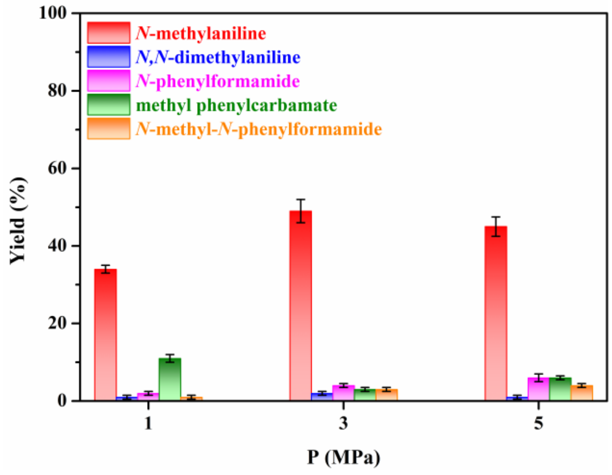

Figure S8. Hydrogenation of 1,3-diphenylurea in the presence of CH_3OH as co-solvent. Reaction conditions: 1,3-diphenylurea (2 mmol), $(PPh_3)_3Ir(CO)H$ (2 mol%), $Py(CH_2PPh_2)_2$ (3 mol%), reaction temperature (180 °C), solvent (3.7 mL THF and 0.3 mL CH_3OH). **i**, Reaction time (18 h). **ii**, Reaction time (24 h).

5-3 Catalytic coupling of aniline and methanol in the H_2 atmosphere

In a N_2 glove box, 0.04 mmol of the $(PPh_3)_3Ir(CO)H$ and 0.06 mmol of $Py(CH_2PPh_2)_2$ were added in 4 mL of solvent (0.3 mL methanol and 3.7 mL THF) to a 50 mL autoclave. This mixture was stirred for 5 min, then 4 mmol of aniline was added to it. Remove the sealed autoclave from the glove box, rinse it four times with hydrogen gas (Pressure to 50 bar, release to 2 bar, cycle four times) and pressurize it to 30 bar, and heat it with stirring at the desired temperature. After the reaction, the steel autoclave was cooled in an ice-bath for 30 min and slowly depressurized. The biphenyl (internal standard) was added to the cold solution and then filtered through Celite, and the solution was analyzed by GC/GC-MS.

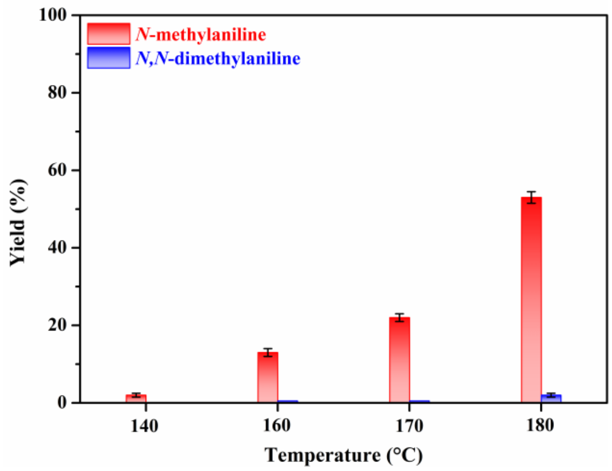

Figure S9. Catalytic coupling of aniline and methanol in the H_2 atmosphere. Reaction conditions: aniline (4 mmol), $(PPh_3)_3Ir(CO)H$ (2 mol%), $Py(CH_2PPh_2)_2$ (3 mol%), 30 bar H_2 (separate trials at varying reaction time), solvent (0.3 mL methanol and 3.7 mL THF), reaction time (24 h), reaction temperature (140-180 °C).

5-4 Hydrogenation of 1,3-diphenylurea with CH_3OH in the absence of catalyst

2 mmol of 1,3-diphenylurea was added in 4 mL of solvent (0.1 mL methanol and 3.9 mL THF) to a 50 mL autoclave. This mixture was stirred for 5 min, then 2 mmol 1,3-diphenylurea was added to it. The autoclave was then sealed, rinsed it with nitrogen and hydrogen four times each (pressure to 50 bar, release to 2 bar), and pressurized to the desired pressure, and heat it with stirring at 140 °C. After the reaction, the steel autoclave was cooled in an ice-bath for 30 min and slowly depressurized. The biphenyl (internal standard) was added to the cold solution and then filtered through Celite, and the solution was analyzed by GC.

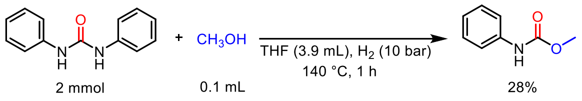

Figure S10. Hydrogenation of 1,3-diphenylurea without catalyst in the presence of CH_3OH.

5-5 Catalytic hydrogenation of 1,3-diphenylurea in the presence of ethanol as co-solvent

In a N_2 glove box, 0.04 mmol of the $(PPh_3)_3Ir(CO)H$ and 0.06 mmol of $Py(CH_2PPh_2)_2$ were added in 4 mL of solvent (0.3 mL ethanol and 3.7 mL THF) to a 50 mL autoclave. This mixture was stirred for 5 min, then 2 mmol of 1,3-diphenylurea was added to it. Remove the sealed autoclave from the glove box, rinse it four times with hydrogen gas (Pressure to 50 bar, release to 2 bar, cycle four times) and pressurize it to the 30 bar, and heat it with stirring at the desired reaction temperature. After the reaction, the steel autoclave was cooled in an ice-bath for 30 min and slowly depressurized. The biphenyl (internal standard) was added to the cold solution and then filtered through Celite, and the solution was analyzed by GC/GC-MS.

(a)

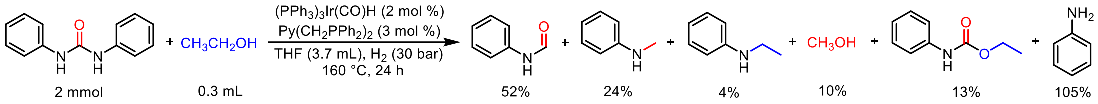

(b)

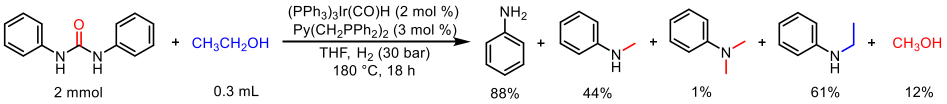

Figure S11. Hydrogenation of 1,3-diphenylurea in the presence of CH_3CH_2OH as co-solvent. **a,** Reaction temperature (160 °C); **b,** Reaction temperature (180 °C).

6. Catalytic hydrogenation of formamides, carbamates, or urea derivatives to methanol

In a N_2 glove box, $(PPh_3)_3Ir(CO)H$ and $Py(CH_2PPh_2)_2$ were added in 4 mL of THF to a 50 mL autoclave. This mixture was stirred for 5 min, then 1-2 mmol of substrate was added to it. Remove the sealed autoclave from the glove box, rinse it four times with hydrogen gas (Pressure to 50 bar, release to 2 bar, cycle four times) and pressurize it to the 30 bar, and heat it with stirring at the desired reaction temperature. After the reaction, the steel autoclave was cooled in an ice-bath for 30 min and slowly depressurized. The biphenyl (internal standard) was added to the cold solution and then filtered through Celite, and the solution was analyzed by GC/GC-MS.

Table S2. Iridium-catalyzed hydrogenation of formamides, carbamates and urea derivatives to methanol[a]

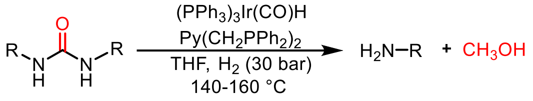

entry	substrate	time (h)	yield (%) of methanol[b]	yield (%) of *N*-methylamines[b]	conversion (%)
**1**	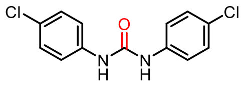	50[c]	84	8	> 99
**2**	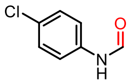	48	51	11	75
**3**	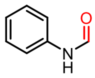	24[d]	40	3	54
**4**	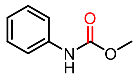	24[d,e]	73	6	99

[a]Reaction conditions: substrate (2 mmol), $(PPh_3)_3Ir(CO)H$ (2 mol%), $Py(CH_2PPh_2)_2$ (3 mol%), H_2 (30 bar), THF (4 mL), 150 °C (bath temperature). [b]Yield determined by GC using biphenyl as an internal standard. Identification of the products were also confirmed by GC-MS and ^1H NMR. [c]$(PPh_3)_3Ir(CO)H$ (4 mol%), $Py(CH_2PPh_2)_2$ (5 mol%) were used. [d]160 °C (bath temperature). [e]substrate (1 mmol), $(PPh_3)_3Ir(CO)H$ (6 mol%), $Py(CH_2PPh_2)_2$ (8 mol%), THF (3 mL).

7. Catalytic hydrogenation of formamides, carbamates, or urea derivatives to methylamines

In a N_2 glove box, $(PPh_3)_3Ir(CO)H$ and $Py(CH_2PPh_2)_2$ were added in 4 mL of solvent (ethanol and THF) to a 50 mL autoclave. This mixture was stirred for 5 min, then 2 mmol of substrate was added to it. Remove the sealed autoclave from the glove box, rinse it four times with hydrogen gas (Pressure to 50 bar, release to 2 bar, cycle four times) and pressurize it to the 30 bar, and heat it with stirring at 180 °C. After the reaction, the steel autoclave was cooled in an ice-bath for 30 min and slowly depressurized. The biphenyl (internal standard) was added to the cold solution and then filtered through Celite, and the solution was analyzed by GC/GC-MS.

Table S3. Iridium-catalyzed hydrogenation of urea derivatives to methylamines[a]

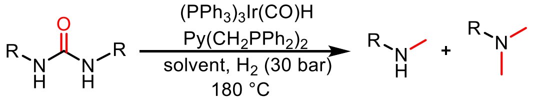

entry	substrate	time (h)	yield (%) of *N*-methylamines	yield (%) of *N,N*-dimethylamines
**1**	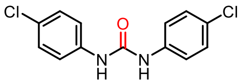	22	27	2
		48[b]	52	7
**2**	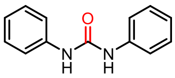	48[c]	66	7
**3**	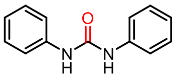	36[d,g]	97	30
**4**	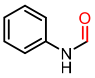	45[e,f]	54	38
**5**	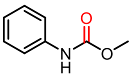	36[g]	46	6
**6**	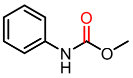	48[e,g]	49	39
**7**	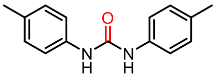	48[g]	51	10
**8**	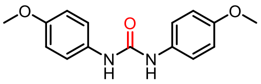	48	87	61

[a]Reaction conditions: substrate (2 mmol), $(PPh_3)_3Ir(CO)H$ (2 mol%), $Py(CH_2PPh_2)_2$ (3 mol%), H_2 (30 bar), solvent (2.0 mL MeOH and 2.0 mL THF), 180 °C (bath temperature). Yield determined by GC using biphenyl as an internal standard. Identification of the products were also confirmed by GC-MS and ^1H NMR. [b]$(PPh_3)_3Ir(CO)H$ (4 mol%), $Py(CH_2PPh_2)_2$ (5 mol%). [c]Solvent (0.3 mL MeOH and 3.7 mL THF). [d]$(PPh_3)_3Ir(CO)H$ (4 mol%), $Py(CH_2PPh_2)_2$ (5 mol%). [e]$(PPh_3)_3Ir(CO)H$ (3 mol%), $Py(CH_2PPh_2)_2$ (4 mol%). [f]Solvent (1.0 mL MeOH and 3.0 mL THF). [g]Solvent (0.5 mL MeOH and 3.5 mL THF).

8. Iridium-catalyzed transfer hydrogenation of urea derivatives to methylamines in the presence of alcohols

8-1 Conversion of urea derivatives to methylamines via transfer hydrogenation under N_2 atmosphere in the presence of methanol

In a N_2 glove box, 0.04 mmol of the $(PPh_3)_3Ir(CO)H$ and 0.06 mmol of $Py(CH_2PPh_2)_2$ were added in 4 mL of solvent (0.5 mL ethanol and 3.5 mL THF) to a 50 mL autoclave. This mixture was stirred for 5 min, then 2 mmol of 1,3-diphenylurea was added to it. Remove the sealed autoclave from the glove box, rinse it four times with nitrogen gas (Pressure to 30 bar, release to 2 bar, cycle four times) and pressurize it to the 10 bar, and heat it with stirring at 160 °C. After the reaction, the steel autoclave was cooled in an ice-bath for 30 min and slowly depressurized. The biphenyl (internal standard) was added to the cold solution and then filtered through Celite, and the solution was analyzed by GC/GC-MS.

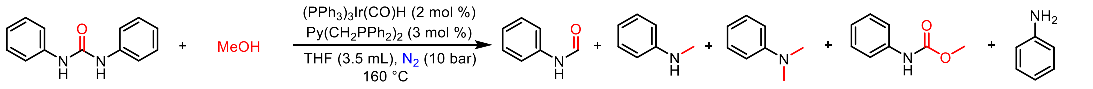

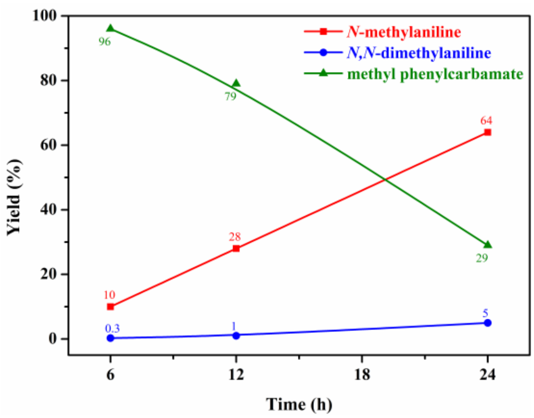

Figure S12. Iridium-catalyzed transfer hydrogenation of urea derivatives to methylamines. Reaction conditions: 1,3-diphenylurea (2 mmol), $(PPh_3)_3Ir(CO)H$ (2 mol%), $Py(CH_2PPh_2)_2$ (3 mol%), N_2 atmosphere, solvent (0.5 mL methanol and 3.5 mL THF), reaction temperature (160 °C), reaction times (6-24 h).

8-2 Conversion of urea derivatives to methylamines under N_2 atmosphere in the presence of alcohol

In a N_2 glove box, 0.04 mmol of the $(PPh_3)_3Ir(CO)H$ and 0.06 mmol of $Py(CH_2PPh_2)_2$ were added in 4 mL of solvent (0.5 mL alcohol and 3.5 mL THF) to a 50 mL autoclave. This mixture was stirred for 5 min, then 2 mmol of 1,3-diphenylurea was added to it. Remove the sealed autoclave from the glove box, rinse it four times with nitrogen gas (Pressure to 30 bar, release to 2 bar, cycle four times) and pressurize it to the 10 bar, and heat it with stirring at the desired reaction temperature. After the reaction, the steel autoclave was cooled in an ice-bath for 30 min and slowly depressurized. The biphenyl (internal standard) was added to the cold solution and then filtered through Celite, and the solution was analyzed by GC/GC-MS.

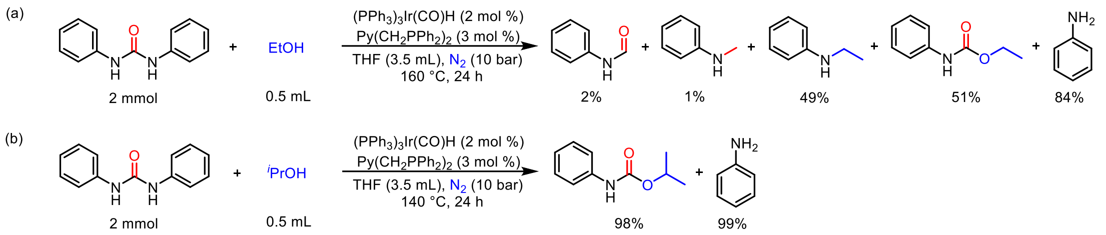

Figure S13. Conversion of 1,3-diphenylurea and alcohol. a, Conversion of 1,3-diphenylurea in the presence of EtOH. **b,** Conversion of 1,3-diphenylurea in the presence of ^{i}PrOH.

9. Reaction process

9-1 Reaction process for the catalytic hydrogenation of 1,3-bis(4-chlorophenyl)urea

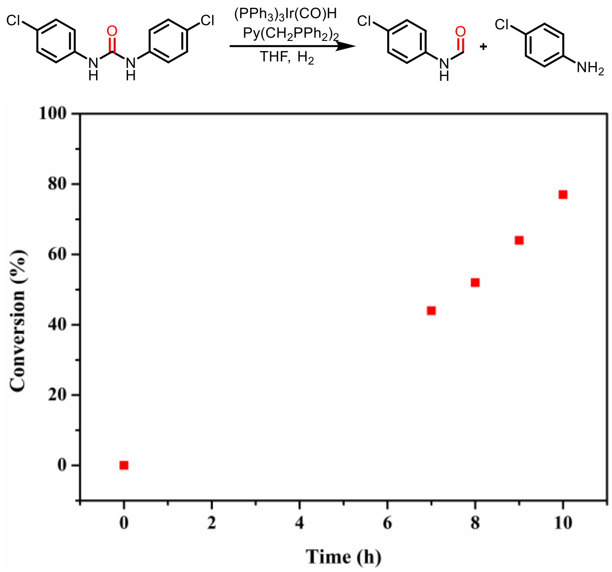

Figure S14. Reaction conditions: 1,3-bis(4-chlorophenyl)urea) (2 mmol), $(PPh_3)_3Ir(CO)H$ (0.005 mmol), $Py(CH_2PPh_2)_2$ (0.0075 mmol), H_2 (10 bar), 130 °C, THF (4 mL).

(a)

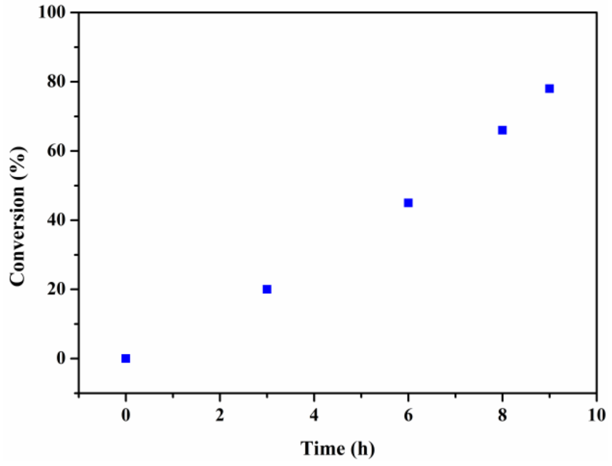

Figure S15. Reaction conditions: 1,3-bis(4-chlorophenyl)urea) (2 mmol), $(PPh_3)_3Ir(CO)H$ (0.01 mmol), $Py(CH_2PPh_2)_2$ (0.015 mmol), H_2 (10 bar), 130 °C, THF (4 mL).

(b)

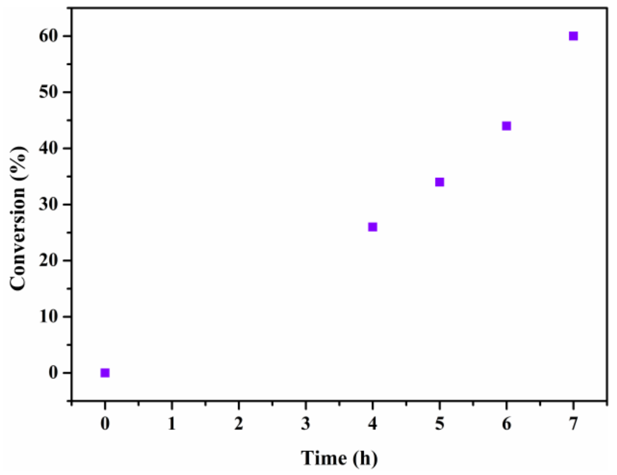

Figure S16. Reaction conditions: 1,3-bis(4-chlorophenyl)urea (2 mmol), $(PPh_3)_3Ir(CO)H$ (0.005 mmol), $Py(CH_2PPh_2)_2$ (0.0075 mmol), H_2 (30 bar), 130 °C, THF (4 mL).

A longer time-course profile for semi-hydrogenation of 1,3-bis(4-chlorophenyl)urea:

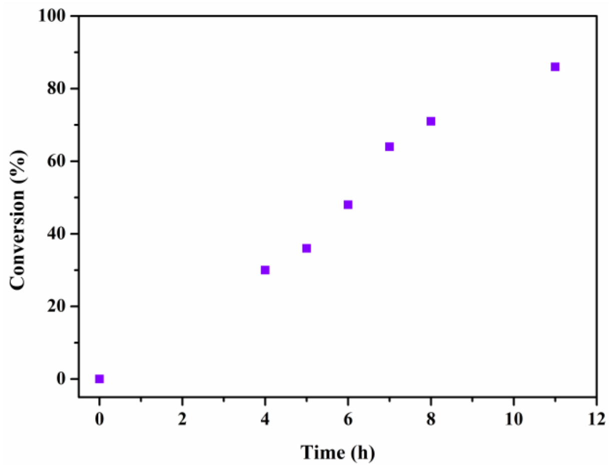

In the early stage of the semi-hydrogenation reactions, catalytic active substances are gradually formed, so there is an induction period observed in the presented time profile. In the later stage of the reaction, the reaction rate would gradually decrease.

(c)

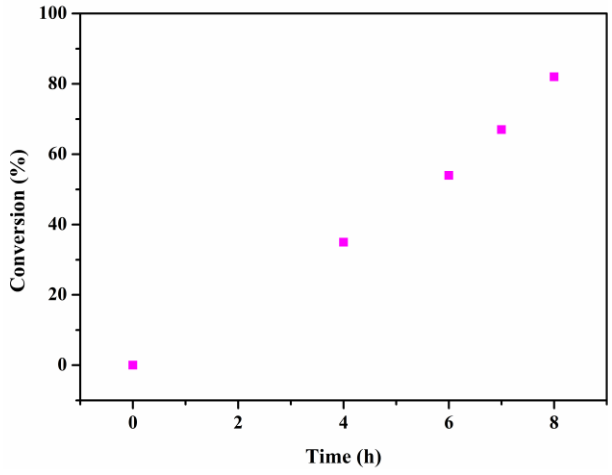

Figure S17. Reaction conditions: 1,3-bis(4-chlorophenyl)urea) (1.5 mmol), $(PPh_3)_3Ir(CO)H$ (0.005 mmol), $Py(CH_2PPh_2)_2$ (0.0075 mmol), H_2 (10 bar), 130 °C, THF (4 mL).

(d)

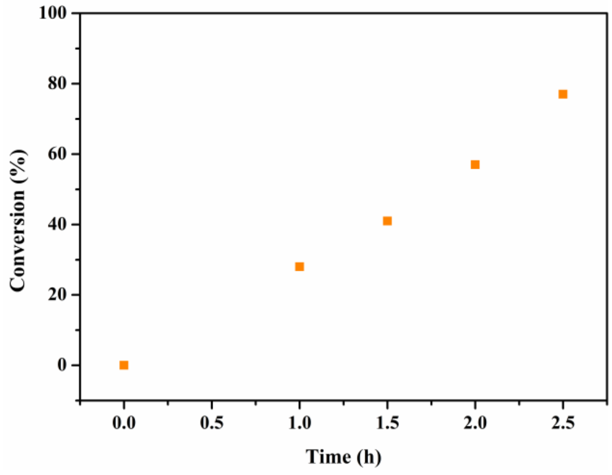

Figure S18. Reaction conditions: 1,3-bis(4-chlorophenyl)urea) (2 mmol), $(PPh_3)_3Ir(CO)H$ (0.005 mmol), $Py(CH_2PPh_2)_2$ (0.0075 mmol), H_2 (10 bar), 140 °C, THF (4 mL).

9-2 Reaction process for the catalytic hydrogenation of 1,3-bis(4-chlorophenyl)urea

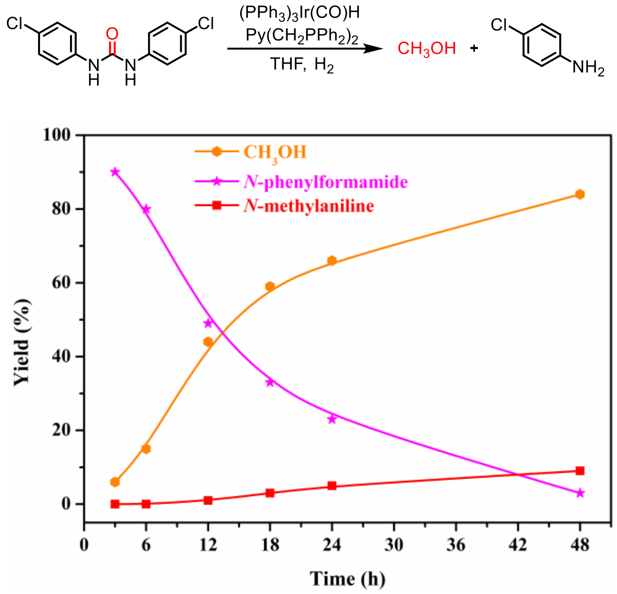

Figure S19. Reaction conditions: 1,3-bis(4-chlorophenyl)urea) (2 mmol), $(PPh_3)_3Ir(CO)H$ (0.08 mmol), $Py(CH_2PPh_2)_2$ (0.10 mmol), H_2 (30 bar), 150 °C, THF (4 mL).

9-3 Reaction process for the catalytic hydrogenation of 1,3-diphenylurea in the presence of methanol as cosolvent

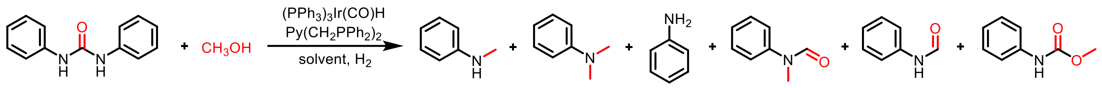

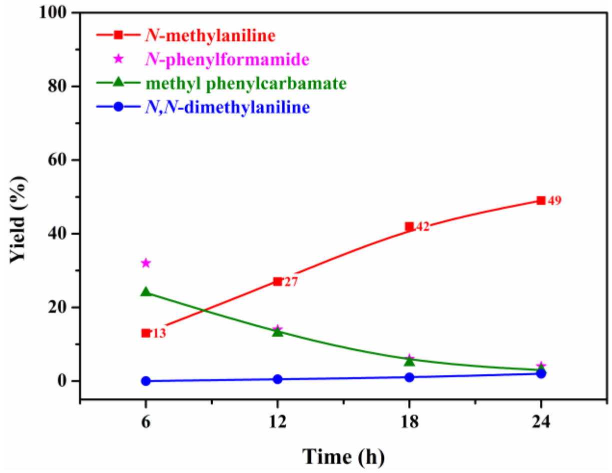

Figure S20. Reaction conditions: 1,3-diphenylurea (2 mmol), $(PPh_3)_3Ir(CO)H$ (0.04 mmol), $Py(CH_2PPh_2)_2$ (0.06 mmol), H_2 (30 bar), solvent (0.3 mL methanol and 3.7 mL THF), reaction temperature (180 °C), reaction times (0-24 h).

(a)

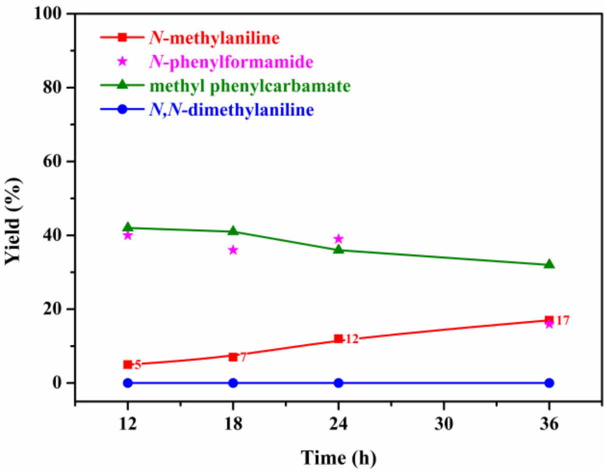

Figure S21. Reaction conditions: 1,3-diphenylurea (2 mmol), $(PPh_3)_3Ir(CO)H$ (0.04 mmol), $Py(CH_2PPh_2)_2$ (0.06 mmol), H_2 (30 bar), solvent (0.3 mL methanol and 3.7 mL THF), reaction temperature (160 °C), reaction times (0-36 h).

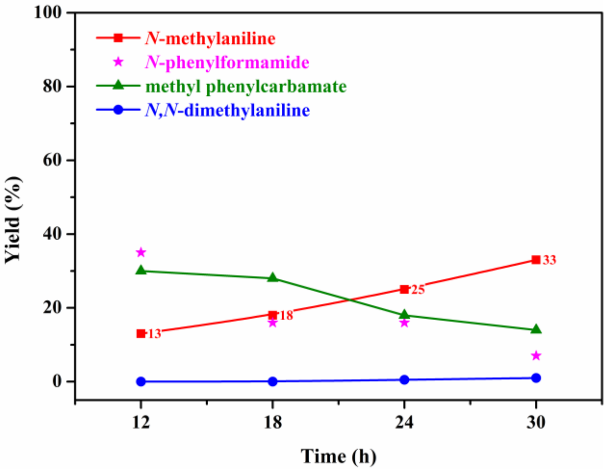

Figure S22. Reaction conditions: 1,3-diphenylurea (2 mmol), $(PPh_3)_3Ir(CO)H$ (0.04 mmol), $Py(CH_2PPh_2)_2$ (0.06 mmol), H_2 (30 bar), solvent (0.3 mL methanol and 3.7 mL THF), reaction temperature (170 °C), reaction times (0-30 h).

(b)

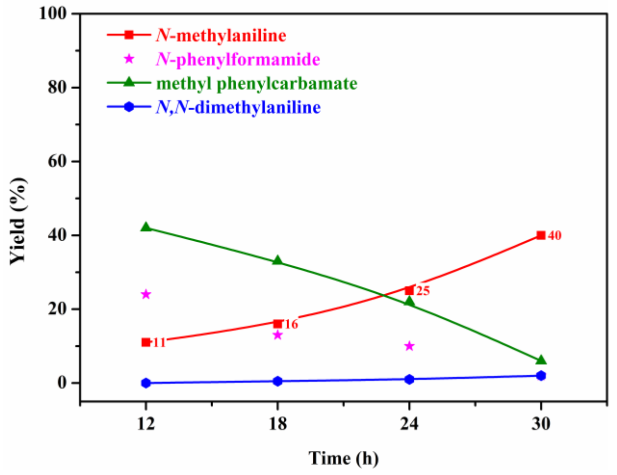

Figure S23. Reaction conditions: 1,3-diphenylurea (2 mmol), $(PPh_3)_3Ir(CO)H$ (0.02 mmol), $Py(CH_2PPh_2)_2$ (0.03 mmol), H_2 (30 bar), solvent (0.3 mL methanol and 3.7 mL THF), reaction temperature (180 °C), reaction times (0-30 h).

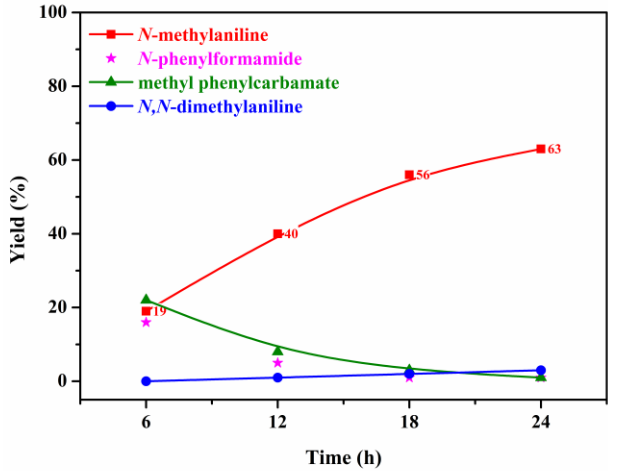

Figure S24. Reaction conditions: 1,3-diphenylurea (2 mmol), $(PPh_3)_3Ir(CO)H$ (0.08 mmol), $Py(CH_2PPh_2)_2$ (0.12 mmol), H_2 (30 bar), solvent (0.3 mL methanol and 3.7 mL THF), reaction temperature (180 °C), reaction times (0-24 h).

(c)

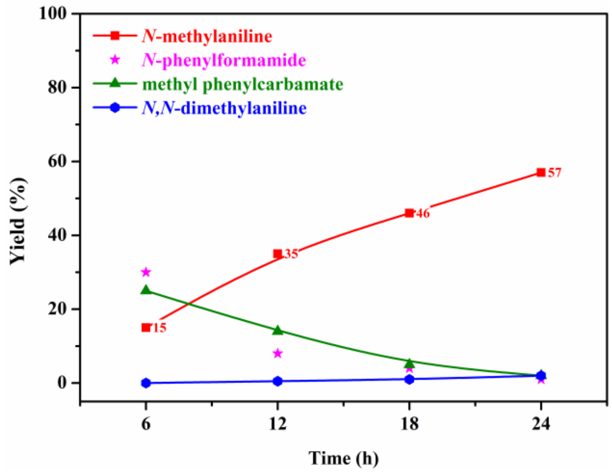

Figure S25. Reaction conditions: 1,3-diphenylurea (1.5 mmol), $(PPh_3)_3Ir(CO)H$ (0.04 mmol), $Py(CH_2PPh_2)_2$ (0.06 mmol), H_2 (30 bar), solvent (0.3 mL methanol and 3.7 mL THF), reaction temperature (180 °C), reaction times (0-24 h).

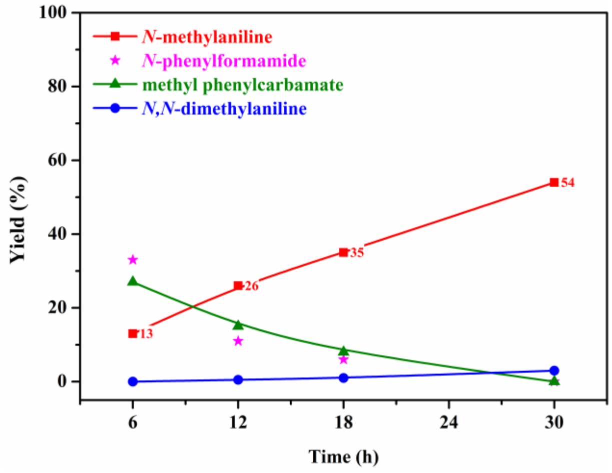

Figure S26. Reaction conditions: 1,3-diphenylurea (2.5 mmol), $(PPh_3)_3Ir(CO)H$ (0.04 mmol), $Py(CH_2PPh_2)_2$ (0.06 mmol), H_2 (30 bar), solvent (0.3 mL methanol and 3.7 mL THF), reaction temperature (180 °C), reaction times (0-30 h).

(d)

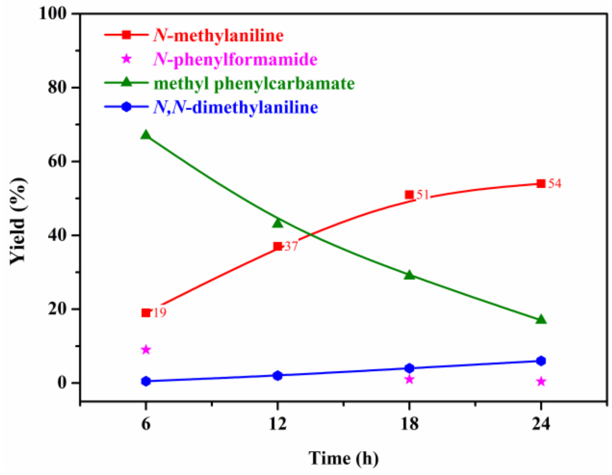

Figure S27. Reaction conditions: 1,3-diphenylurea (2 mmol), $(PPh_3)_3Ir(CO)H$ (0.04 mmol), $Py(CH_2PPh_2)_2$ (0.06 mmol), H_2 (30 bar), solvent (1.0 mL methanol and 3.0 mL THF), reaction temperature (180 °C), reaction times (0-24 h).

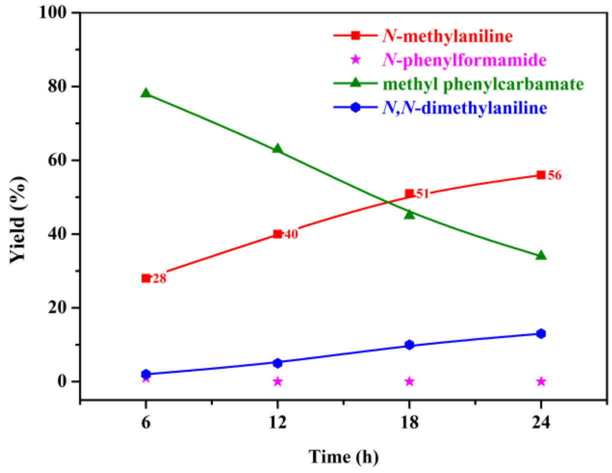

Figure S28. Reaction conditions: 1,3-diphenylurea (2 mmol), $(PPh_3)_3Ir(CO)H$ (0.04 mmol), $Py(CH_2PPh_2)_2$ (0.06 mmol), H_2 (30 bar), solvent (2.0 mL methanol and 2.0 mL THF), reaction temperature (180 °C), reaction times (0-24 h).

10. Two-step process for conversion of urea derivatives and carbamates to methylamines under mild reaction conditions

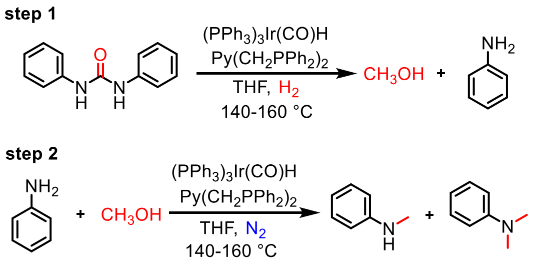

Figure S29. Alternative two-step synthesis route for methylamines at mild reaction conditions.

11. Possible reaction pathways

11-1 Iridium-catalyzed hydrogenation of carbamates and urea derivatives to formamides

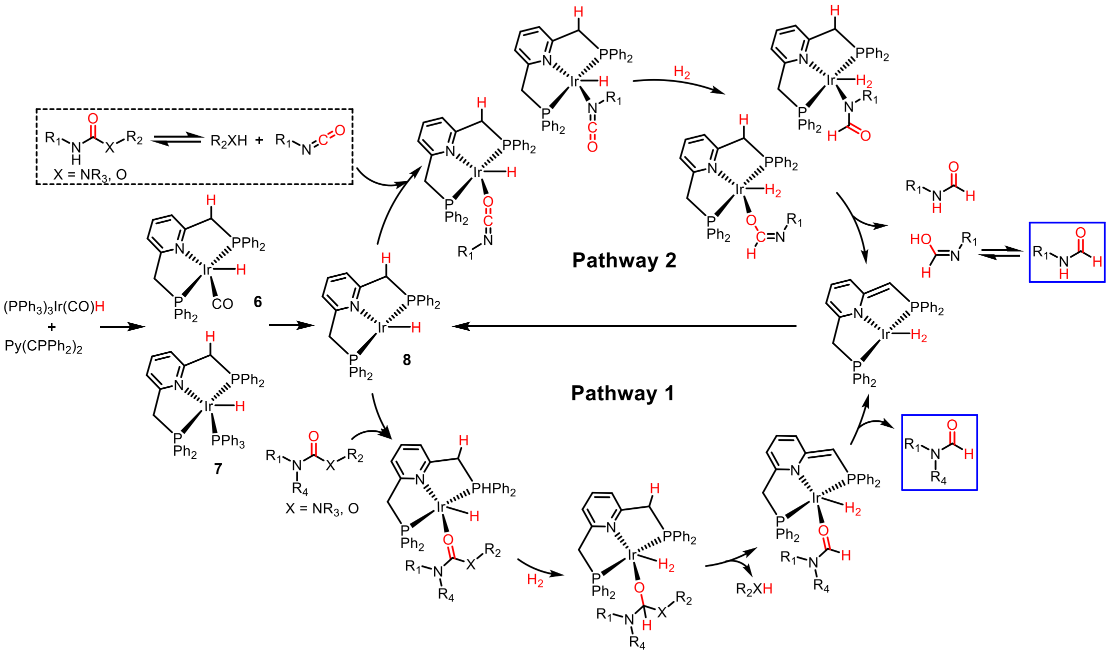

Figure S30. Proposed reaction mechanism for Iridium-catalyzed hydrogenation of carbamates and urea derivatives to formamides.

11-2 Iridium-catalyzed hydrogenation of carbamates and urea derivatives to methanol

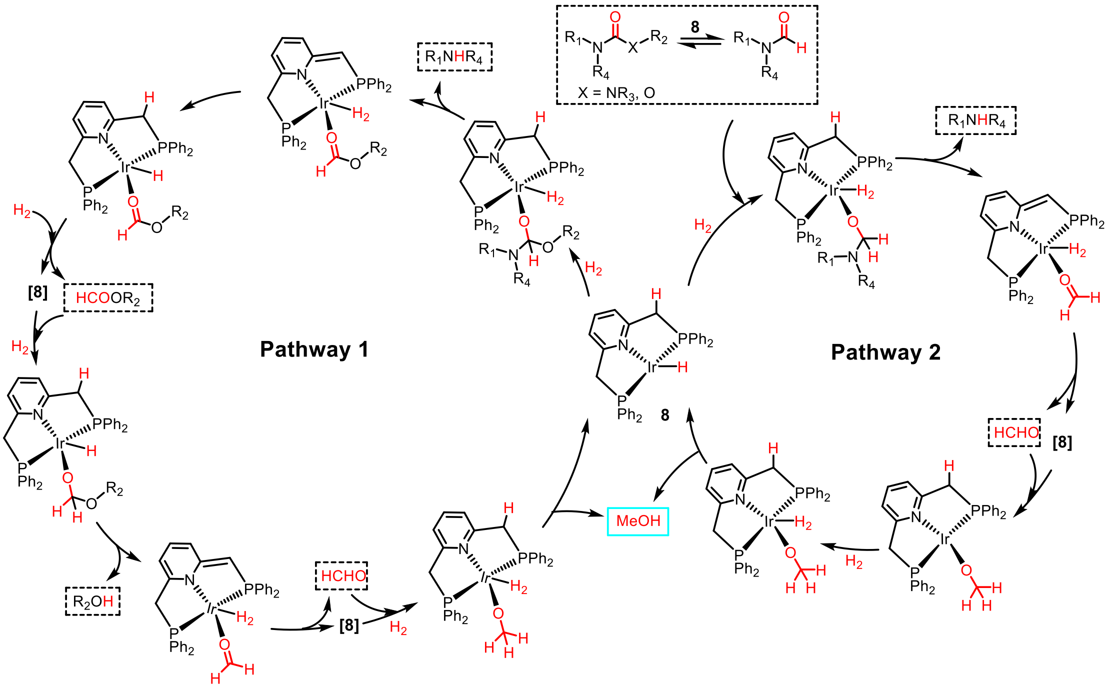

Figure S31. Proposed reaction mechanism for Iridium-catalyzed hydrogenation of carbamates and urea derivatives to methanol.

11-3 Iridium-catalyzed hydrogenation of carbamates and urea derivatives to methylamines

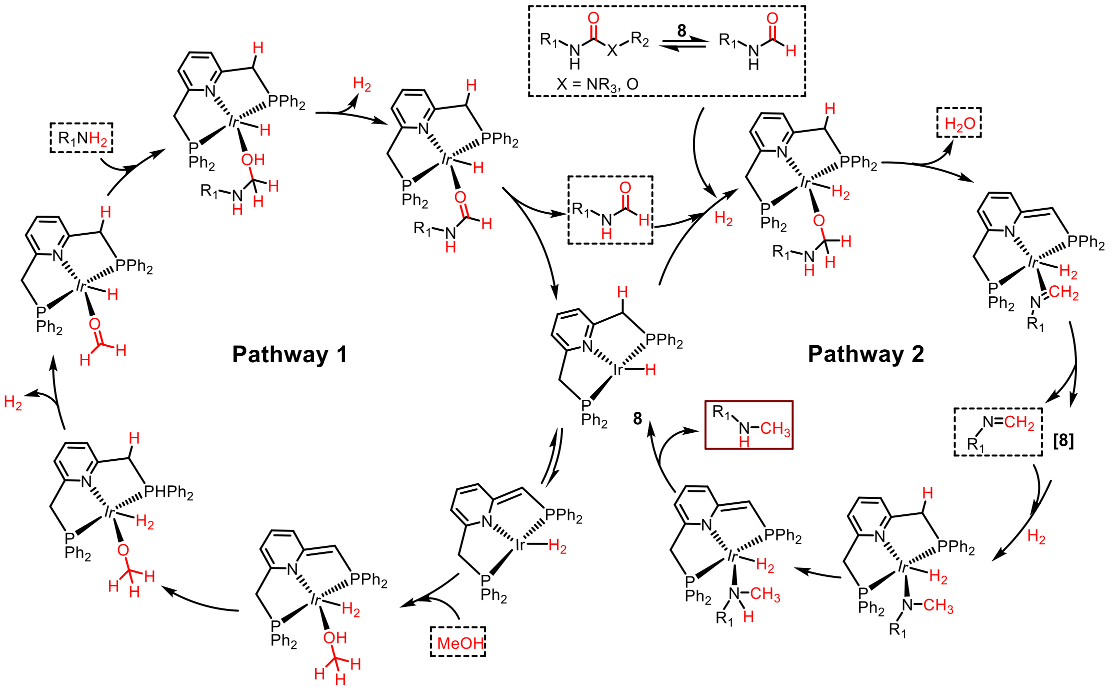

Figure S32. Proposed reaction mechanism for Iridium-catalyzed hydrogenation of carbamates and urea derivatives to methylamines.

12. Analysis of organic compounds

12-1 GC-MS Data of Products

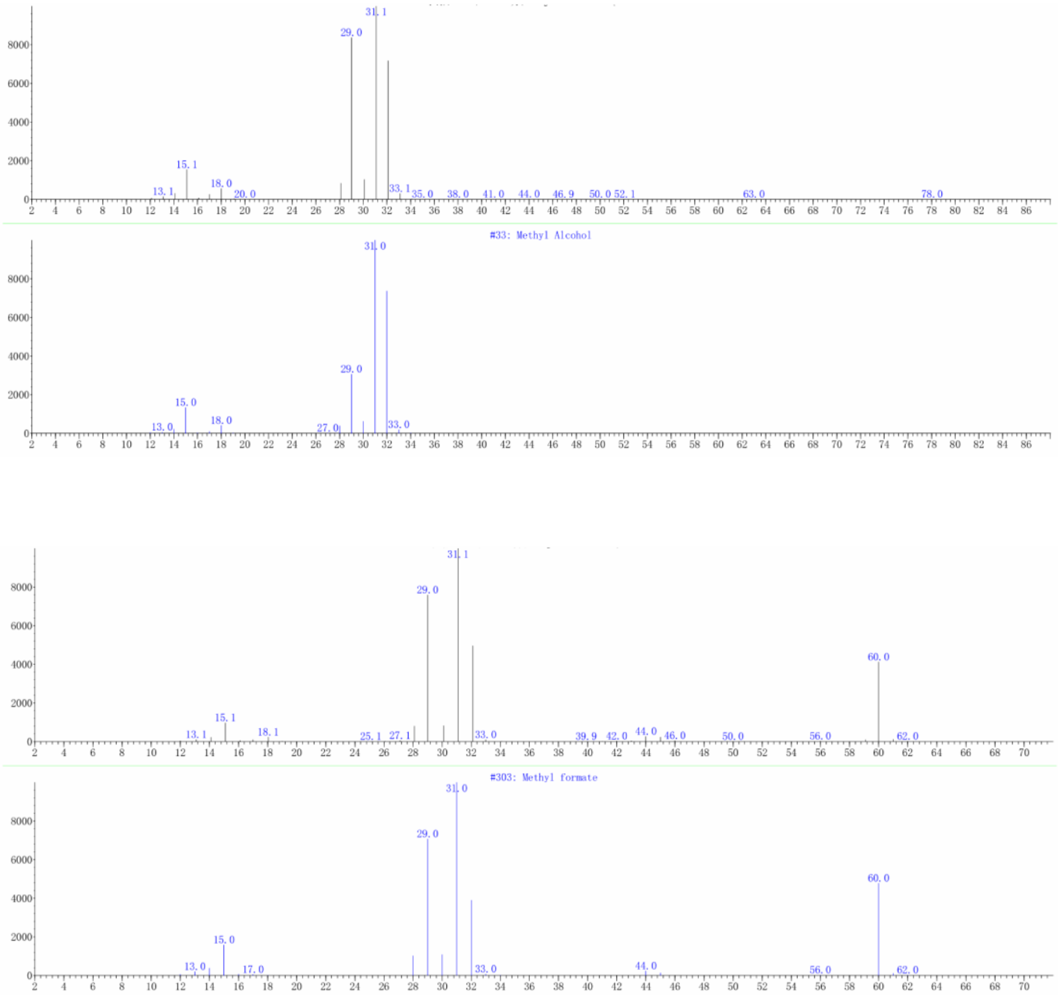

Figure S33. GC-MS spectra of the CH_3OH and $HCOOCH_3$ for hydrogenation of methyl phenylcarbamate.

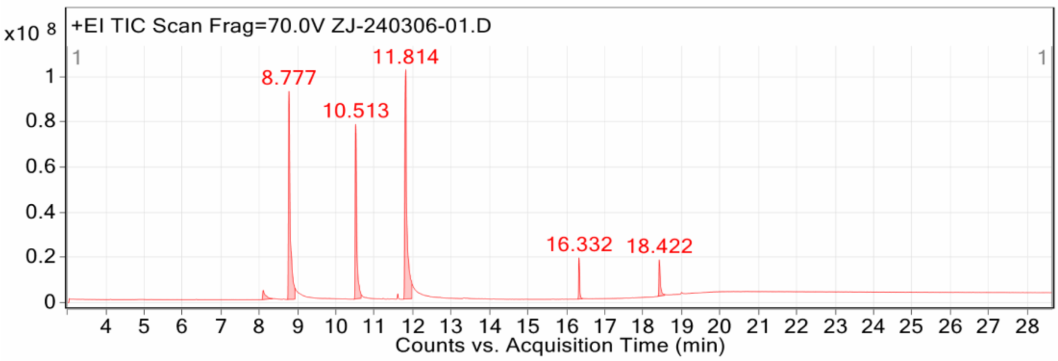
x10 8
+EI TIC Scan Frag=70.0V ZJ-240306-01.D
8.777
10.513
11.814
16.332
18.422
Counts vs. Acquisition Time (min)

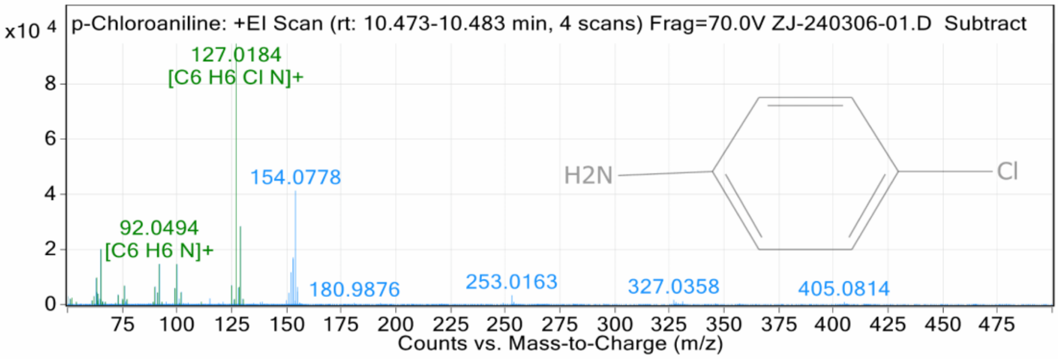
x10 4
p-Chloroaniline: +EI Scan (rt: 10.473-10.483 min, 4 scans) Frag=70.0V ZJ-240306-01.D Subtract
127.0184
[C6 H6 Cl N]+
154.0778
92.0494
[C6 H6 N]+
180.9876
253.0163
327.0358
405.0814
H2N
Cl
Counts vs. Mass-to-Charge (m/z)

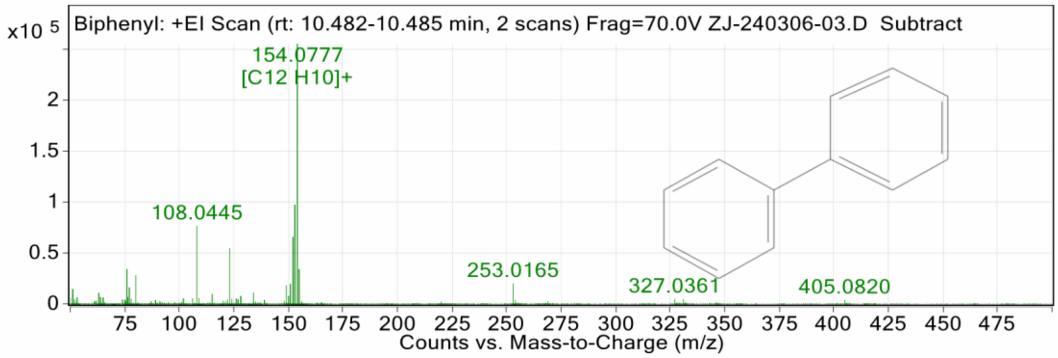
x10 5
Biphenyl: +EI Scan (rt: 10.482-10.485 min, 2 scans) Frag=70.0V ZJ-240306-03.D Subtract
154.0777
[C12 H10]+
108.0445
253.0165
327.0361
405.0820
Counts vs. Mass-to-Charge (m/z)

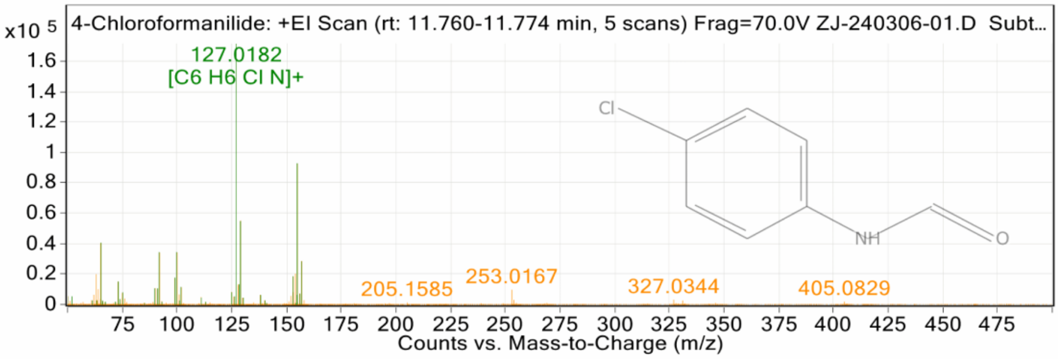
x10 5
4-Chloroformanilide: +EI Scan (rt: 11.760-11.774 min, 5 scans) Frag=70.0V ZJ-240306-01.D Subt...
127.0182
[C6 H6 Cl N]+
Cl
NH
O
205.1585
253.0167
327.0344
405.0829
Counts vs. Mass-to-Charge (m/z)

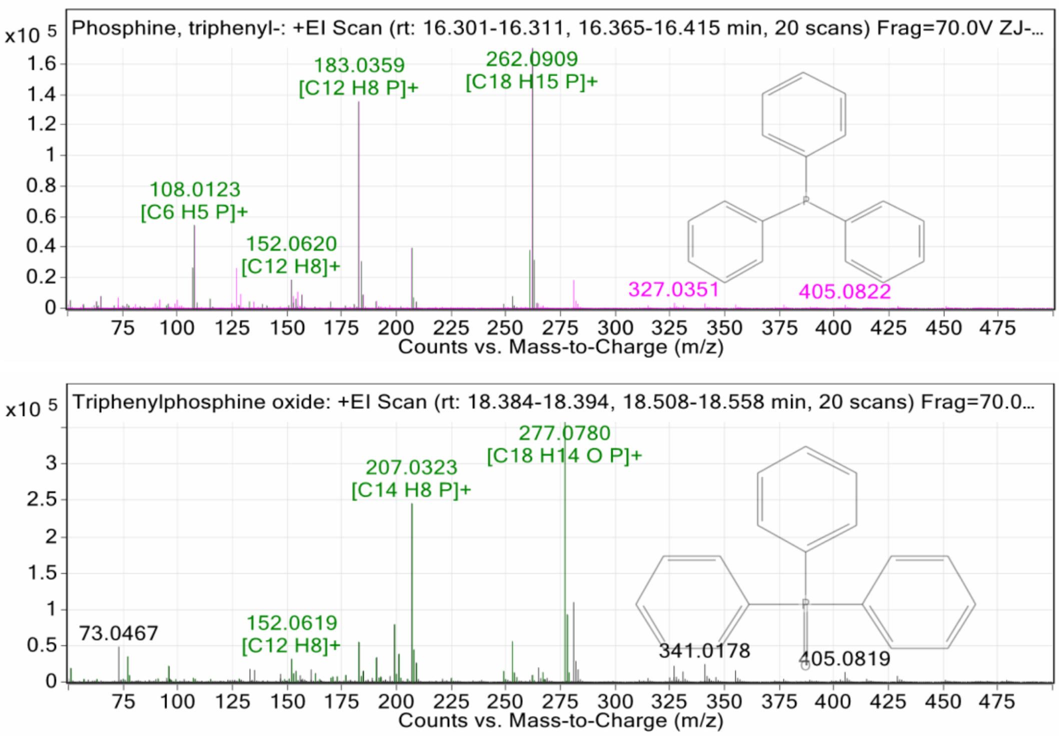

Figure S34. GC-MS spectrum of the crude reaction mixture of hydrogenation of 1,3-bis(4-chlorophenyl)urea (**1a**) with biphenyl as internal standard.

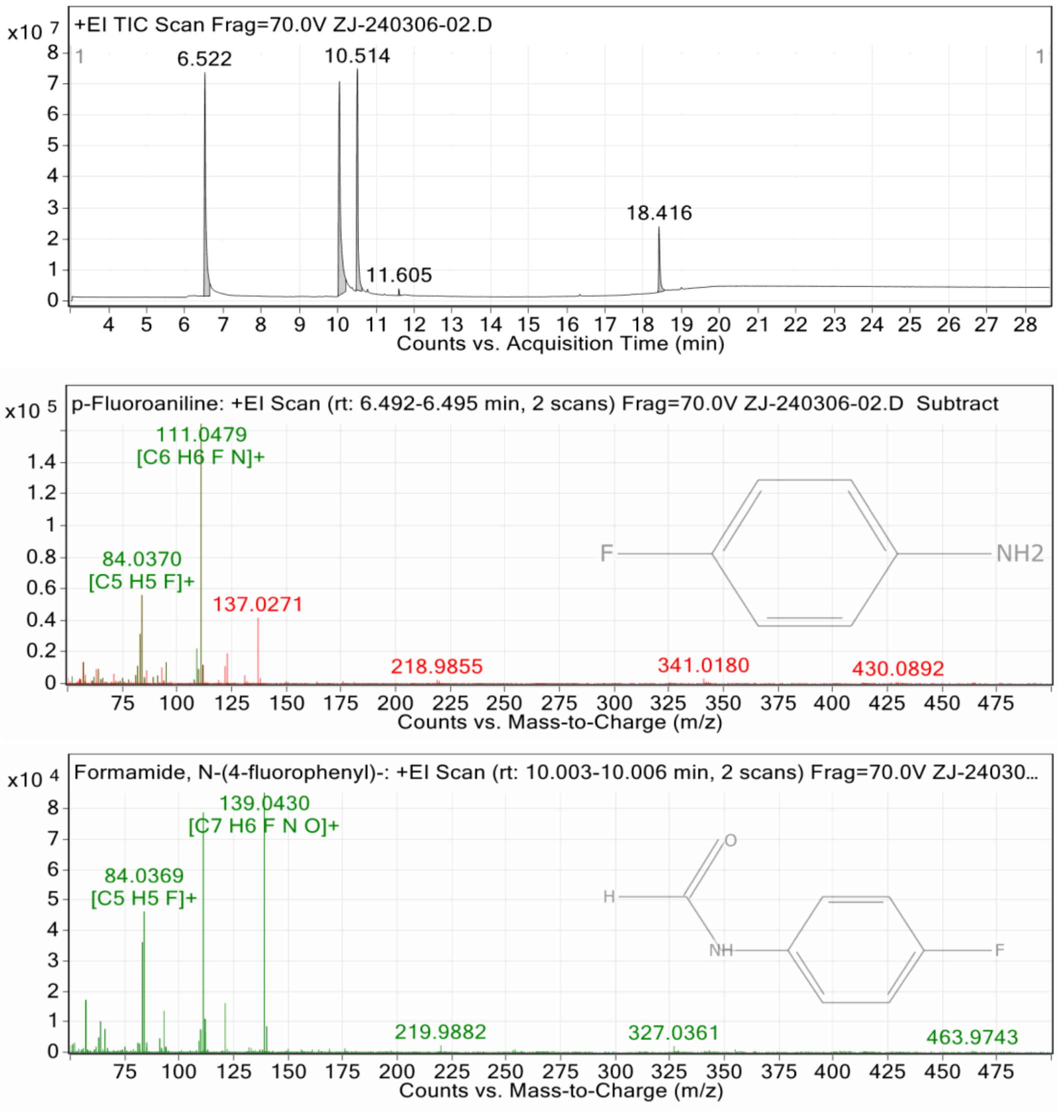

Figure S35. GC-MS spectrum of the crude reaction mixture of hydrogenation of 1,3-bis(4-fluorophenyl)urea (**1b**) with biphenyl as internal standard.

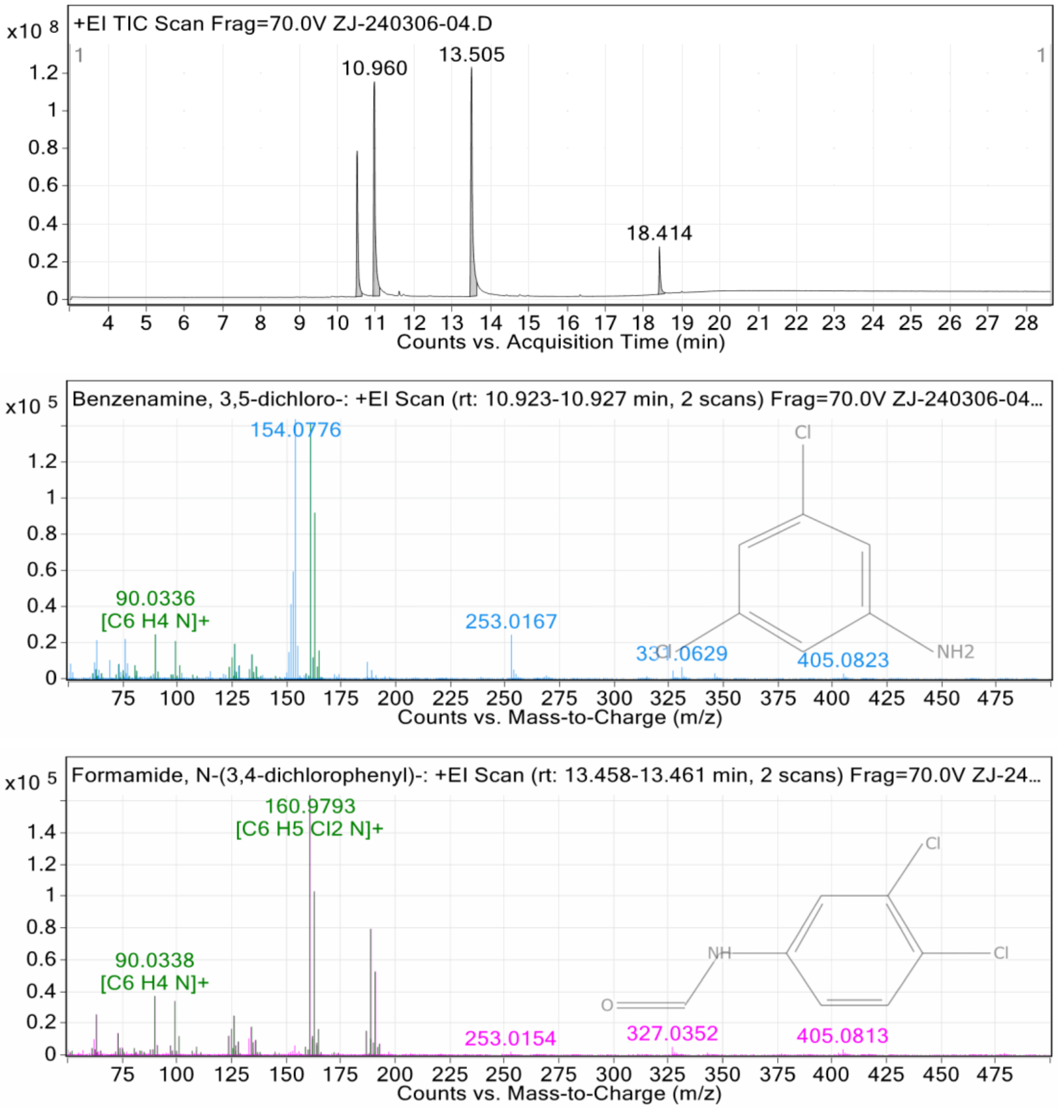

Figure S36. GC-MS spectrum of the crude reaction mixture of hydrogenation of 1,3-bis(3,4-dichlorophenyl)urea (**1c**) with biphenyl as internal standard.

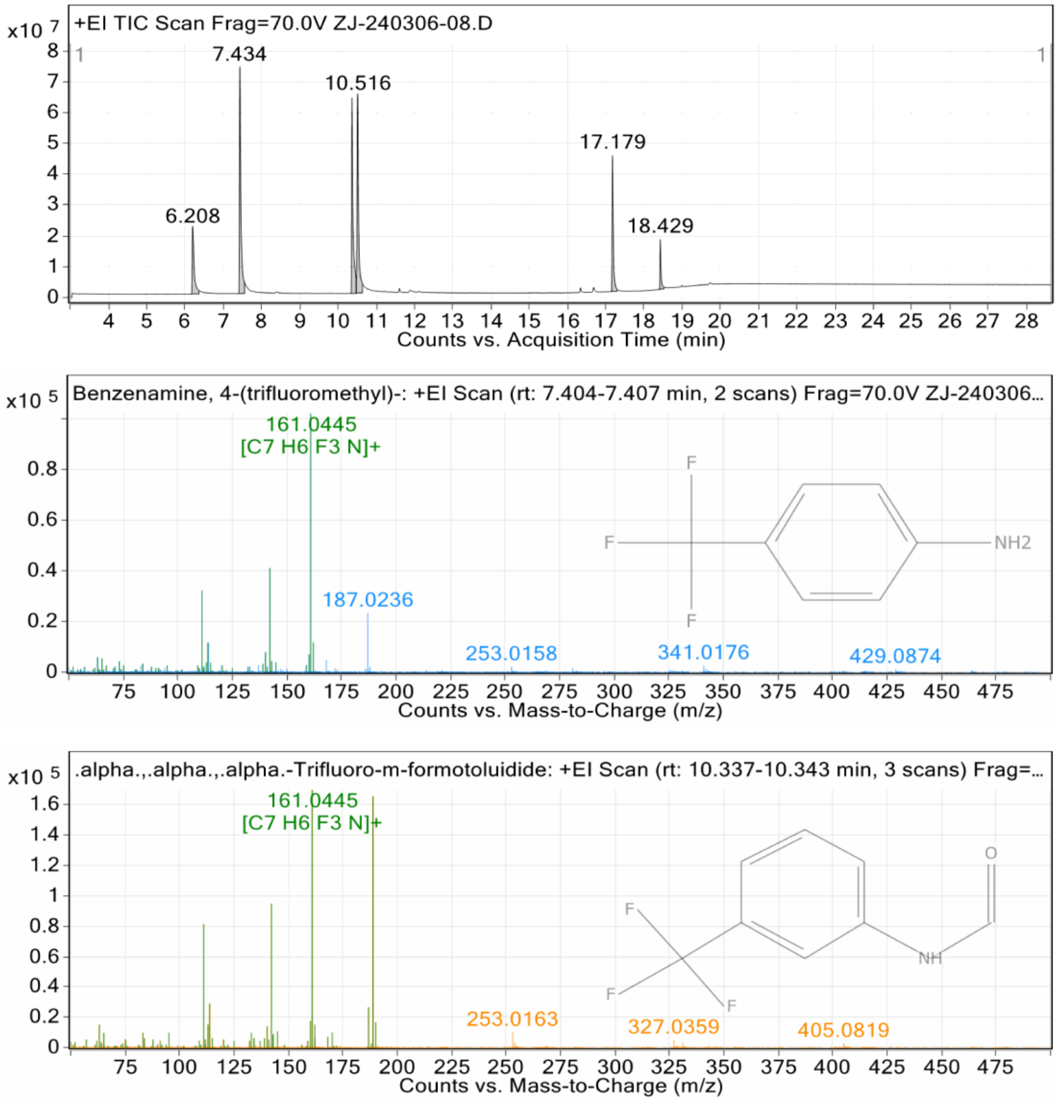

Figure S37. GC-MS spectrum of the crude reaction mixture of hydrogenation of 1,3-bis[4-(trifluoromethyl)phenyl]urea (**1d**) with biphenyl as internal standard.

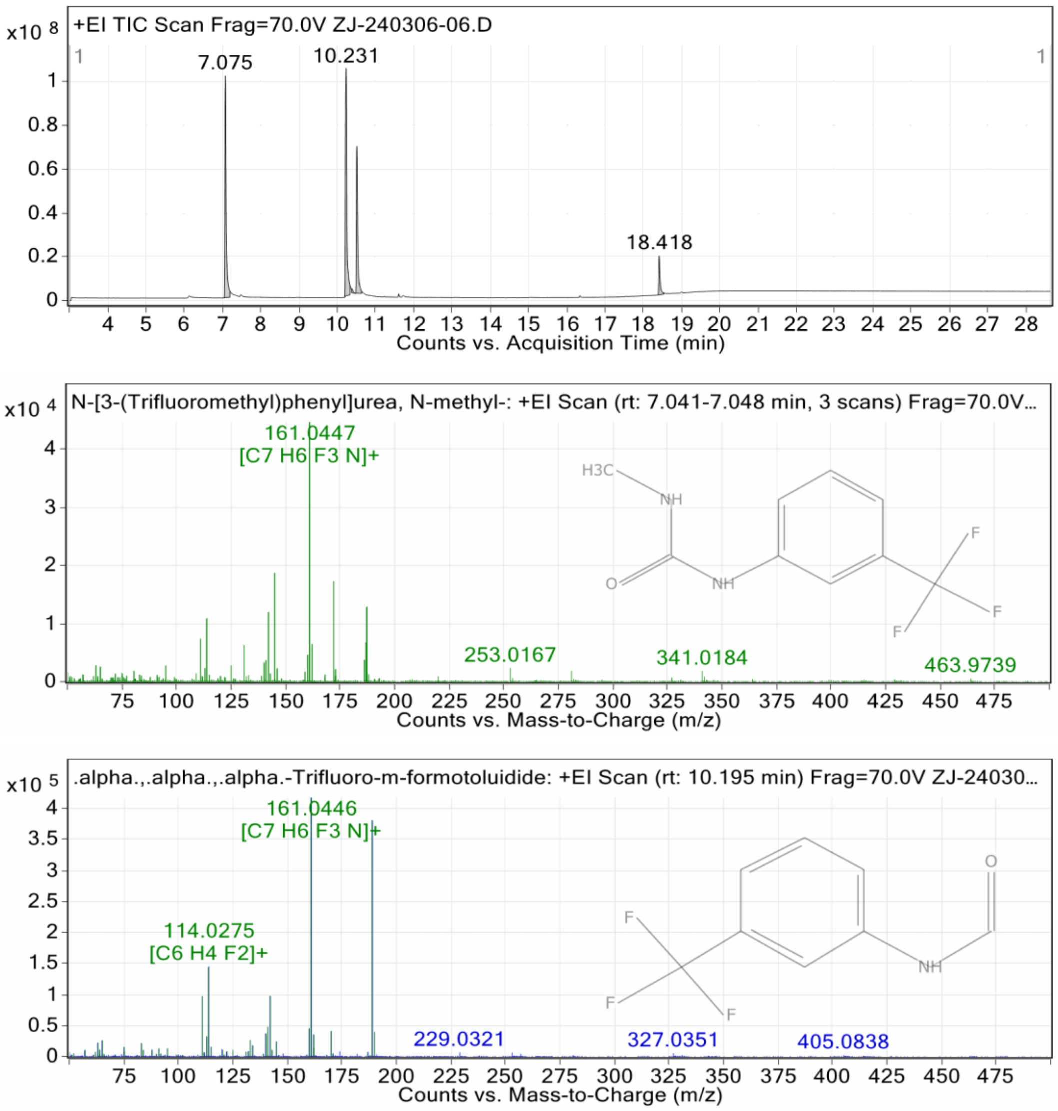

Figure S38. GC-MS spectrum of the crude reaction mixture of hydrogenation of 1,3-bis[3-(trifluoromethyl)phenyl]urea (**1e**) with biphenyl as internal standard.

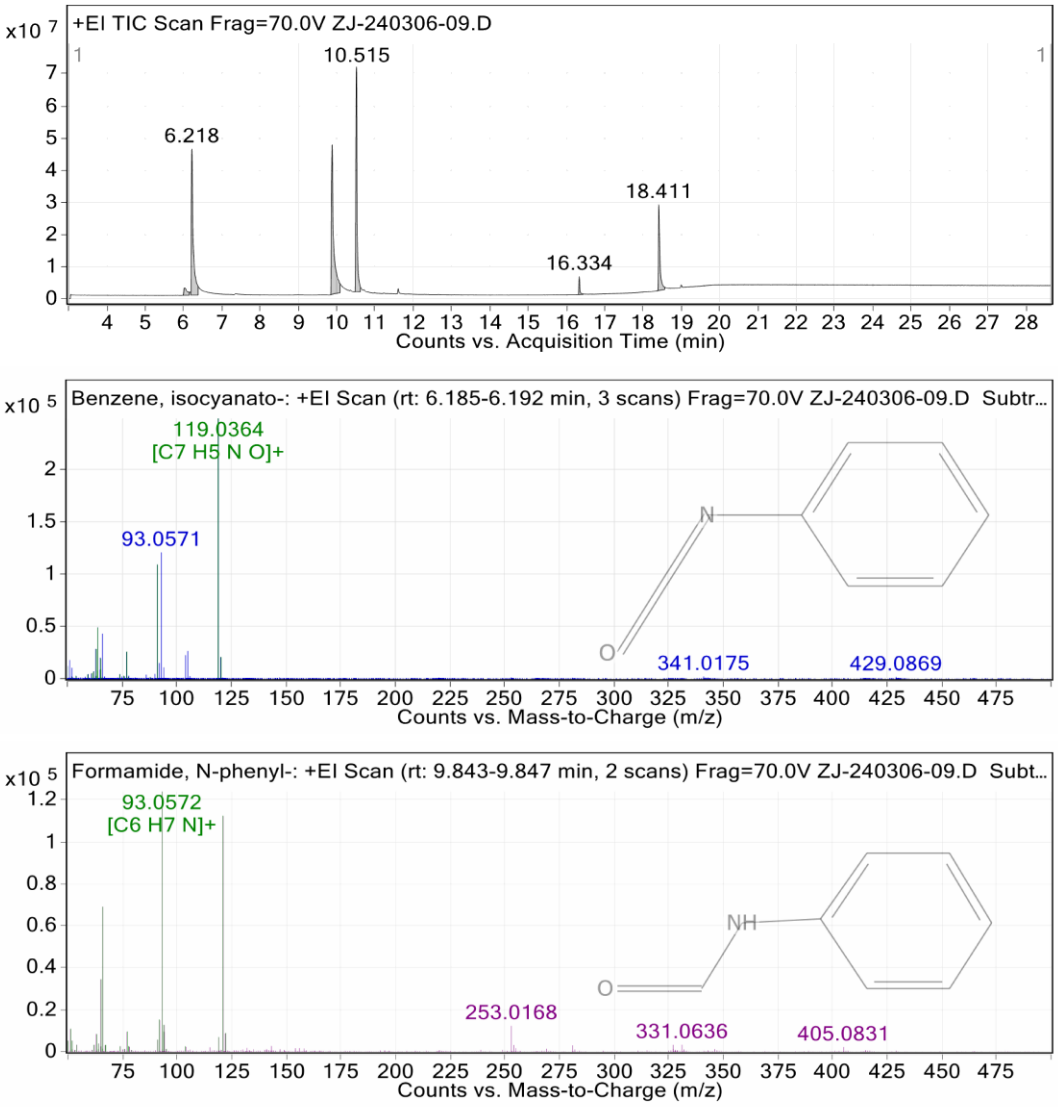

Figure S39. GC-MS spectrum of the crude reaction mixture of hydrogenation of 1,3-diphenylurea (**1g**) with biphenyl as internal standard.

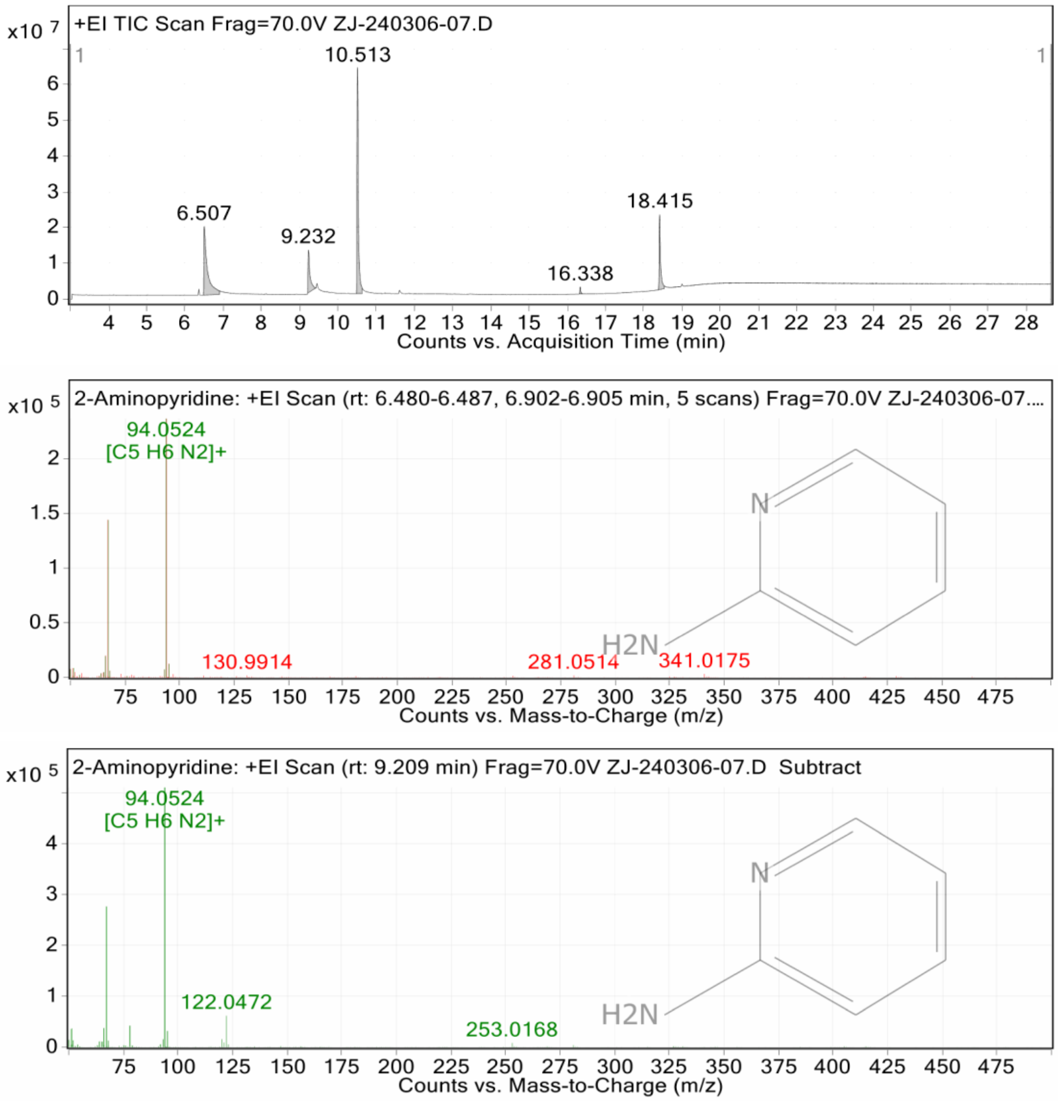

Figure S40. GC-MS spectrum of the crude reaction mixture of hydrogenation of 1,3-di(pyridin-2-yl)urea (**1h**) with biphenyl as internal standard.

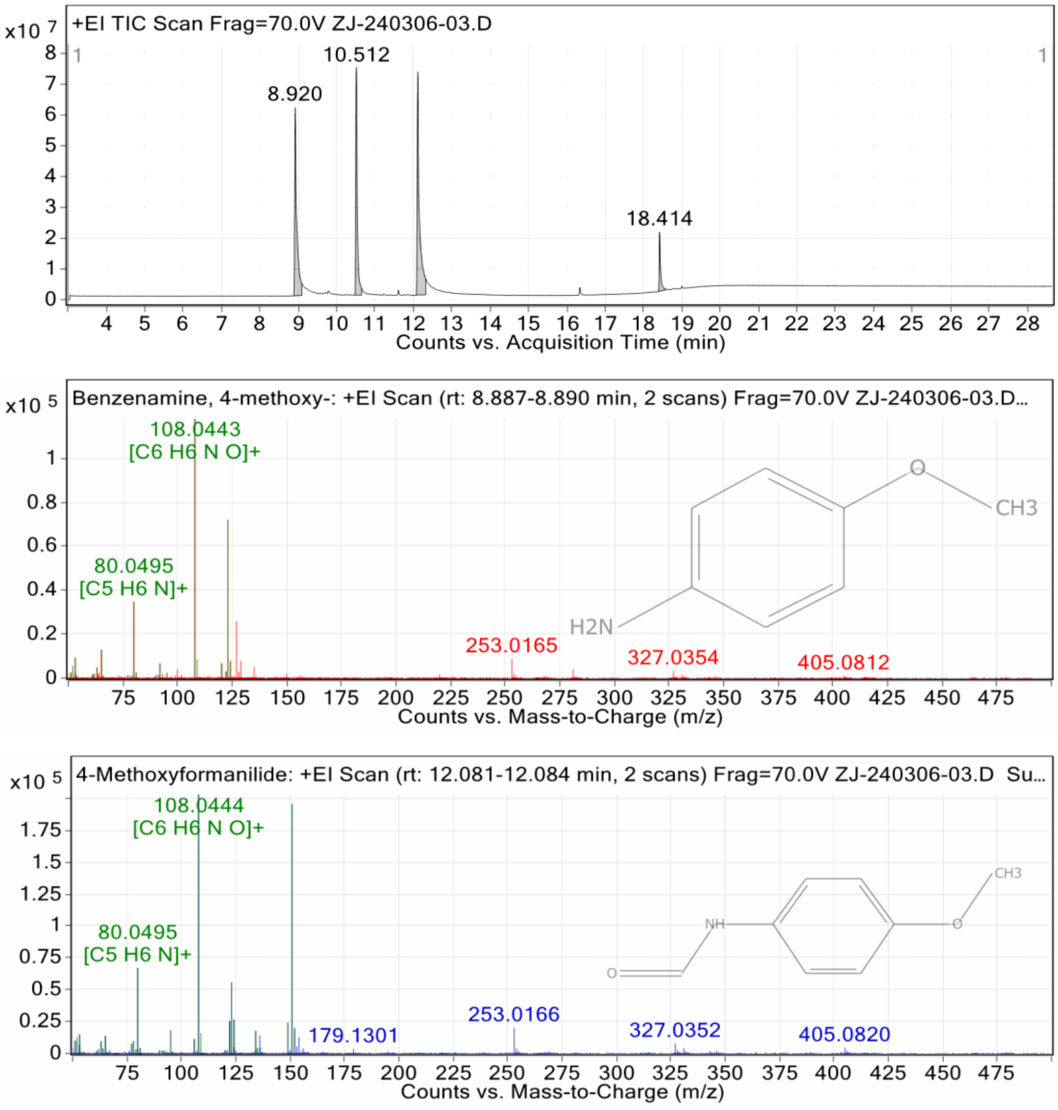

Figure S41. GC-MS spectrum of the crude reaction mixture of hydrogenation of 1,3-bis(4-methoxyphenyl)urea (**1j**) with biphenyl as internal standard.

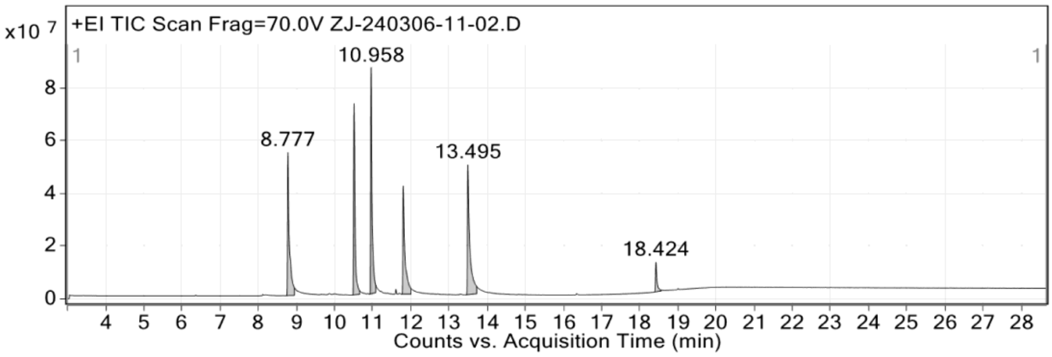

x10 7
+EI TIC Scan Frag=70.0V ZJ-240306-11-02.D
10.958
8.777
13.495
18.424
Counts vs. Acquisition Time (min)

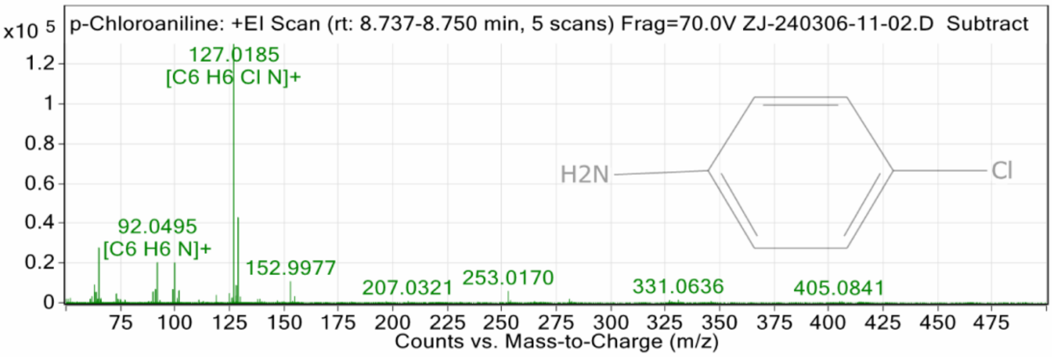

x10 5
p-Chloroaniline: +EI Scan (rt: 8.737-8.750 min, 5 scans) Frag=70.0V ZJ-240306-11-02.D Subtract
127.0185
[C6 H6 Cl N]+
92.0495
[C6 H6 N]+
152.9977
207.0321
253.0170
331.0636
405.0841
H2N
Cl
Counts vs. Mass-to-Charge (m/z)

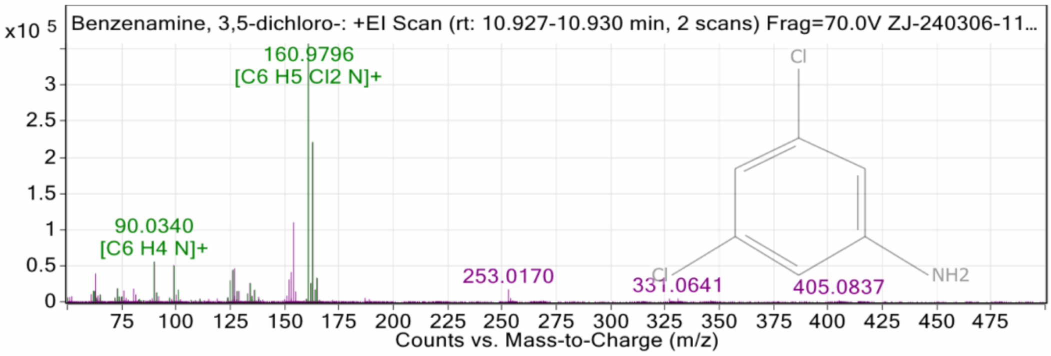

x10 5
Benzenamine, 3,5-dichloro-: +EI Scan (rt: 10.927-10.930 min, 2 scans) Frag=70.0V ZJ-240306-11...
160.9796
[C6 H5 Cl2 N]+
90.0340
[C6 H4 N]+
253.0170
331.0641
405.0837
Cl
Cl
NH2
Counts vs. Mass-to-Charge (m/z)

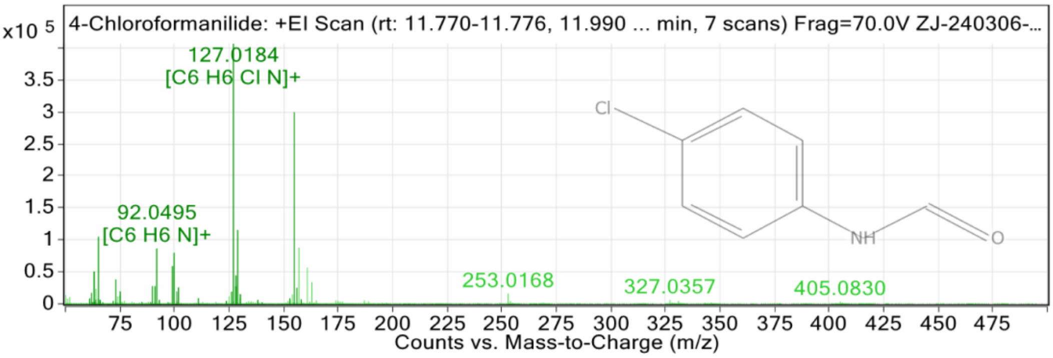

x10 5
4-Chloroformanilide: +EI Scan (rt: 11.770-11.776, 11.990 ... min, 7 scans) Frag=70.0V ZJ-240306-...
127.0184
[C6 H6 Cl N]+
92.0495
[C6 H6 N]+
253.0168
327.0357
405.0830
Cl
NH
O
Counts vs. Mass-to-Charge (m/z)

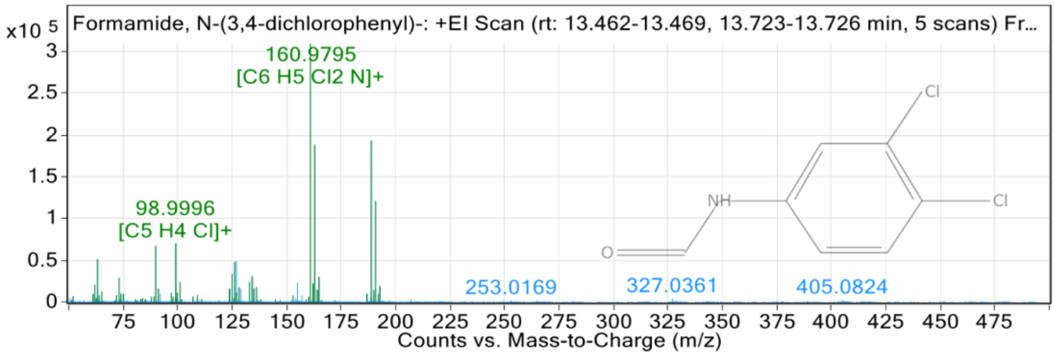

Figure S42. GC-MS spectrum of the crude reaction mixture of hydrogenation of 1-(4-chlorophenyl)-3-(3,4-dichlorophenyl)urea (**1o**) with biphenyl as internal standard.

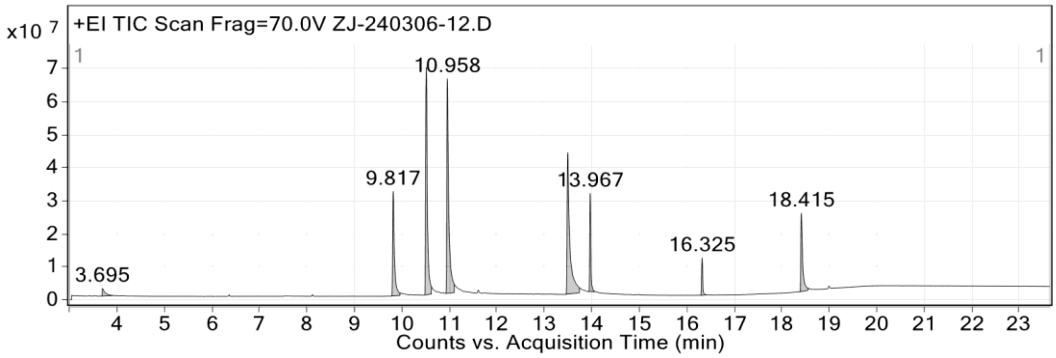

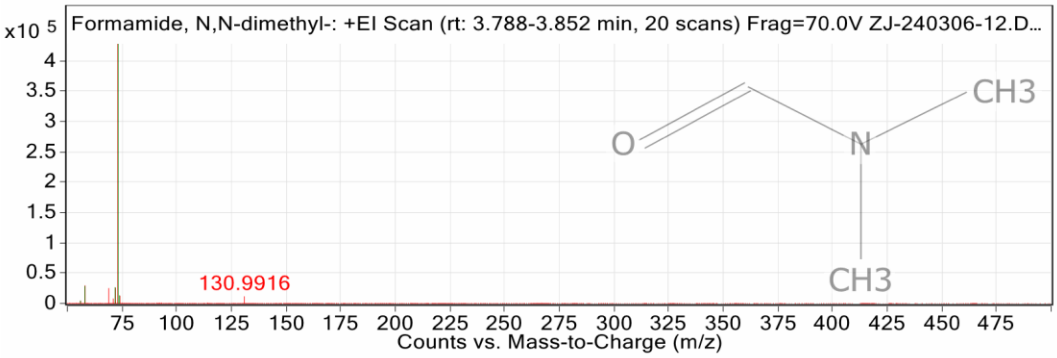

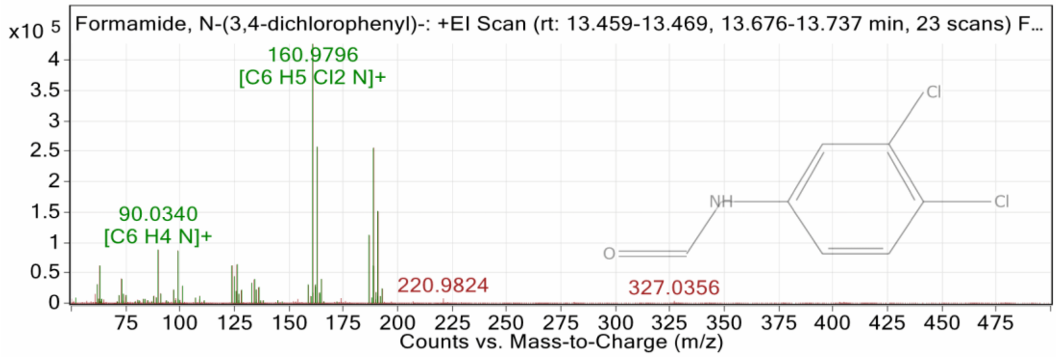

Figure S43. GC-MS spectrum of the crude reaction mixture of hydrogenation of 3-(3,4-dichlorophenyl)-1,1-dimethylurea (**1s**) with biphenyl as internal standard.

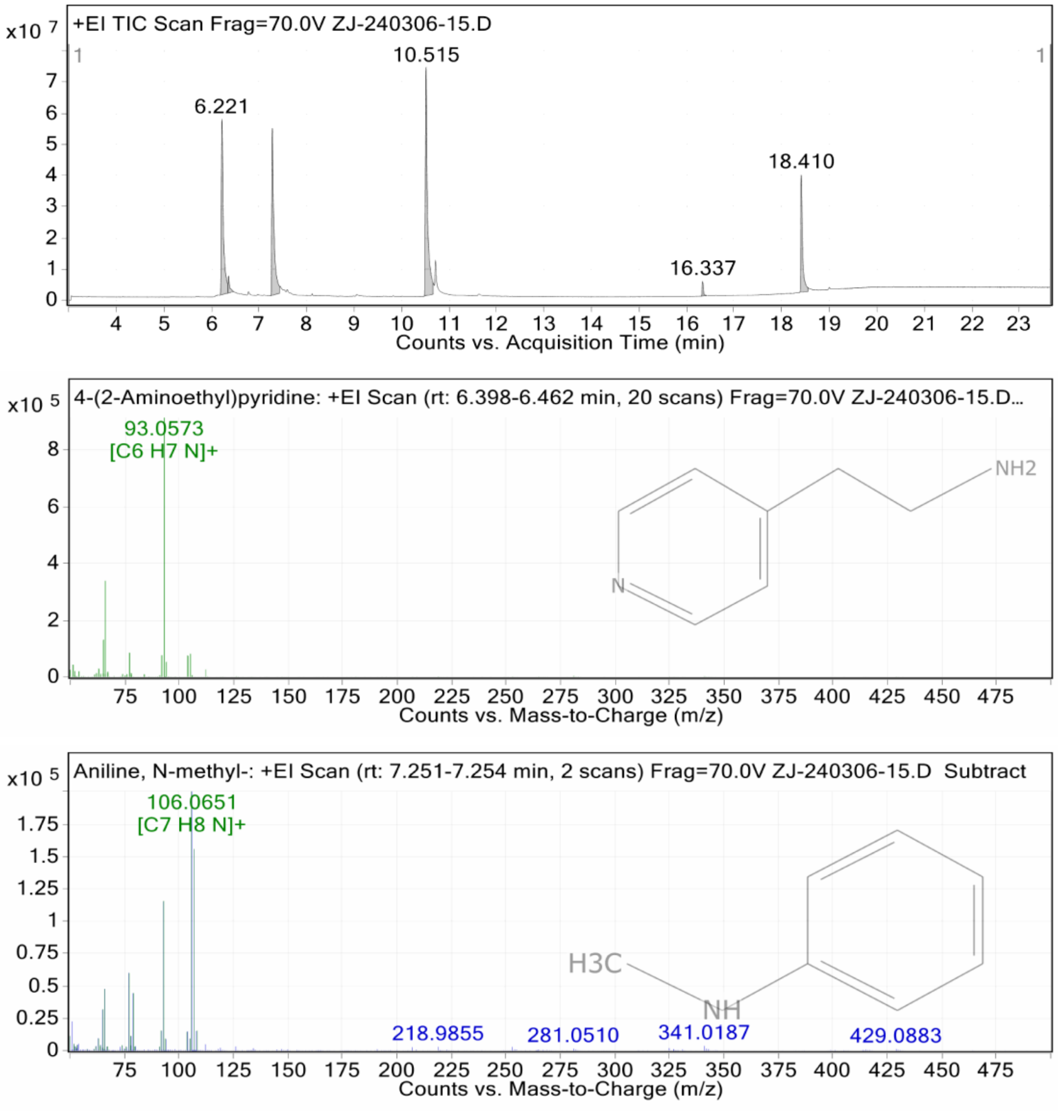

Figure S44. GC-MS spectrum of the crude reaction mixture of hydrogenation of 1,3-diphenylurea in the presence of CH_3OH with biphenyl as internal standard.

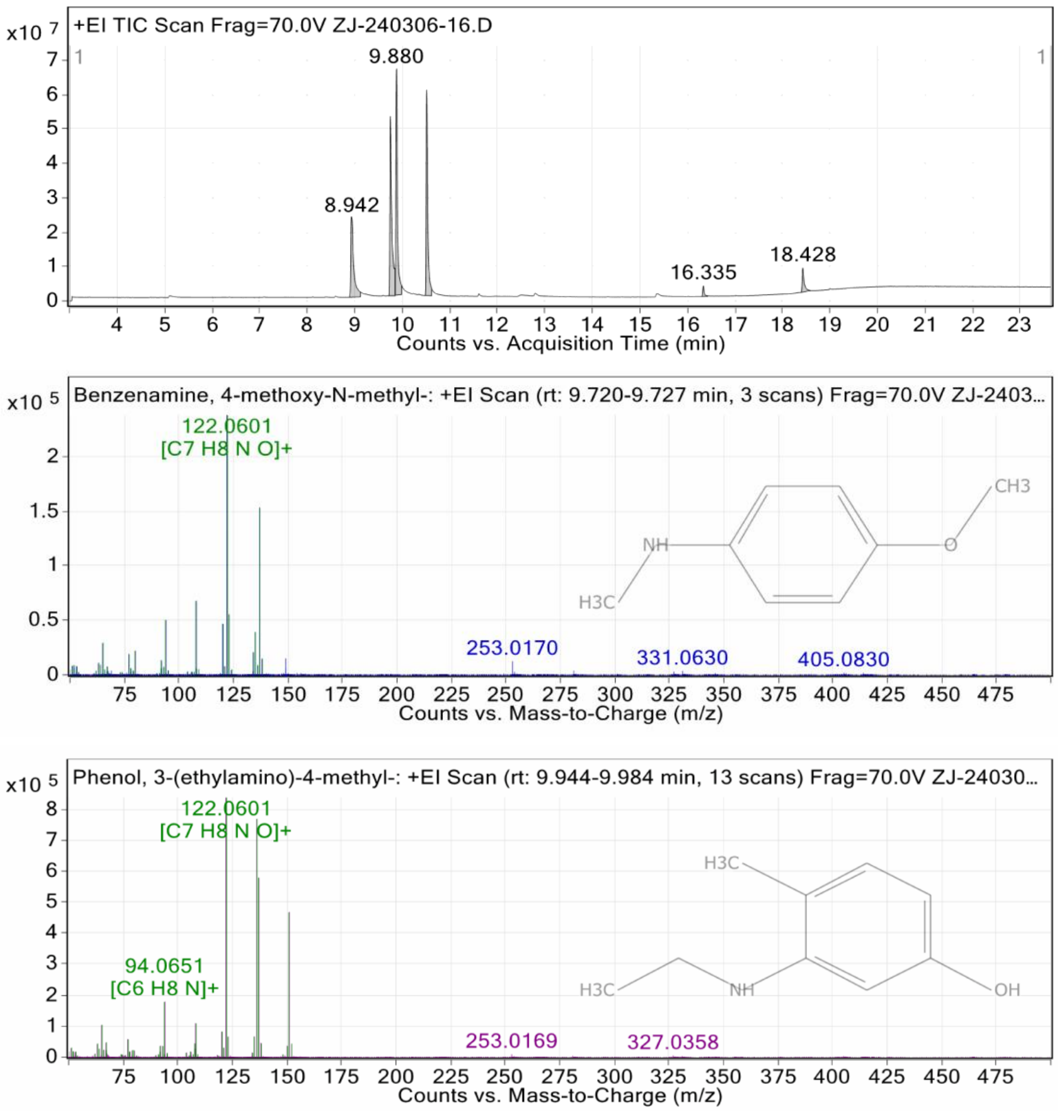

Figure S45. GC-MS spectrum of the crude reaction mixture of hydrogenation of 1,3-bis(4-methoxyphenyl)urea in the presence of CH_3OH with biphenyl as internal standard.

12-2 NMR Data of Isolated Products

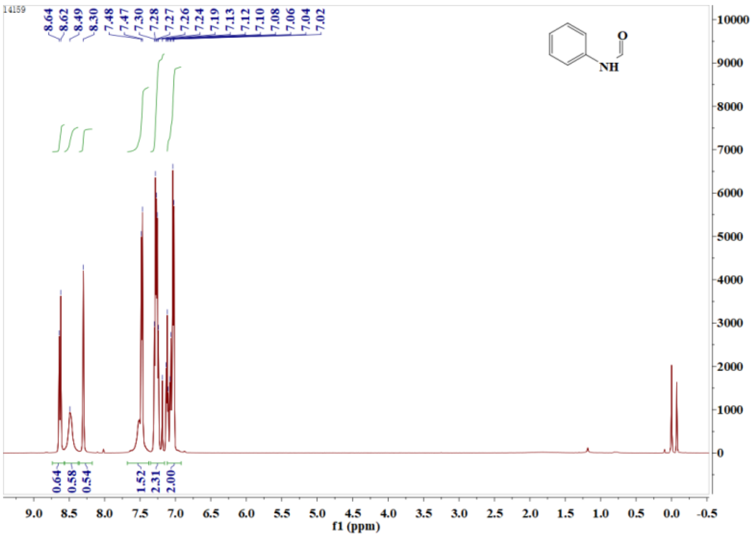

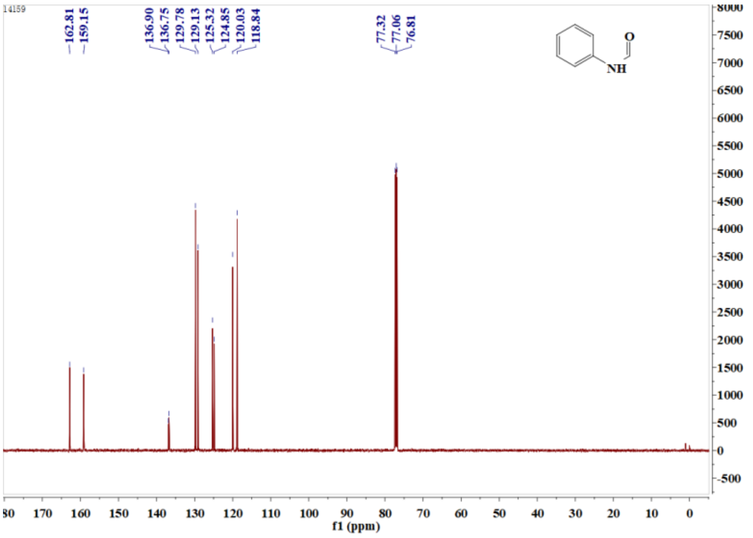

Figure S46. ^{1}H NMR ($CDCl_3$, 500 MHz) spectrum and ^{13}C NMR ($CDCl_3$, 125 MHz) spectrum of the *N*-formanilide.

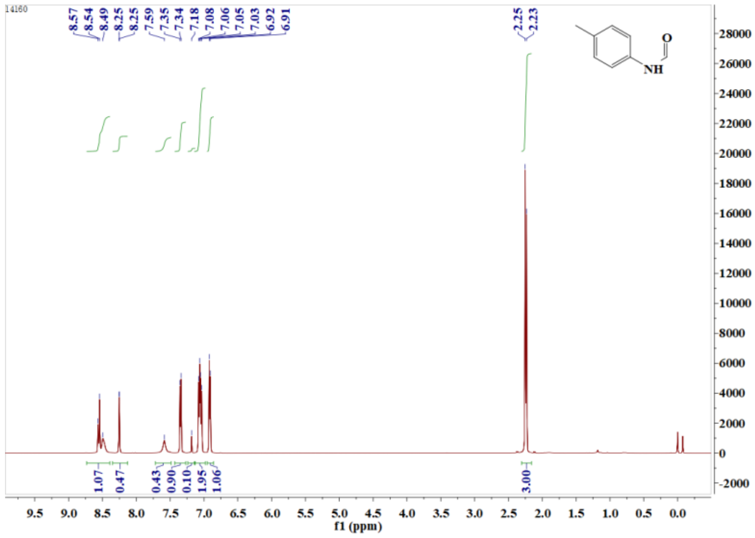

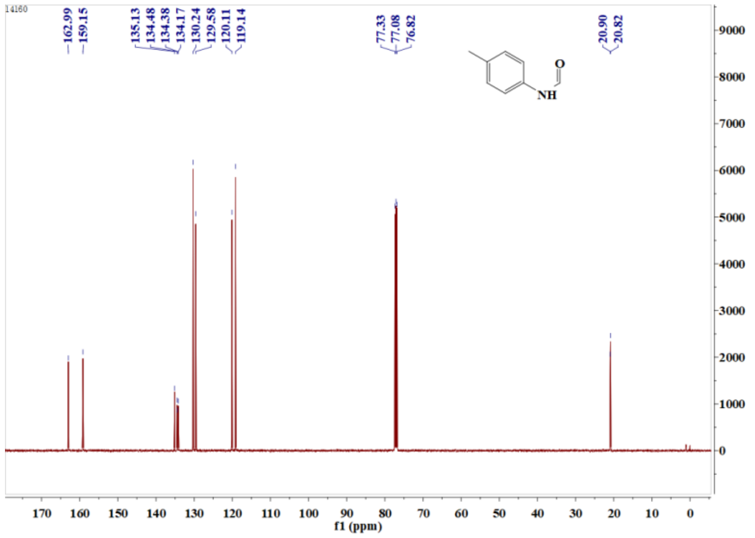

Figure S47. ^{1}H NMR ($CDCl_3$, 500 MHz) spectrum and ^{13}C NMR ($CDCl_3$, 125 MHz) spectrum of the *N*-(4-methylphenyl)formamide.

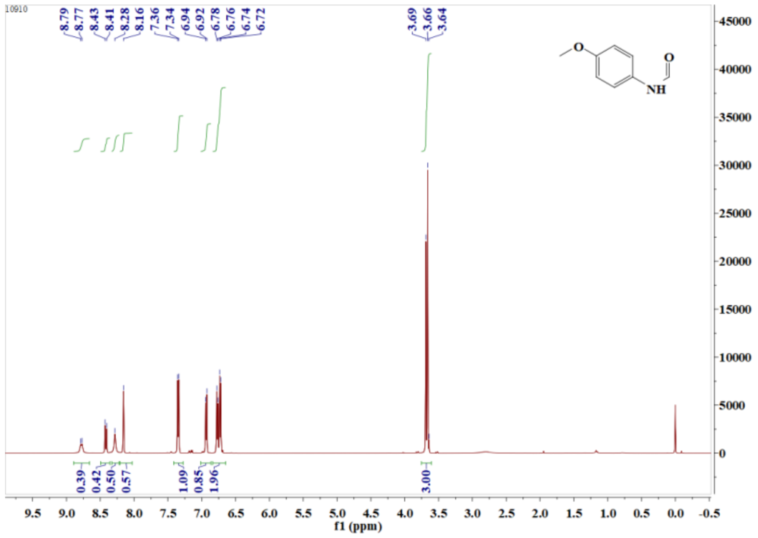

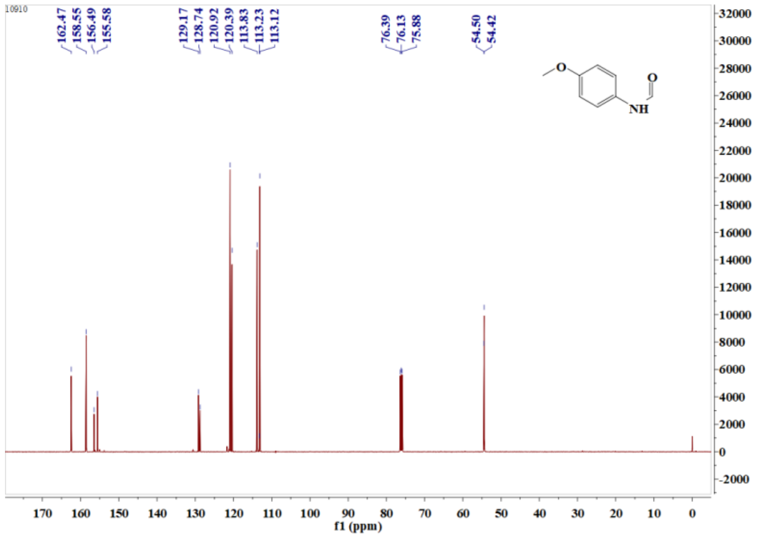

Figure S48. ^{1}H NMR ($CDCl_3$, 500 MHz) spectrum and ^{13}C NMR ($CDCl_3$, 125 MHz) spectrum of the *N*-(4-methoxy)formanilide.

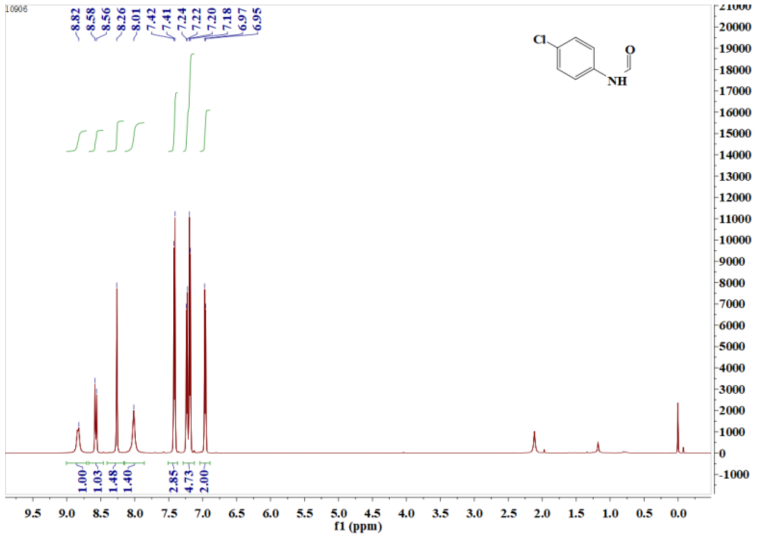

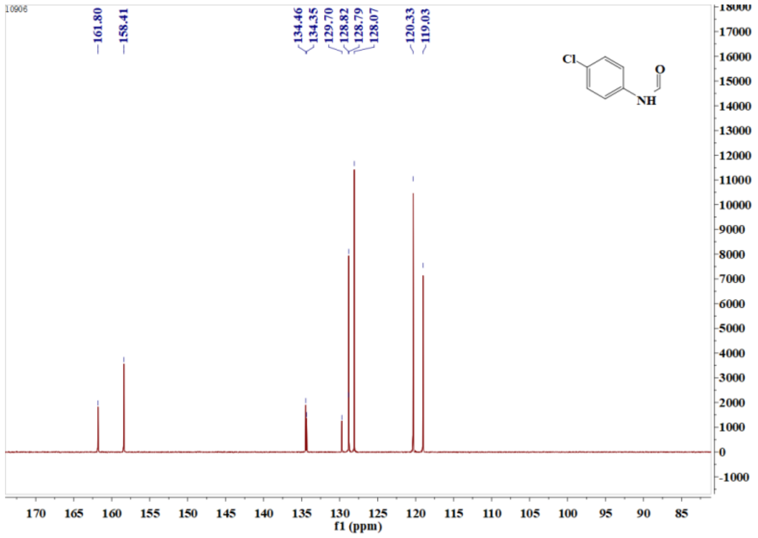

Figure S49. ^{1}H NMR ($CDCl_3$, 500 MHz) spectrum and ^{13}C NMR ($CDCl_3$, 125 MHz) spectrum of the *N*-(4-chlorophenyl)formamide.

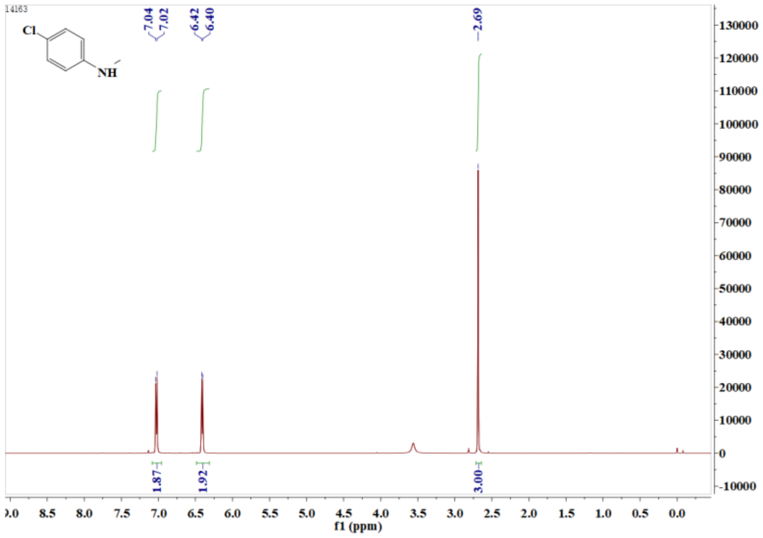

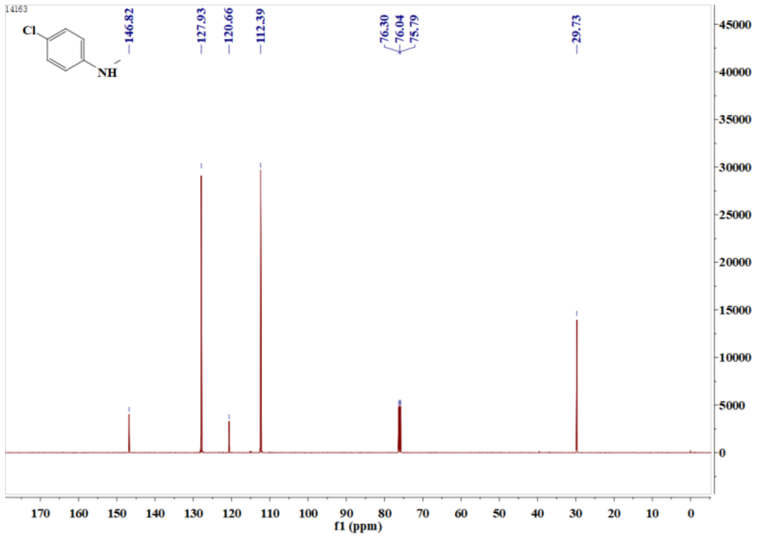

Figure S50. ^{1}H NMR ($CDCl_3$, 500 MHz) spectrum and ^{13}C NMR ($CDCl_3$, 125 MHz) spectrum of the 4-chloro-*N*-methylaniline.

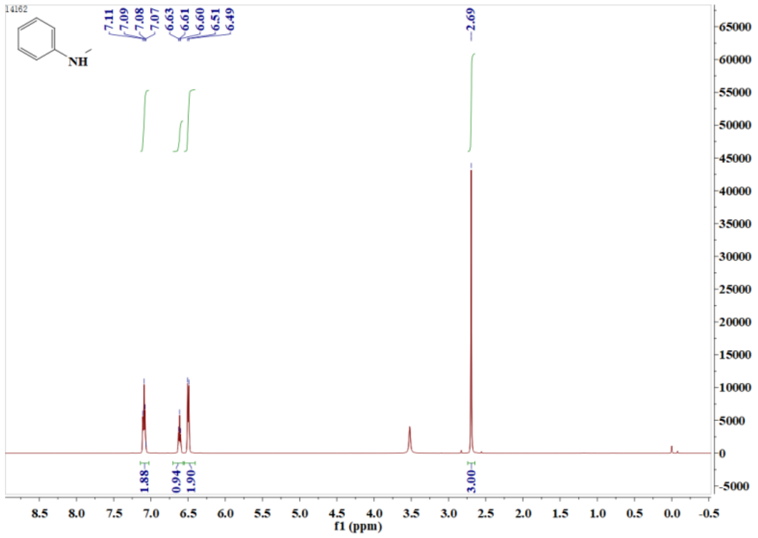

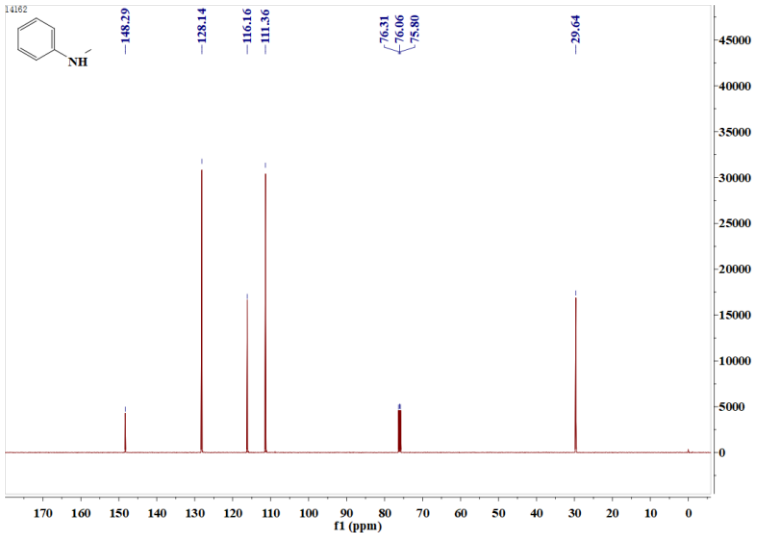

Figure S51. ^{1}H NMR ($CDCl_3$, 500 MHz) spectrum and ^{13}C NMR ($CDCl_3$, 125 MHz) spectrum of the *N*-methylaniline.

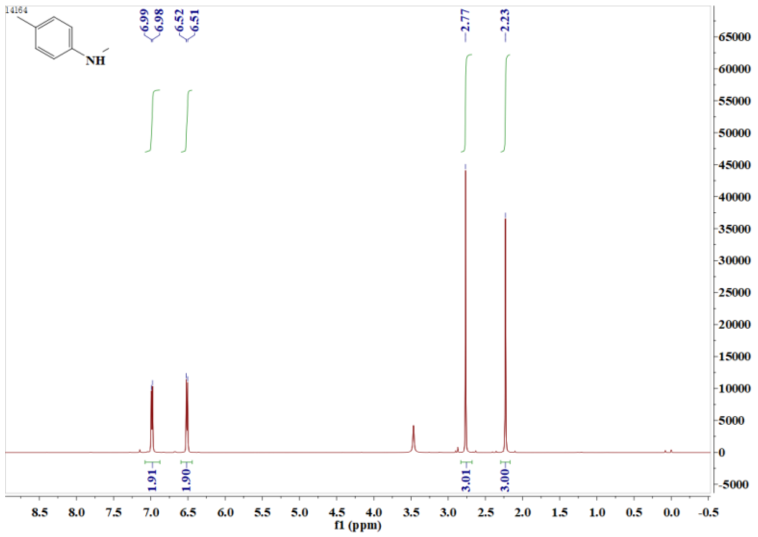

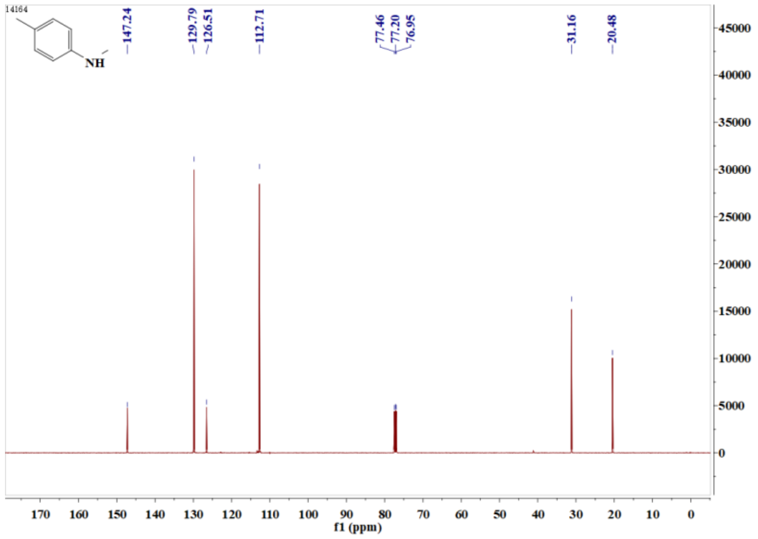

Figure S52. ^{1}H NMR ($CDCl_3$, 500 MHz) spectrum and ^{13}C NMR ($CDCl_3$, 125 MHz) spectrum of the *N*,4-dimethylaniline.
